# New pyrimidine derivatives as potential agents against hepatocellular carcinoma: design, synthesis, and *in vitro* and *in vivo* biological evaluations

**DOI:** 10.3389/fphar.2026.1745214

**Published:** 2026-01-30

**Authors:** Jeanluc Bertrand, Ignacio Montorfano, Ramón Pérez-Castro, Ricardo Valdés-Valdés, Jacqueline Romero, Thalía Delgado, Iván Brito, Juan F. Santibáñez, Alan R. Cabrera, María Paola Vieytes, Javier Echeverría, Cristian O. Salas, César Echeverría

**Affiliations:** 1 Departamento de Química Orgánica, Facultad de Química y de Farmacia, Pontificia Universidad Católica de Chile, Santiago, Chile; 2 ATACAMA-OMICS, Laboratorio de Biología Molecular y Genómica, Facultad de Medicina, Universidad de Atacama, Copiapó, Chile; 3 In vivo Tumor Biology Research Facility, Centro Oncológico, Universidad Católica del Maule, Talca, Chile; 4 Laboratorio de Investigaciones Biomédicas, Facultad de Medicina, Universidad Católica del Maule, Talca, Chile; 5 Departamento de Química, Facultad de Ciencias Básicas, Universidad de Antofagasta, Antofagasta, Chile; 6 Group for Molecular Oncology, National Institute of Republic of Serbia, University of Belgrade, Institute for Medical Research, Belgrade, Serbia; 7 Departamento de Química Inorgánica, Facultad de Química y de Farmacia, Pontificia Universidad Católica de Chile, Santiago, Chile; 8 Departamento de Ciencias del Ambiente, Facultad de Química y Biología, Universidad de Santiago de Chile, Santiago, Chile; 9 Instituto de Ciencias Naturales, Facultad de Medicina Veterinaria y Agronomía, Universidad de Las Américas, Santiago, Chile; 10 Centro de Investigación en Ciencias Biológicas y Químicas, Universidad de Las Américas, Santiago, Chile

**Keywords:** apoptosis, caspases, cytotoxicity, hepatocellular carcinoma, *in vivo* evaluation, pyrimidine derivatives

## Abstract

**Background:**

Sorafenib is a tyrosine kinase inhibitor (TKI) used to treat hepatocellular carcinoma (HCC), but this drug causes clinically significant toxicities in approximately 50% of patients. Given the high frequency and severity of these side effects, it is necessary to develop new, safer drugs to treat this cancer.

**Purpose:**

Novel 2,6,9-trisubstituted pyrimidine derivatives were synthesised and evaluated as potential antitumour agents for HCC.

**Materials and Methods:**

Twelve compounds (**6a–l**) were obtained by a four-step synthetic procedure using a simple and efficient methodology in which two key reactions were promoted by microwave irradiation. Subsequently, compounds **6a–l** were evaluated *in vitro* for cytotoxic activity against the HCC cell line HepG2 and other cell lines; *in vivo* in the HepG2 xenograft tumour model; and *in silico* (docking and dynamic simulations).

**Results and discussion:**

Compound **6e** proved to be the most promising of this series (IC_50_ = 5.6 µM), as well as being more index selective than sorafenib and with lower cytotoxicity in Vero cells (18.92 µM). In addition, **6e** was further evaluated in Huh-7 cells and demonstrated selectivity for HCC. Docking studies on the proposed targets, VEGFR-2 and B-raf, indicated that **6e** could bind to them with binding energies and interaction patterns similar to those of sorafenib. The **6e** interaction pattern at the VEGFR-2 binding site was corroborated by dynamic studies over 100 ns. A possible mechanism of **6e**-induced HepG2 cell death was investigated. Experiments on caspases-3, -7, -8, -9, Apaf-1, Cyt-c, ERK1/2, and p53 showed that they were all activated, whereas Bcl-2 was inhibited by **6e** in HepG2 cells. Furthermore, **6e** induced the accumulation of reactive oxygen species (ROS) in HepG2 cells. These results suggest that apoptosis in HepG2 was caused by: (i) a caspase-dependent pathway and (ii) changes in the cellular levels of Bcl-2 family proteins and ROS. In addition, **6e** attenuated the growth of HepG2 xenograft tumours in mice at a dose of 1 mg/kg for 3 weeks.

**Conclusion:**

Based on these results, this pyrimidine derivative could be an interesting compound for the design of new agents against HCC.

## 1 Introduction

Cancer encompasses a group of related diseases characterised by the uncontrolled division of cells and their subsequent spread to other tissues within the body (Hanahan and Weinberg, 2000). According to the World Health Organization (WHO), cancer ranks as the second leading cause of mortality globally, following cardiovascular diseases (Siegel et al., 2019). An analysis by Sung et al. of the global cancer burden in 2020, based on GLOBOCAN data, estimated 19.3 million new cases and 10 million cancer-related deaths across 36 cancer types in 185 countries (Sung et al., 2021). Among these, liver cancer is identified as the sixth most frequently diagnosed cancer and the third leading cause of cancer-related mortality, following lung and colorectal cancers, with approximately 906,000 new cases and 830,000 deaths. The term liver cancer primarily refers to hepatocellular carcinoma (HCC), which accounts for at least 85% of cases, and intrahepatic cholangiocarcinoma, comprising 10%–15% of cases, among other rare types (Sung et al., 2021).

**FIGURE 1:**
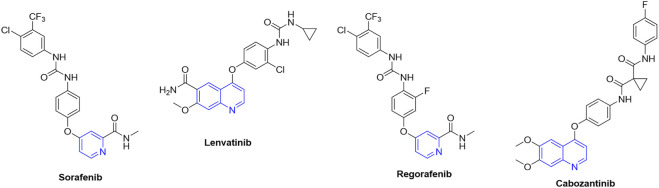
The chemical structures of TKIs are used in the clinical treatment of HCC and in clinical trials. The nitrogen-heterocyclic scaffold is highlighted in blue.

The treatment of HCC with chemotherapy presents significant challenges due to its high level of chemoresistance and the frequent presence of underlying cirrhosis in patients (Eatrides et al., 2017). Sorafenib (Figure 1), an oral tyrosine kinase inhibitor (TKI), was approved by the FDA in 2007 as a first-line treatment for patients with HCC at various stages (Kim et al., 2017; Li et al., 2019). Despite overall improvements in survival, poor tolerance to side effects in approximately 50% of patients leads to drug discontinuation (Li et al., 2019). Consequently, alternative drugs such as lenvatinib, regorafenib, and cabozantinib have been evaluated for HCC treatment (Figure 1) (Roskoski, 2021). For this reason, the pursuit of novel anticancer agents is a fundamental focus of research within medicinal chemistry and the pharmaceutical industry, aiming to identify compounds with selective antitumor activity and minimal impact on normal cells (Zhong et al., 2021).

**FIGURE 2:**
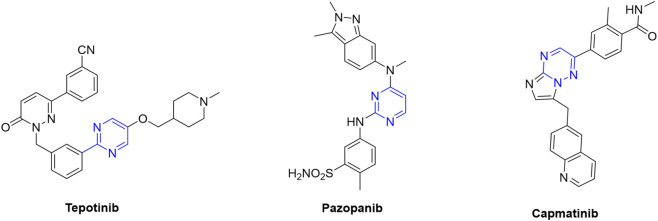
Chemical structures of pyrimidine derivatives in clinical studies of HCC treatment.

Interestingly, from a chemical perspective, most TKIs used to treat HCC share a heterocyclic ring, particularly nitrogen heterocycles such as pyridine or quinoline (Figure 1). For this reason, nitrogen-containing rings have been considered an interesting component in fragment-based cancer drug discovery (Kerru et al., 2020). In this address, considering that pyrimidine is a bioisoster of pyridine, tepotinib, capmatinib, and pazopanib (Figure 2) were developed and they are under clinical evaluation for the treatment of HCC (Le Grazie et al., 2017).

Given these antecedents, this work focused on the synthesis of new 2,4,6-trisubstituted pyrimidine derivatives and their subsequent evaluation in the HepG2 cell line, a validated model for assessing potential HCC drugs. Later, the most promising compounds were evaluated in other HCC cell lines, Huh-7, as well as in other tumoral cell lines to demonstrate selectivity, and in the non-tumoral Vero cell line as a control. *In silico* studies of VEGFR-2 and B-Raf were conducted to identify potential targets, including sorafenib. In addition, to study a possible mechanism of cell death in HepG2 cells, the apoptotic pathway, by activating caspases-3, -7, -8, and -9, apoptosis protease-activating factor-1 (Apaf-1), cytochrome c (Cyt-c), and p53, was investigated. Changes in the cellular levels of Bcl-2 family proteins and extracellular signal-regulated kinase (ERK)1/2 in HepG2 cells, as well as the induction of reactive oxygen species (ROS), were also considered. Finally, the antitumour activity of the most active compound was evaluated in a mouse HCC tumour model.

## 2 Materials and methods

### 2.1 General information

The reagents used in this study were obtained from Sigma–Aldrich (St. Louis, MO, United States). The purity of all the synthesised compounds was determined using NMR, TLC, and HRMS. In TLC, silica gel 60 F254 aluminium foils (20 × 20 cm; Merck, Burlington, United States) were used as the stationary phase, and the mobile phase was specified in each reaction procedure. In the NMR spectra, the chemical shifts of each signal were reported in parts per million (ppm) and, where appropriate, the coupling constants (*J*) were reported in hertz (*Hz*). In addition, the multiplicity of the ^1^H-NMR signals is expressed as s (singlet), d (doublet), t (triplet), and dd (doublet doublet).

The following instruments were used for the identification and characterization of each of the synthesized compounds: 1) Kofler Thermogenerate apparatus (Reichert, Werke A.G., Wien, Austria) for the determination of the melting points (m.p.), which are expressed in degrees Celsius (°C) without corrections; 2) Bruker Avance III HD-400 spectrometers [400 MHz (^1^H) and 100 MHz (^13^C)] or 200 MHz [200 MHz (^1^H) and 50 MHz (^13^C)] (Bruker, Karlsruhe, Germany) to obtain the ^1^H and ^13^C NMR spectra (tetramethylsilane, TMS, was used as internal reference); 3) Bruker Compact QTOF MS + Elute UHPLC (Bruker, Karlsruhe, Germany) with a constant nebulizer temperature of 250 °C, for the acquisition of HRMS-ESI data. This methodology was used for the final products. For this, samples dissolved in acetonitrile were injected directly into the ESI source via an injection valve and a syringe pump at a flow rate of 5 μL min^-1^. Measurements were carried out in positive ion mode, with a scanning range of *m/z* 300.00–1510.40 and a resolution of 140,000; 4) LC-MS experiments were carried out on a UHPLC Eksigent1 coupled with an MS detector ABSciex1 (AB Sciex, Woodlands Central Indus, Estate Singapore), Triple Quad 4500 model equipment. This methodology was used for the reaction intermediates. The samples were directly injected using a syringe, and the data were collected in the range of 100.0–600.0 Da, at 200 Da s^-1^ and positive polarity; 5) Bruker D8 Venture diffractometer equipped with a bidimensional CMOS Photon 100 detector, using graphite monochromated Cu-Kα (λ = 1.54178 Å) radiation (Bruker Co., Billerica, MA, United States) for the X-ray crystal structure analysis (XRD). Anisotropic displacement parameters were refined for all non-hydrogen atoms. Selected crystallographic data are listed in Supplementary Table S1 (Electronic Supplementary Material). 6) The microwave reactor CEM Discovery 101 (CEM GmbH, Kamp-Lintfort, Germany) was used to carry out chemical reactions during the synthesis of the final products. 7) HPLC-DAD was used to determine the purities of the final compounds. Chromatographic separation was performed on an ACQUITY UPLC I-Class PLUS System (Waters Corporation, Milford, MA, United States) equipped with a BEH C18 1.7 μm 2.1 × 100 mm column. Binary mobile phases were employed, with mobile phase A as 0.1% (v/v) formic acid in water and mobile phase B in acetonitrile 0.1% (v/v) formic acid in acetonitrile. The analysis was performed at a flow rate of 1.8 mL/min, with a single isocratic step lasting 5 min per run and an injection volume of 2 µL.

### 2.2 Synthetic procedures

**4-Chloro-2-methyl-6-(4-(trifluoromethoxy)phenyl)pyrimidine, 3.** In a 10 mL reaction vial, 4,6-dichloro-2-methylpyrimidine (100 mg, 0.61 mmol) and (4-(trifluoromethoxy)phenyl)boronic acid (189 mg, 0.92 mmol) in dioxane (2 mL) were added to a solution of dichlorobis(triphenyl phosphine)palladium (II) (17 mg, 0.024 mmol) and Cs_2_CO_3_ 2 M (1 mL, 2 mmol). The mixture was then stirred at 80 °C for 30 min in a microwave reactor. The mixture was cooled to room temperature, extracted with EtOAc, and the organic layer was dried over anhydrous Na_2_SO_4_. After filtration, the filtrate was concentrated and purified by silica gel chromatography using petroleum ether/EtOAc (9:1) as the mobile phase, yielding **3**. White solid, yield 40%, m.p. 35 °C–40 °C. ^1^H NMR (400 MHz, CDCl_3_) δ 8.08 (d, *J* = 8.8 Hz, 2H), 7.49 (s, 1H), 7.32 (d, *J* = 8.3 Hz, 2H), 2.75 (s, 3H) ppm. ^13^C-NMR (CDCl_3_, 101 MHz) δ 169.37, 164.27, 161.80, 151.54, 134.21, 129.06 (2C), 121.09 (2C), 120.34 (q, ^1^ *J*_C-F_ = 259.6 Hz, OCF_3_), 113.89, 25.96 ppm ^19^F NMR (CDCl_3_, 376 MHz) δ −57.70 (s, 3F) ppm. ESI/MS for (C_12_H_8_ClF_3_N_2_O [M + H]^+^). Calcd: 289.04. Found: 288.80.

**Ethyl-4-((2-methyl-6-(4-(trifluoromethoxy)phenyl)pyrimidin-4-yl)amino)benzoate, 4**. To a solution of **3** (50 mg, 0.17 mmol) and ethyl 4-aminobenzoate (24 mg, 0.144 mmol) in dioxane (2 mL), the Pd(OAc)_2_ (6 mg, 0.03 mmol), Xantphos (33 mg, 0.058 mmol) and 2 M aq Cs_2_CO_3_ (0.15 mL, 0.3 mmol) were added, then the mixture was stirred at 100 °C by 1 h in a MW reactor. After cooling to room temperature, the reaction mixture was filtered, and the filtrate was diluted with H_2_O (100 mL). The mixture was extracted with ethyl acetate, and the organic layer was dried using anhydrous Na_2_SO_4_ and concentrated. The residue was purified by silica gel chromatography using a petroleum ether/EtOAc (2:1) mobile phase to yield **4**. White solid, yield 83%, m.p. 173 °C–174 °C. ^1^H NMR (400 MHz, CDCl_3_) 8.01 (d, *J* = 8.4 Hz, 2H), 7.93 (d, *J* = 8.6 Hz, 2H), 7.46 (d, *J* = 8.4 Hz, 2H), 7.23 (d, *J* = 8.6 Hz, 2H), 6.95 (s, 1H), 4.31 (q, *J* = 7.1 Hz, 2H), 2.60 (s, 3H), 1.33 (t, *J* = 7.1 Hz, 3H) ppm. ^13^C-NMR (CDCl_3_, 101 MHz) δ 168.60, 166.29, 163.16, 160.91, 150.89, 143.23, 136.30, 131.37 (2C), 128.79 (2C), 125.55, 121.13 (2C), 120.54 (^1^ *J*_C-F_ = 258.6 Hz, OCF_3_), 119.81 (2C), 98.51, 61.05, 26.31, 14.48 ppm ^19^F NMR (CDCl_3_, 376 MHz) δ −57.74 (s, 3F) ppm. ESI/MS for (C_21_H_18_F_3_N_3_O_3_ [M + H]^+^). Calcd: 418.14. Found: 418.70.

**4-((2-Methyl-6-(4-(trifluoromethoxy)phenyl)pyrimidin-4-yl)amino)benzoic acid, 5**. To a solution of **4** (250 mg, 0.6 mmol) in MeOH (20 mL), 2 M aqueous NaOH (15 mL, 30 mmol) was added, and the mixture was stirred at room temperature for 12 h. The reaction mixture was poured into ice-cold water (30 mL). After acidification with 2 M HCl to pH 3, the precipitate was collected by filtration. The solid was washed with cold water and dried *in vacuo* to yield **5**, which was used in the next step without purification. White solid, yield 92%. ^1^H NMR (400 MHz, DMSO-*d*_*6*_) δ 11.69 (s, 1H), 8.07 (d, *J* = 8.8 Hz, 2H), 8.03–7.89 (m, 4H), 7.64 (d, *J* = 8.2 Hz, 2H), 7.42 (s, 1H), 2.72 (s, 3H).

General procedures for the synthesis of final compounds **6a–6l**

To a solution of **5** (100 mg, 0.26 mmol) and the corresponding secondary amine (50 mg, 0.31 mmol) in *N*, *N*-Dimethylformamide (DMF, 10 mL), [(Dimethylamino)-1*H*-1,2,3-triazolo-[4,5-*b*]pyridin-1-ylmethylene]-*N*-methylmethanaminium hexafluorophosphate *N*-oxide (HATU, 300 mg, 0.8 mmol) and DIPEA (0.21 mL, 0.23 mmol) were added. The mixture was then stirred at 80 °C for 16 h. After cooling to room temperature, the reaction mixture was concentrated under vacuum, and the solid residue was purified by column chromatography on silica gel using a gradient-polarity mobile phase of methanol-chloroform (0%–15%).

**(4-((2-Methyl-6-(4-(trifluoromethoxy)phenyl)pyrimidin-4-yl)amino)phenyl)(4-phenylpiperidin-1-yl)methanone, 6a**. White solid, yield 30%, m.p. 228 °C–230 °C. ^1^H NMR (400 MHz, CDCl_3_) 7.97 (d, *J* = 8.3 Hz, 2H), 7.67 (s, 1H), 7.51 (d, *J* = 8.1 Hz, 2H), 7.44 (d, *J* = 8.2 Hz, 2H), 7.30 (dt, *J* = 14.9.8 Hz, 7H), 6.92 (s, 1H), 4.88 (s, 1H), 4.03 (s, 1H), 3.03 (d, *J* = 62.3 Hz, 2H), 2.80 (t, *J* = 11.9 Hz, 1H), 2.65 (s, 3H), 1.82 (d, *J* = 78.3 Hz, 4H) ppm. ^13^C-NMR (CDCl_3_, 101 MHz) δ 170.23, 168.19, 162.49, 161.18, 150.67, 144.99, 140.46, 136.25, 131.01, 128.64 (2C), 128.40 (2C), 126.71 (2C), 126.62 83 (2C), 120.96 (2C), 120.62 (2C), 120.42 (q,^1^ *J*_*C-F*_ = 258.6 Hz, OCF_3_), 98.52, 42.76 (2C), 33.34, 26.19 (2C) ppm. ^19^F NMR (CDCl_3_, 376 MHz) δ −57.69 (s, 3F) ppm. HPLC: purity 97.95% and Rt = 2.583 min. HRMS for (C_30_H_27_F_3_N_4_O_2_ [M + H]^+^). Calcd: 533.2159. Found: 533.2158.

**(4-((2-Methyl-6-(4-(trifluoromethoxy)phenyl)pyrimidin-4-yl)amino)phenyl)(4-morpholinopiperidin-1-yl)methanone, 6b**. Yellow solid, yield 54%, m.p. 135 °C–137 °C. ^1^H NMR (400 MHz, CDCl_3_) δ 7.80 (d, *J* = 8.3 Hz, 2H), 7.40 (s, 1H), 7.32 (d, *J* = 7.7 Hz, 2H), 7.22 (d, *J* = 7.9 Hz, 2H), 7.11 (d, *J* = 8.3 Hz, 2H), 6.74 (s, 1H), 4.51 (s, 1H), 3.75 (s, 1H), 3.56 (s, 4H), 2.80 (s, 2H), 2.47 (s, 3H), 2.39 (s, 4H), 2.29 (t, *J* = 9.7 Hz, 1H), 1.74 (s, 2H), 1.33 (s, 2H) ppm. ^13^C-NMR (CDCl_3_, 101 MHz) δ 170.02, 168.26, 162.64, 161.14, 150.66, 140.46, 136.31, 130.86, 128.63 (2C), 128.39 (2C), 120.96 (2C), 120.58 (2C), 120.41 (q, ^1^ *J*_*C-F*_ = 258.6 Hz, OCF_3_), 98.35, 67.14 (2C), 61.87 (2C), 49.82 (2C), 28.86 (2C), 26.21 ppm ^19^F NMR (CDCl_3_, 376 MHz) δ −57.71 (s, 3F) ppm. HPLC: purity 98.78% and Rt = 0.867 min. HRMS for (C_28_H_30_F_3_N_5_O_3_ [M + H]^+^). Calcd: 542.2371. Found: 542.2374.

**(4-((2-Methyl-6-(4-(trifluoromethoxy)phenyl)pyrimidin-4-yl)amino)phenyl)(4-phenylpiperazin-1-yl)methanone, 6c**. Light brown solid, yield 36%, m.p. 250 °C–252 °C. ^1^H NMR (400 MHz, DMSO-*d*_*6*_) δ 9.87 (s, 1H), 8.15 (d, *J* = 8.6 Hz, 2H), 7.85 (d, *J* = 8.3 Hz, 2H), 7.51 (d, *J* = 8.3 Hz, 2H), 7.45 (d, *J* = Hz, 2H), 7.23 (t, *J* = Hz, 2H), 7,13 (s, 1H), 6.96 (m, 2H), 6.81 (m, 1H), 3.66 (s, 4H), 3.18 (s, 4H), 2.57 (s, 3H) ppm. ^13^C-RMN (DMSO-*d*_*6*_, 101 MHz) δ 168.97, 167.03, 161.05, 160.22, 150.80, 149.67, 141.45, 136.21, 128.97 (2C), 128.85 (2C), 128.58 (2C), 128.31 (2C), 121.2 (2C), 120.41 (q, ^1^ *J*_C-F_ = 249.7 Hz, OCF_3_), 118.77 (2C), 115.90 (2C), 99.86, 48.57 (2C), 26.13 ppm ^19^F NMR (CDCl_3_, 376 MHz) δ −56.65 (s, 3F) ppm. HPLC: purity 98.38% and Rt = 2.363 min. HRMS for (C_29_H_26_F_3_N_5_O_2_ [M + H]^+^). Calcd: 534.2111. Found: 534.2109.

**(4-((2-Methyl-6-(4-(trifluoromethoxy)phenyl)pyrimidin-4-yl)amino)phenyl)(4-(pyridin-2-yl)piperazin-1-yl)methanone, 6d**. White solid, yield 37%; m.p. 236 °C–239 °C. ^1^H NMR (400 MHz, CDCl_3_) δ 8.00 (d, *J* = 4.0 Hz, 1H), 7.76 (d, *J* = 8.7 Hz, 2H), 7.61 (s, 1H), 7.39–7.28 (m, 3H), 7.25 (d, *J* = 8.4 Hz, 2H), 7.08 (d, *J* = 7.8 Hz, 2H), 6.71 (s, 1H), 6.48 (dd, *J* = 10.5, 5.9 Hz, 2H), 3.46 (d, *J* = 46.0 Hz, 8H), 2.45 (s, 3H) ppm. ^13^C-RMN (CDCl_3_, 101 MHz) δ 170.46, 168.32, 162.67, 161.20, 159.19, 150.75, 148.09, 140.96, 137.85, 136.36, 130.24, 128.76 (2C), 128.72 (2C), 121.06 (2C), 120.54 (2C), 120.52 (q, ^1^ *J*_C-F_ = 254.5 Hz, OCF_3_), 114.23, 107.50, 98.67, 45.59 (2C), 29.78 (2C), 26.29 ppm ^19^F NMR (CDCl_3_, 376 MHz) δ −56.71 (s, 3F) ppm. HPLC: purity 100% and Rt = 2.630 min. HRMS for (C_28_H_25_F_3_N_6_O_2_ [M + H]^+^). Calcd: 535.2064. Found: 535.2063.

**(4-((2-Methyl-6-(4-(trifluoromethoxy)phenyl)pyrimidin-4-yl)amino)phenyl)(4-(pyridin-4-yl)piperazin-1-yl)methanone, 6e**. White solid, yield 15%, m.p. 231 °C–232 °C. ^1^H NMR (400 MHz, DMSO-*d*_*6*_) δ 10.03 (s, 1H), 8.12 (s, 2H), 8.08 (d, *J* = 8.4 Hz, 2H), 7.82 (d, *J* = 8.2 Hz, 2H), 7.44 (d, *J* = 8.2 Hz, 2H), 7.39 (d, *J* = 8.2 Hz, 2H), 7.15 (s, 1H), 6.77 (d, *J* = 4.7 Hz, 2H), 3.57 (s, 4H), 2.50 (s, 3H) ppm. ^13^C-RMN (DMSO-*d*_*6*_, 101 MHz) δ 169.6, 167.48, 161.57, 160.61, 154.77 (2C), 150.17, 142.13 (2C), 136.70 (2C), 129.03 (2C), 128.79 (2C), 121.62 (2C), 120.49 (q, ^1^ *J*_C-F_ = 262.6 Hz, OCF_3_), 119.24 (2C), 108.92, 100.48, 45.86 (2C), 26.62 ppm ^19^F NMR (CDCl_3_, 376 MHz) δ −57.65 (s, 3F) ppm. HPLC: purity 100% and Rt = 0.863 min. HRMS for (C_28_H_25_F_3_N_6_O_2_ [M + H]^+^). Calcd: 535.2064. Found: 535.2063.

**(4-((2-Methyl-6-(4-(trifluoromethoxy)phenyl)pyrimidin-4-yl)amino)phenyl)(4-(pyrazin-2-yl)piperazin-1-yl)methanone, 6f**. White solid, yield 43%, m.p. 243 °C–244 °C. ^1^H NMR (400 MHz, DMSO-*d*_*6*_) δ 9.87 (s, 1H), 8.31 (d, *J* = 11.4 Hz, 1H), 8.17–7.95 (m, 3H), 7.84 (d, *J* = 8.6 Hz, 3H), 7.47 (dd, *J* = 19.6, 8.3 Hz, 4H), 7.12 (s, 1H), 3.63 (s, 8H), 2.55 (s, 3H) ppm. ^13^C-RMN (DMSO-*d*_*6*_, 101 MHz) δ 169.65, 167.49, 161.52, 160.67, 154.92, 150.15, 141.92, 136.66, 133.24, 131.93, 129.06 (2C), 128.82 (2C), 121.61 (2C), 119.25 (2C), 100.36, 79.64, 44.42 (2C), 26.59 ppm ^19^F NMR (CDCl_3_, 376 MHz) δ −56.65 (s, 3F) ppm. HPLC: purity 100% and Rt = 2.033 min. HRMS for (C_27_H_24_F_3_N_7_O_2_ [M + H]^+^). Calcd: 536.2016. Found: 536.2000.

**(4-((2-Methyl-6-(4-(trifluoromethoxy)phenyl)pyrimidin-4-yl)amino)phenyl)(4-(5-(trifluoromethyl)pyridin-2-yl)piperazin-1-yl)methanone, 6g**. Light brown solid, yield 23%, m.p. 199 °C–202 °C. ^1^H NMR (400 MHz, CDCl_3_) δ 8.35 (s, 1H), 7.91 (d, *J* = 8.7 Hz, 2), 7.64–7.53 (m, 2H), 7.49 (d, *J* = 8.5 Hz, 2H), 7.40 (d, *J* = 8.5 Hz, 2H), 7.22 (d, *J* = 8.4 Hz, 2H), 6.86 (s, 1H), 6.60 (d, *J* = 9.0 Hz, 1H), 3.65 (s, 8H), 2.59 (s, 3H) ppm. ^13^C-RMN (CDCl_3_, 101 MHz) δ 170.39, 168.27, 162.72, 161.04, 160.10, 150.73, 145.77 (d, ^3^ *J*_C-F_ = 4.0 Hz), 140.87, 136.19, 134.76, 130.05, 128.75 (2C), 128.65 (2C), 122.38 (q, ^1^ *J*_C-F_ = 295.3 Hz, OCF_3_), 120.98 (2C), 120.52 (2C), 116.07 (d, ^3^ *J*_C-F_ = 33.3 Hz), 105.83, 98.43, 44.80 (2C), 30.92 (2C), 25.15 ppm ^19^F NMR (CDCl_3_, 376 MHz) δ −57.71 (s, 3F), −61.22 (s, 3F) ppm. HPLC: purity 95.57% **(4-((2-Methyl-6-(4-(trifluoromethoxy)phenyl)pyrimidin-4-yl)amino)phenyl)(4-(4-nitrophenyl)piperazin-1-yl)methanone, 6h**. Yellow solid, yield 76%, m.p. 150 °C–153 °C. ^1^H NMR (400 MHz, CDCl_3_) δ 8.06 (d, *J* = 9.3 Hz, 2H), 7.92 (d, *J* = 8.7 Hz, 2H), 7.50 (d, *J* = 8.4 Hz, 2H), 7.42 (d, *J* = 8.4 Hz, 2H), 7.22 (d, *J* = 8.0 Hz, 3H), 6.85 (s, 1H), 6.76 (d, *J* = 9.3 Hz, 2H), 3.76 (s, 4H), 3.40 (s, 4H), 2.59 (s, 3H) ppm. ^13^C-RMN (CDCl_3_, 101 MHz) δ 170.29, 168.36, 162.86, 160.96, 154.49, 150.76, 140.91, 139.24, 136.19, 129.86, 128.86 (2C), 128.66 (2C), 125.96 (2C), 121.00 (2C), 120.48 (2C), 120.41 (q, ^1^ *J*_C-F_ = 258.6 Hz, OCF_3_), 113.20 (2C), 98.35, 47.19 (2C), 30.52 (2C), 26.21 ppm ^19^F NMR (CDCl_3_, 376 MHz) δ −57.70 (s, 3F) ppm. HPLC: purity 98.53% Rt = 1.907. HRMS for (C_29_H_25_F_3_N_6_O_4_ [M + H]^+^). Calcd: 579.1962. Found: 579.1975.

**(4-(4-Chlorophenyl)piperazin-1-yl)(4-((2-methyl-6-(4-(trifluoromethoxy)phenyl)pyrimidin-4-yl)amino)phenyl)methanone, 6i**. Light brown solid, yield 63%, m.p. 223 °C–225 °C. ^1^H NMR (400 MHz, CDCl_3_) δ 7.99 (d, *J* = 8.3 Hz, 2H), 7.72 (s, 1H), 7.58 (d, *J* = 8.1 Hz, 2H), 7.47 (d, *J* = 8.1 Hz, 2H), 7.30 (d, *J* = 9.4 Hz, 2H), 7.25 (d, *J* = 8.5 Hz, 2H), 6.98 (s, 1H), 6.87 (d, *J* = 8.6 Hz, 2H), 3.83 (s, 4H), 3.19 (s, 4H), 2.68 (s, 3H) ppm. ^13^C-RMN (CDCl_3_, 101 MHz) δ 170.13, 168.02, 162.29, 161.00, 150.81, 149.51, 140.57, 135.77, 130.39, 129.17 (2C), 128.72 (2C), 128.70, 125.66, 120.97 (2C), 120.66 (2C), 120.40 (q, ^1^ *J*_C-F_ = 258.6 Hz, OCF_3_), 117.96 (2C), 98.54, 49.74 (2C), 29.70 (2C), 25.99 ppm ^19^F NMR (CDCl_3_, 376 MHz) δ −57.69 (s, 3F) ppm. HPLC: purity 100% and Rt = 2.860 min. HRMS for (C_29_H_25_ClF_3_N_5_O_2_ [M + H]^+^). Calcd: 568.1722. Found: 568.1725.

**(4-(4-Methoxyphenyl)piperazin-1-yl)(4-((2-methyl-6-(4-(trifluoromethoxy)phenyl)pyrimidin-4-yl)amino)phenyl)methanone, 6j**. White solid, yield 40%, m.p. 207 °C–209 °C. ^1^H NMR (400 MHz, CDCl_3_) δ 7.99 (d, *J* = 8.7 Hz, 2H), 7.49 (q, *J* = 8.6 Hz, 4H), 7.29 (d, *J* = 8.3 Hz, 2H), 6.95–6.89 (m, 3H), 6.86 (d, *J* = 9.1 Hz, 2H), 3.78 (s, 7H), 3.09 (s, 4H), 2.66 (s, 3H) ppm. ^13^C-RMN (CDCl_3_, 101 MHz) δ 170.16, 168.43, 163.00, 161.21, 154.64, 150.87, 145.32, 140.56, 136.37, 130.86, 128.89 (2C), 128.81 (2C), 121.11 (2C), 120.87 (2C), 120.54 (q, ^1^ *J*_C-F_ = 272.7 Hz, OCF_3_), 119.12 (2C), 114.70 (2C), 98.22, 55.69, 51.41 (2C), 29.80 (2C), 26.25 ppm. HPLC: purity 100% and Rt = 1.813 min ^19^F NMR (CDCl_3_, 376 MHz) δ −57.71 (s, 3F) ppm. HRMS for (C_30_H_28_F_3_N_5_O_3_ [M + H]^+^). Calcd: 564.2217. Found: 564.2205.

**(4-(2,4-Dimethylphenyl)piperazin-1-yl)(4-((2-methyl-6-(4-(trifluoromethoxy)phenyl)pyrimidin-4-yl)amino)phenyl)methanone, 6k**. Light brown solid, yield 30%, m.p. 224 °C–225 °C. ^1^H NMR (400 MHz, CDCl_3_) δ 8.24 (d, *J* = 8.5 Hz, 2H), 7.94 (s, 1H), 7.79 (d, *J* = 8.2 Hz, 2H), 7.73 (d, *J* = 8.2 Hz, 2H), 7.58–7.49 (d, *J* = 8.3 Hz, 2H), 7.29 (s, 1H), 7.24 (d, *J* = 10.2 Hz, 2H), 7.16 (d, *J* = 8.0 Hz, 1H), 4.05 (d, *J* = 59.3 Hz, 4H), 3.16 (s, 4H), 2.92 (s, 3H), 2.56 (s, 3H), 2.54 (s, 3H) ppm. ^13^C-RMN (CDCl_3_, 101 MHz) δ 170.11, 168.02, 162.42, 161.08, 150.81, 148.34, 140.23, 135.85, 133.41, 132.66, 131.96, 131.08, 128.74 (2C), 128.70 (2C), 127.18 (2C), 120.87 (2C), 120.50 (q, ^1^ *J*_C-F_ = 259.6 Hz, OCF_3_), 119.18 (2C), 98.27, 52.19 (2C), 29.70 (2C), 25.94, 20.71, 17.64 ppm ^19^F NMR (CDCl_3_, 376 MHz) δ −57.70 (s, 3F) ppm. HPLC: purity 100% and Rt = 4.377 min. HRMS for (C_31_H_30_F_3_N_5_O_2_ [M + H]^+^). Calcd: 562.2424. Found: 562.2421.

**(4-(2-Fluorophenyl)piperazin-1-yl)(4-((2-methyl-6-(4-(trifluoromethoxy)phenyl)pyrimidin-4-yl)amino)phenyl)methanone, 6l**. White solid, yield 21%, m.p. 230 °C–232 °C. ^1^H NMR (400 MHz, DMSO-*d*_*6*_) δ 9.87 (s, 1H), 8.15 (d, *J* = 8.5 Hz, 2H), 7.85 (d, *J* = 8.2 Hz, 2H), 7.52 (d, *J* = 8.3 Hz, 2H), 7.46 (d, *J* = 8.2 Hz, 2H), 7.20–6.94 (m, 4H), 3.69 (s, 4H), 3.05 (s, 4H), 2.57 (s, 3H) ppm. ^13^C-RMN (DMSO-*d*_*6*_, 101 MHz) δ 169.45, 167.51, 161.52, 160.72, 156.70, 154.27, 150.16, 141.93, 140.02, 136.71, 129.28 (2C), 129.06 (2C), 128.81 (2C), 125.34, 123.30, 121.63 (2C), 120.09 (2C), 119.25, 116.46, 100.33, 50.83 (2C), 26.61 ppm ^19^F NMR (CDCl_3_, 376 MHz) δ −56.64 (s, 3F), −122.90 (s, F) ppm. HPLC: purity 100% and Rt = 0.893 min. HRMS for (C_29_H_25_F_4_N_5_O_2_ [M + H]^+^). Calcd: 552.2017. Found: 552.1996.

### 2.3 X-ray structure determination

Single-crystal X-ray diffraction data were collected on a Bruker D8-VENTURE I\μS diffractometer using graphite monochromated Mo-Kα radiation (λ = 0.71073 Å). Data were collected at 293(2) K. The intensities were corrected for absorption by empirical correction with the X-area. The structures were solved using direct methods (SHELXS) (Sheldrick, 2015) and refined by full-matrix least-squares calculations on F2 (SHELXL-97). The program OLEX2 (Dolomanov et al., 2009) was used to generate molecular graphics. H atoms were in the difference Fourier map but refined with fixed individual displacement parameters, using a riding model with C − H distances of 0.93 Å (for aromatic rings), 0.96 Å (for CH_3_ group) and 0.97 Å (for CH_2_ group) with U(H) values of 1.2 Ueq(C) (for CH in aromatic moiety and CH_2_ group), and 1.5 Ueq(C) (for CH_3_). Selected distances, angles, and dihedral angles are summarised in Supplementary Table S1 (Supplementary Material). The CCDC number of the structure is 1474904.

### 2.4 Biology

#### 2.4.1 Cell culture

HepG2, HL-60, HeLa, MCF-7, and Vero cells (American Type Culture Collection, ATCC-HB-8065, ATCC-CCL-240, ATCC-CL-2, ATCC-HTB-22, and ATCC-CCl-81) and Huh-7 (Dra. Susana Zanlungo kindly provided these cells from the Faculty of Medicine at the Pontificia Universidad Católica de Chile) were grown in monolayer culture in Dulbecco’s modified Eagle’s medium (DMEM) with 10% foetal bovine serum (FBS) (Gibco, NY, United States) and antibiotic-antimycotic (Gibco, NY, United States) at 37 °C in a humidified 5% CO_2_ incubator.

#### 2.4.2 MTT assay

The cells were seeded in a 96-well plate at an initial density of 5 × 10^3^ cells/well. 24 h later, the cells were treated with control (Milli-Q water) or various concentrations of **6e** for 24 h. Following treatment, the cells were incubated with 0.5 mg/mL MTT tetrazolium for 4 h at 37 °C. The resulting violet formazan precipitate was solubilised in DMSO after gentle shaking for 10 min. The absorbance of dissolved formazan crystals was measured at 540 nm using a microplate reader (Tecan Infinite ® f200, Grodig, Austria) (van Meerloo et al., 2011; Echeverría et al., 2022).

#### 2.4.3 Annexin-V/PI apoptosis assay

Apoptosis was measured through flow cytometry. Detection of cell externalisation of phosphatidylserine on early apoptotic cells using fluorescein-labelled Annexin V with the “Alexa Fluor 488 Annexin V/propodium iodide (PI) Dead Cell Apoptosis” Kit. The protocol was performed according to the manufacturer’s instructions (Invitrogen, Carlsbad, CA, United States). The cells were analysed on a flow cytometer (BD Accuri C6), and 10,000 events were counted per sample (Kumar et al., 2021; Salem et al., 2023).

#### 2.4.4 Fluorescence microscopy

The HepG2 cells were washed twice with PBS before being fixed. Subsequently, the cells were washed once more and incubated with primary antibodies. The cells were washed twice and treated with secondary antibodies. The samples were then mounted using the ProLong Gold antifade mounting medium containing DAPI (Invitrogen, UK) (Koch-Edelmann et al., 2017; Im et al., 2019; Echeverría et al., 2022). For a detailed list of the antibodies used, see Supplementary Table S2. Fluorescent cells were analysed using an EVOS ® FLoid ® cell microscope (Life Technologies, CA, United States).

#### 2.4.5 Western blot

HepG2 cells were lysed with cold lysis buffer, and then proteins were extracted. Supernatants were collected and preserved in lysis buffer. Both the protein extract and supernatant were subjected to sodium dodecyl sulfate polyacrylamide gel electrophoresis (SDS-PAGE), and the separated proteins were transferred onto nitrocellulose or polyvinylidene fluoride (PVDF) membranes. After blocking, the membrane was incubated with the appropriate primary antibody, washed twice, and then incubated with a secondary antibody. The bands were detected using a peroxidase-linked IgG antibody. Tubulin and α-actin served as a loading control (Mahmood and Yang, 2012; Echeverría et al., 2022). A detailed list of the antibodies used is provided in Supplementary Table S3.

#### 2.4.6 Caspase assays

The Caspase-Glo 3/7 assay (Promega, Madison, WI, United States) was used to measure the activity of the key effector caspases 3 and 7 according to the manufacturer’s instructions (https://worldwide.promega.com/products/cell-health-assays/apoptosis-assays/caspase_glo-3_7-assay-systems/?tabset0=0). Briefly, cells were seeded at a density of 2000 cells/well in 96-well white-walled plates. The next day, cells were treated with compounds. Cleaved caspase 3/7 activity was assessed at 4 and 8 h. Caspase-Glo reagent was added to the wells, carefully mixed, and incubated in the dark at room temperature for 90 min. Absorbance and luminescence signals were measured using a microplate reader (Tecan infinite® M200 Pro, Männedorf, Switzerland). Each treatment was replicated at least 4 times (Alzamami et al., 2023).

#### 2.4.7 Determination of intracellular ROS

2,7-Dichlorodihydrofluorescein diacetate (DCFH-DA) is a cell-permeable probe used to detect intracellular ROS. Inside the cell, it is converted to DCF-DA by esterases and quickly oxidised to highly fluorescent DCF in the presence of hydrogen peroxide and peroxidases (Echeverría et al., 2022). Cells were treated with 10 μM DCFH-DA for 30 min and subsequently rinsed with PBS. DCF fluorescence was detected using a fluorescence plate reader (Tecan infinite® M200Pro). ROS levels were expressed as relative fluorescence units (RFU) of DCF (Kim and Xue, 2020).

### 2.5 *In silico* study

#### 2.5.1 Molecular docking

The crystallographic structure of VEGFR-2 bound to sorafenib (2.03 Å) was retrieved from the Protein Data Bank database (PDB code 4ASD). The UCSF Chimera program was used to remove the co-crystallised ligands and other molecules (Pettersen et al., 2004). Polar hydrogen addition and side-chain reparation were performed using the Chimera « DockPrep » command. Considering the physiological pH, the protein’s protonation state is limited to the histidine residue. Compound **6e** was constructed using ChemDraw to obtain the SMILES code. The protonation states, energy minimisation, and generation of the three-dimensional structure were performed using OpenBabel (O’Boyle et al., 2011).

Molecular docking was performed using RDOCK (Li et al., 2003). The receptor (VEGFR-2) in mol2 format was used to run the software. The cavity was initially created using the co-crystallised ligand with a total volume of 1383.62 Å^3^ and centred at coordinates X = −23.5526, Y = −0.818728, and Z = −11.1295. The docking protocol was quantitatively validated by re-docking the co-crystallised sorafenib into the VEGFR-2 binding site. Multiple independent fifty docking runs were performed, and the best-ranked pose reproduced the experimental binding mode with a root-mean-square deviation (RMSD) of 1.02 Å relative to the crystallographic structure and a docking score of −28.85. RMSD values below 2.0 Å are generally accepted as indicative of successful pose reproduction, thereby confirming the protocol’s ability to recover the experimentally observed binding mode reliably. The re-docked ligand was consistently ranked among the top-scoring poses across independent runs (Supplementary Figure S1). Then, fifty conformations were generated for the ligand, and the top five ranked conformations, determined by the docking score, were chosen. Following molecular docking, we scrutinised the optimised binding poses, generated graphical representations in PyMOL, and used the Protein-Ligand Interaction Profiler (PLIP) to analyse non-covalent interactions within the ligand-receptor complex (Schrödinger, 2010; Adasme et al., 2021). An identical methodology was employed for the B-Raf kinase (PDB ID: 1UWH), obtained from the Protein Data Bank with a resolution of 2.95 Å. The cavity was initially created using the co-crystallised ligand with a total volume of 1542.12 Å^3^ and centred at coordinates X = 73.7531, Y = 44.7420, and Z = −64.6680. The docking protocol was quantitatively validated by re-docking the co-crystallised sorafenib into the B-Raf binding site. Across 50 independent docking runs, the highest-ranked pose reproduced the experimental binding mode with an RMSD of 1.17 Å relative to the crystallographic structure and a docking score of −30.61, indicating adequate protocol performance.

#### 2.5.2 Molecular dynamics

The best pose of each molecule was subjected to molecular dynamics simulations using the Desmond module within Maestro. Solvation was performed using the Transferable Intermolecular Potential 3-Point water model (TIP3P). Counterions (Na^+^) were added to neutralise the net charge, and 0.15 M NaCl was included to mimic physiological ionic strength. All systems used the Optimised Potentials for Liquid Simulations, version 3e (OPLS3e) force field. Each trajectory was run for 100 ns under NPT conditions at 310 K and 1 atm. Trajectories were recorded every 100 ps, yielding ∼1000 frames per system.

### 2.6 *In vivo* study

#### 2.6.1 Animals

Immunodeficient NSG™ mice male/female (Jackson Laboratory, Bar Harbour, ME, United States) were bred and housed in individually ventilated cages (IVC) under specific pathogen-free conditions in the *in vivo* Tumour Biology Research Facility of Centro Oncológico at the Universidad Católica Del Maule (UCM), Chile. The Institutional Animal Care and Use Committee of the UCM approved all animal experiments.

#### 2.6.2 Tumour xenograft assays

Under isoflurane anaesthesia, 5 x10^6^ HepG2 cells suspended in 100 µL of PBS were injected subcutaneously into the dorsal flank of 5 week-old NSG mice (Jung, 2014; Echeverría et al., 2022). The mice were weighed weekly, and tumour growth was tracked using a digital calliper to calculate tumour volume as (length × width^2^)/2 (Carlsson et al., 1983). When tumours reached a size of 80–100 mm^3^, the mice were randomly divided into different experimental groups (*n* = 6). They received intraperitoneal injections (sodium chloride containing 2.5% Tween-80% and 2.5% ethanol, in a final volume of 100 μL) of 1 and 5 mg/kg **6e**, or vehicle (PBS), twice a week for 3 weeks. Throughout the treatment period, the mice were observed daily, and their weight and tumour volume were measured twice a week (Echeverría et al., 2022; El-Zend et al., 2025).

#### 2.6.3 Data analysis

In all experiments, replicates were performed, and the data presented represent the mean ± standard error (SEM) from at least three independent experiments, each performed in triplicate. Comparisons were conducted using one-way analysis of variance (ANOVA) (Kruskal–Wallis), followed by Dunn’s post hoc test, and statistical significance was set at *p* < 0.05.

## 3 Results

### 3.1 Design and synthesis

**FIGURE 3:**
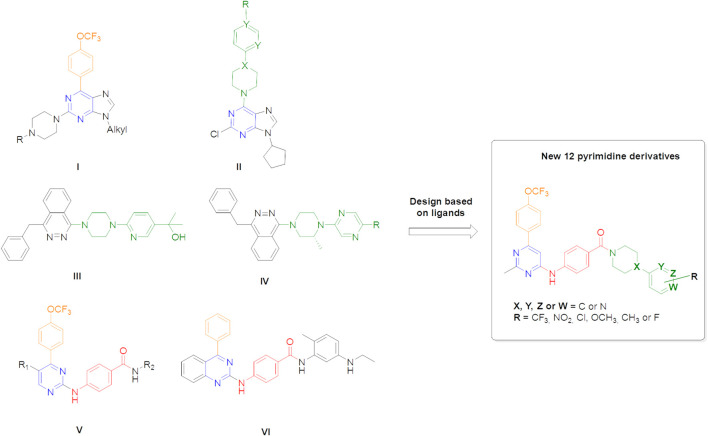
Design of new pyrimidine derivatives as potential agents for HCC treatment.

As mentioned in the introduction, the pyrimidine heterocycle is an attractive scaffold for drug design and the development of new anticancer drugs (Kaur et al., 2014; de la Torre and Albericio, 2019; He et al., 2020), and accordingly, in this work, the design of these compounds was based on our previous results of some 2,6,9-trisubstituted purine derivatives in several cancer cell lines (**I–II**, Figure 3) (Salas et al., 2019; Bertrand et al., 2020; 2022; Zárate et al., 2021), as well as several nitrogenated antitumoral agents (**III–VI**, Figure 3) (Siu et al., 2009; Peukert et al., 2013; Xin et al., 2014). First, the trifluoromethoxyphenyl fragment at C-6 was retained, as it has been shown to favour antitumour activity (orange fragment in Figure 3). Second, the *p*-aminobenzoic moiety (red fragment in Figure 3), linked to C-4 of the pyridine ring, was extended by piperazine or piperidine to an aromatic ring or morpholine to analyse the influence of the substitution with several fragments found in bioactive pyrimidine, purine, and pyrimidine analogues (Figure 3, green colour these fragments).

**FIGURE 4:**
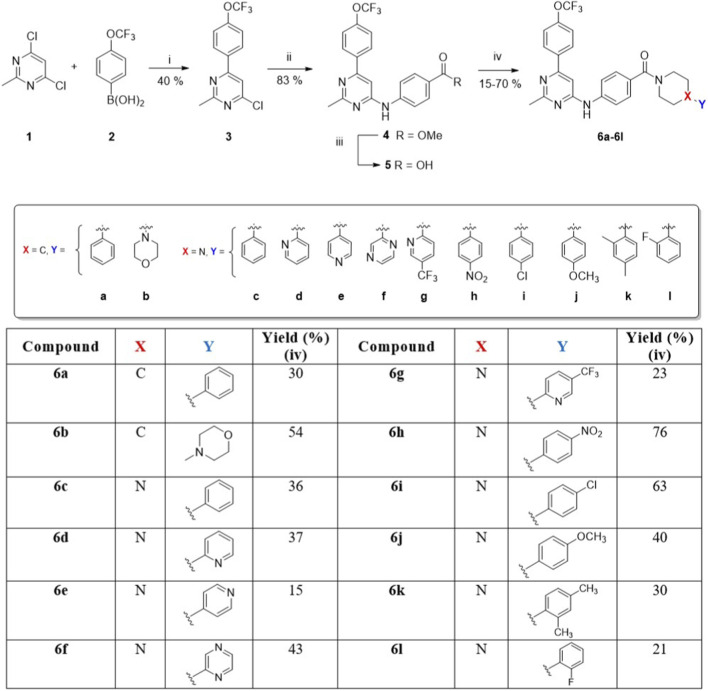
Reagents and conditions: i) Pd(PPh_3_)_2_Cl_2_, Cs_2_CO_3_, dioxane, 80 °C, MW, 30 min, 40%; ii) Ethyl 4-aminobenzoate, Pd(OAc)_2_, XantPhos, Cs_2_CO_3_ 2M, dioxane, 100 °C, MW, 1 h, 83%; iii) aq NaOH 2 M/methanol, rt, 12 h. Later, aq HCl 10%, pH ≈ 3, 92%; iv) Piperidines/piperazines, HATU, DIPEA, DMF, 80 °C, 16 h.

**FIGURE 5:**
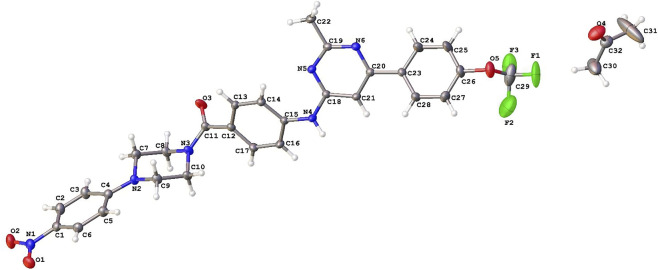
Molecular structure of **6h** obtained by XRD. Thermal ellipsoids are shown at a 30% probability.

The syntheses and structures of the new 2,6,9-trisubstituted pyrimidines **6a–l** are described in Figure 4. Twelve compounds, based on the chemical structures of well-known cytotoxic agents, were synthesised in a four-step process using 4,6-dichloro-2-methylpyrimidine (**1**) as the starting material. The first step was substitution at the C-6 position of **1** with trifluoromethoxyphenylboronic acid 2 under MW irradiation for 30 min, yielding compound 3 via a Suzuki reaction (Salih and Baqi, 2019). Second, through a Buchwald-Hartwig C-N coupling reaction (Salas et al., 2019; Bertrand et al., 2020; 2022; Zárate et al., 2021; Villegas et al., 2022) between **2** and ethyl-*p*-aminobenzoate, using Pd(OAc)_2_ and XantPhos under MW conditions at 100 °C for 1 h, derivative **4** was obtained with a yield of 83%. Subsequently, the corresponding basic hydrolysis product of **4**, followed by treatment with HCl, provided carboxylic acid **5**, which was suitable for the next step (92% yield). Finally, the coupling of **5** with the selected arylpiperazines or phenylpiperidine was carried out using HATU in low to moderate yields (15%–70%). These low yields could be explained by the fact that, in this step, the value is calculated after the purification process of each final compound. The structures of the newly synthesised compounds were established based on their spectral properties (^1^H, ^13^C, and ^19^F NMR, MS, or HRMS; see the Materials and Methods section and Supplementary Material). X-ray diffraction was also performed on compound **6h** (Figure 5).

### 3.2 Crystallographic data

The molecular structure of the selected pyrimidine derivative, **6h**, was determined using single-crystal X-ray crystallography (XRD, Figure 5). Selected distances, angles, and dihedral angles are listed in Supplementary Table S1. The dihedral angles formed by the four approximately planar C atoms of the piperazine ring and the nitrobenzene, benzene, pyrimidine, and trifluoromethoxyphenyl rings are: 31.22 (19); 77.74(19); 81.57(19), and 49.2(2)°, respectively. The piperazine ring had a chair conformation. All distances and angles were expected. In the crystal, the molecules are linked by strong N—H⋅⋅⋅O hydrogen bonds into a chain with graph-set notation C(8) along the [010] direction.

### 3.3 Cytotoxicity of pyrimidine derivatives

To analyse the potential *in vitro* antitumor activity of the synthesised compounds, their cytotoxic effects were first investigated in the HepG2 cell line, which corresponds to a cell line widely used to evaluate the cytotoxic potential of new drugs for the treatment of HCC (Arzumanian et al., 2021; Blidisel et al., 2021). A conventional colourimetric assay was performed to estimate the IC_50_ values of these compounds in cells after 72 h of continuous exposure. These assays were performed in triplicate using four serial dilutions (0.1–50 µM) of each sample. Sorafenib, a reference anticancer drug for the treatment of HCC, was used as a positive control. The cytotoxicity results on HepG2 cells indicated that only compound **6e** had an IC_50_ below 50 µM (IC_50_ = 5.6 µM; see Table 1; Supplementary Table S4). To demonstrate that **6e** inhibits HCC cell growth, it was also tested in the Huh-7 cell line, which showed an IC_50_ = 11.6 µM. Similarly, **6e** was tested in other tumour cell lines (HL-60, HeLa, and MCF-7) to demonstrate toxicity against HCC and in the non-tumour Vero cell line used as a non-tumorigenic control; in these cases, we used etoposide as the positive control. In addition, the use of Vero cells allowed the calculation of the Selectivity Index (SI). This value indicates the ratio of toxicity in Vero cells to that in tumour cells. The IC_50_ and SI values of **6e** in each cell line are listed in Supplementary Table S1.

**TABLE 1:** *In vitro* cytotoxicity of **6e**, etoposide, and sorafenib on cancer cell lines and Vero cells, and the Selective Index values are shown in parentheses.

Compounds	IC_50_ values^a^ (μM)					
	HepG2	Huh-7	HL-60	HeLa	MCF-7	Vero
6e	5.6 ± 0.7 (3.4)^b^	11.6 ± 0.11 (1.6)	32 ± 2.0	>50	>50	18.92 ± 1.3
Etoposide	—	—	3.0 ± 1.1	21.2 ± 3.8	7.6 ± 2.1	>25
Sorafenib	4.1 ± 1.1 (1.6)	3.69 ± 1.34 (1.7)	—	—	—	6.6 ± 1.1

IC_50_ values were determined in three independent experiments, each performed in triplicate, across the range 0.05–50 μM b Selective index = IC_50_ value on Vero cell/IC_50_ value on cancer cell line.

As demonstrated in Table 1, the HepG2 and Huh-7 cell lines exhibited increased sensitivity to **6e**, with IC_50_ values ranging from 5.6 to 11.6 µM, whereas the HL-60, HeLa, and MCF-7 cells exhibited IC_50_ values greater than 32 µM. A statistical analysis revealed that **6e** exhibited a comparable potency to sorafenib in the HepG2 cell line. However, **6e** demonstrated approximately three times lower potency in the Huh-7 cell line than sorafenib, with IC_50_ values of 11.6 ± 0.11 µM and 3.69 ± 1.34 µM, respectively. Furthermore, **6e** showed a relatively minor effect on Vero cells compared with sorafenib, as reflected in SI values: they were similar to sorafenib in Huh-7 cells but twice as selective for HepG2 cells. Therefore, these results indicate that sorafenib lacks specificity, as it affects these three cell lines with equal potency. Consequently, given **6e**′s primary cytotoxic activity in two HCC cell lines, potential targets were first identified *in silico* and subsequently investigated *in vitro* in HepG2 cells.

### 3.4 *In silico* studies to identify targets for 6e

Sorafenib exhibits a dual mechanism of action in HCC: it blocks tumour proliferation and growth by inhibiting the RAF/MEK/ERK pathway in tumour cells. It reduces tumour angiogenesis by inhibiting VEGFR and PDGFR signalling in the tumour vasculature, where VEGFR-2 and B-Raf are key molecular targets. Therefore, as a first step, we conducted molecular docking studies on these targets, compared them with sorafenib, and subsequently validated the results using molecular dynamics simulations.

There are two existing crystal structures of proteins with sorafenib corresponding to VEGFR-2 (PDB code 4ASD (McTigue et al., 2012) and B-Raf (PDB code 1UWH (Wan et al., 2004). Interestingly, these two proteins appear as potential targets when **6e** is submitted to the target-fishing program PLATO (Ciriaco et al., 2021; 2022). A large fraction of the PLATO-fished targets are kinases, including another sorafenib target, FLT-3. Therefore, to predict whether **6e** is a ligand for VEGFR-2 and B-Raf and to propose a potential binding pocket and the most stable conformation with these kinases, molecular docking studies were performed. The complexes with the best docking poses were energy-minimised and initially ranked using the Standard Precision (SP) scoring function. The SP docking scores for **6e** and sorafenib are reported in Table 2. To validate the docking protocol, we re-docked the co-crystallised sorafenib into the VEGFR-2 binding site; the best-ranked pose reproduced the experimental conformation with an RMSD of 1.02 Å (Supplementary Table S5). These RMSD values are below 2.0 Å, which indicates successful pose reproduction in self-docking tests. According to these values, **6e** exhibited an affinity comparable to that of sorafenib for VEGFR-2 and B-raf. Still, with higher energy than sorafenib, **6e** could be a suitable ligand for these kinases.

**TABLE 2:** Binding affinity scores (kcal/mol) for **6e**, sorafenib for VEGFR-2 and B-raf, and interaction modes in the binding sites.

Compounds	VEGFR-2		B-raf	
	Score	Interactions^a^	Score	Interactions^a^
**6e**	−31.98	**Hydrophobic:** Leu34, Val42, Ala60, Lys62, Val93, Val108, Val110, Leu172, Asp183, Phe184 **H-bonds:** Lys62, Glu79, Cys113 **Other interactions:** p-cation Lys62 and halogen bond Ile162	−31.80	**Hydrophobic:** Leu58, Ile66, Leu67, Thr82, Trp84, Asp147 **H-bonds:** Glu54 and Asp147 **Other interactions:** π-stackings His127 and Phe136
**Sorafenib**	−29.37	**Hydrophobic:** Val42, Ala60, Glu79, Ile82, Leu172, Asp183, Phe184 **H-bonds:** Lys62, Glu79, Cys113 and Asp183	−29.87	**Hydrophobic:** Val24, Lys36, Glu444, Leu58, Leu67, Ile80, Phe136, asp147, Phe148 **- H-bonds:** Glu54, Cys85 and Asp147 **- Other interactions:** π-cation Lys36 and π-stacking His92

^a^ Those similar interactions between **6e** and sorafenib are highlighted.

**FIGURE 6:**
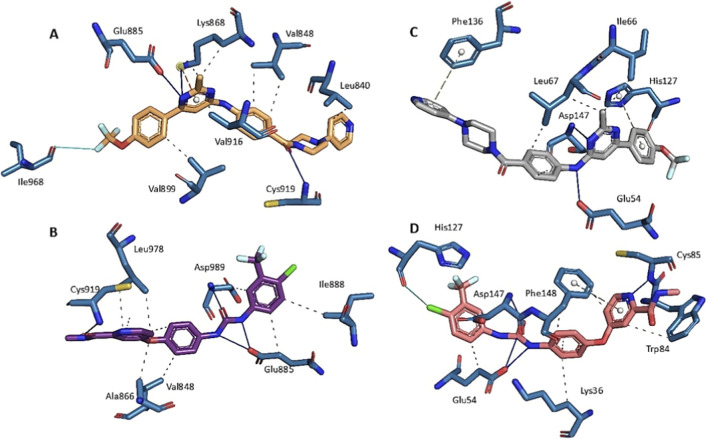
Proposed binding modes of: **(A) 6e** and **(B)** sorafenib into VEGFR-2. **(C) 6e** and **(D)** sorafenib into B-raf.

Under the applied protocol, **6e** adopts binding geometries that share several interactions with sorafenib in both VEGFR-2 and B-Raf binding sites (Table 2; Figure 6). For VEGFR-2, **6e** showed hydrophobic contacts with Val848, Ala866, Leu978, Asp989 and Phe990 and hydrogen-bonding interactions with Lys868, Glu885 and Cys919. For B-Raf, both compounds engage Leu58 and Leu67 and form hydrogen bonds consistent with the canonical binding mode (Table 2). However, docking results provide only an indication of possible binding modes, rather than definitive evidence of biochemical inhibition. Therefore, although current *in silico* data support the plausibility that **6e** can occupy the ATP binding pocket of VEGFR-2 and B-Raf, molecular dynamics simulations were performed for the VEGFR-2 receptor (the highest binding score) to evaluate the stability of the ligand-protein complexes formed by **6e** and sorafenib, and to allow a more rigorous comparison of their binding modes over time.

**FIGURE 7:**
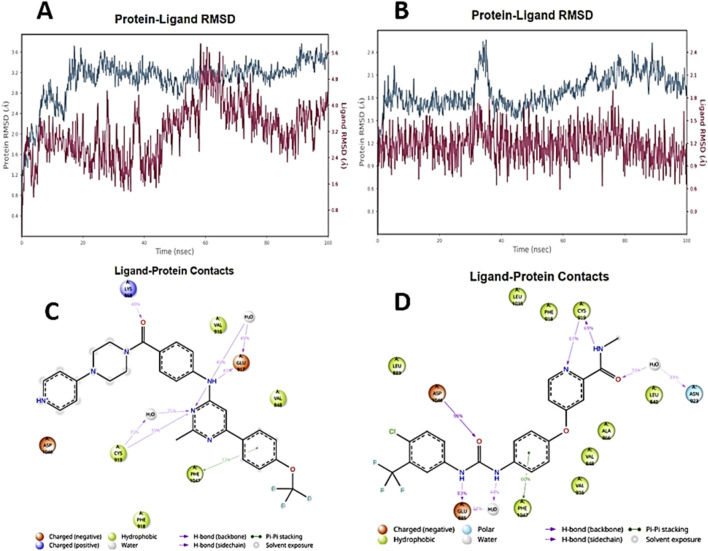
Metrics of the molecular dynamics of compound **6e** and sorafenib. RMSD graph for **6e (A)** and sorafenib **(B)**. Blue: protein RMSD; Red: Ligand RMSD. Interaction diagram highlighting persistent contacts in the complexes: **(C) 6e** and **(D)** Sorafenib.

The MD results for **6e** on VEGFR-2 showed that the protein backbone RMSD reached equilibrium after approximately 20 ns (Figure 7A). The ligand **6e** remained stable within the binding pocket, as evidenced by the ligand RMSD fitted to the protein, with the most significant fluctuation observed around 40 ns (Figure 7A). In the case of the sorafenib complex, the protein fluctuated around 35 ns and subsequently stabilised (Figure 7B); sorafenib itself maintained a consistent behaviour throughout the 100 ns simulation. Residue-based fluctuation analysis (RMSF) showed that residues in the catalytic site exhibited lower fluctuations than the surrounding loop regions, suggesting a relatively rigid binding environment during the simulation for both complexes (Supplementary Figure S1). Protein–ligand contact analysis revealed persistent interactions (>20% of the simulation time) between **6e** and Lys868, Cys919, and Glu917, mainly through hydrogen bonds, as well as sustained hydrophobic contacts with Phe1047 (Figure 7C). For the sorafenib complex, persistent hydrogen-bond interactions were observed with Asp1046, Glu885, and Cys919, and hydrophobic interactions with Phe1047 (Figure 7D). Ligand torsional profile analysis indicated that the major rotatable bonds remained in preferred conformations throughout the simulation, supporting the conformational stability of **6e** within the binding site and that of the reference ligand. Overall, these results suggest that **6e** can maintain relevant interactions within the binding pocket over a 100 ns timescale and may exhibit binding behaviour comparable to sorafenib.

### 3.5 Apoptotic cell death is induced by 6e in HepG2 cells

To study the ability of **6e** to induce apoptosis in HepG2 cells, different biological assays were used, such as flow cytometry and immunocytochemistry to determine the expression of caspase-3 and Bcl-2, Western blot experiments to detect pro- and anti-apoptotic proteins, activation of caspases-3, -7, -8, and -9, phosphorylation of p53 and ERK1/2, and determination of ROS by fluorescence.

**FIGURE 8:**
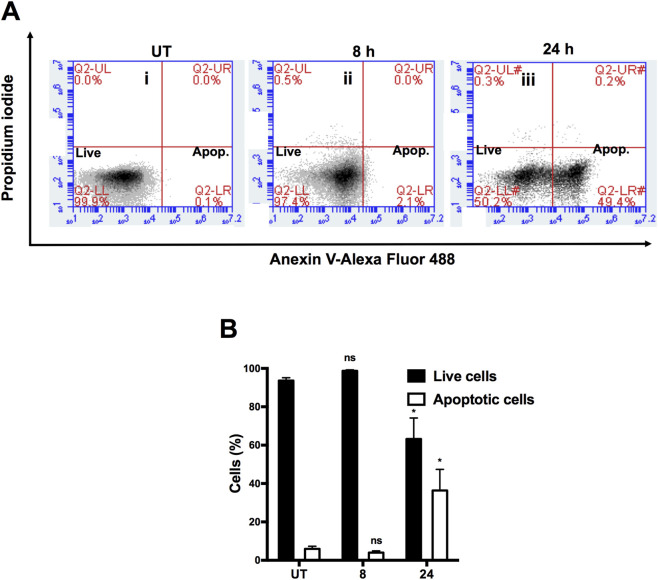
**(A) 6e** induced apoptosis in HepG2 cells as determined by flow cytometry. HepG2 cells were treated with **6e** (10 μM) for 8 or 24 h. The cells were then harvested, stained with Annexin V and PI, and analysed by flow cytometry to assess apoptosis. **(B)** Summary of apoptosis data in the form of histograms. Data are expressed as mean ± SEM from three independent experiments, each performed in triplicate. Statistical differences were assessed using one-way analysis of variance (ANOVA) followed by Dunn’s *post hoc* test. *p < 0.05 *versus* untreated (UT) group.

First, to assess **6e′s** ability to induce apoptosis, HepG2 cells were double-labelled with Annexin V-488/PI and analysed by flow cytometry. These cells were treated with 10 µM **6e** for 8 or 24 h. As shown in Figure 8A, untreated cells were predominantly Annexin-V-488 and PI-negative. This indicates that the cells were viable and did not undergo apoptosis. After treatment with **6e**, two cell populations were observed: live cells and cells undergoing apoptosis (Annexin V-488-positive and PI-negative). **6e** produced a significant increase (*p* < 0.05) in the apoptotic population and a significant decrease (*p* < 0.05) in the live cell population in HepG2 cells only after treatment for 24 h (Figure 8B).

**FIGURE 9:**
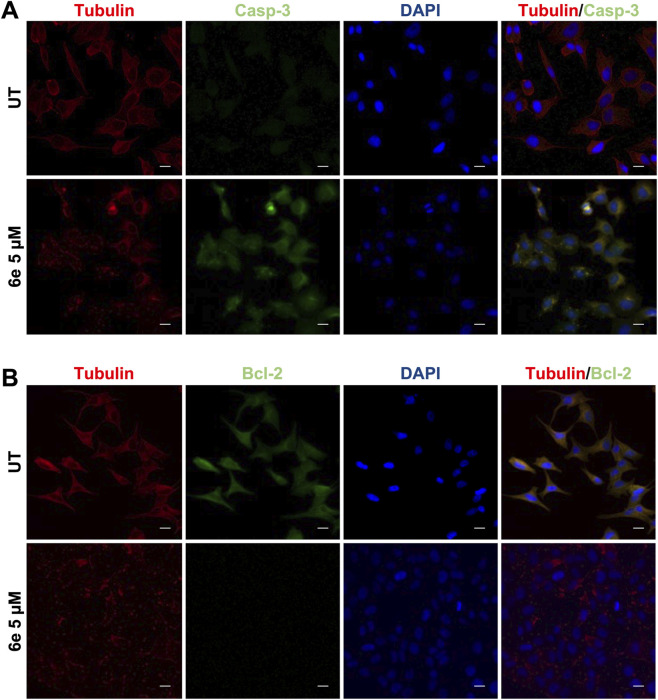
Caspase-3 and Bcl-2 protein expression in HepG2 cells. Cells were treated with 10 μM **6e** for 24 h, followed by immunocytochemical analysis, as described in Materials and Methods (section 2.3). Cells were fixed and immunostained with an anti-tubulin antibody (red), anti-caspase-3, or anti-Bcl-2 (green), and cell nuclei were counterstained with DAPI (blue). Tubulin was used as a control expression. Scale bar represents 10 μm.

We demonstrated that **6e** activated pro-apoptotic proteins and blocked anti-apoptotic proteins. HepG2 cells were immunostained for the pro-apoptotic protein caspase-3 and the anti-apoptotic protein Bcl-2. As shown in Figure 9, after 24 h of treatment with compound **6e** at 5 μM, caspase-3 protein increased (Figure 9A), whereas Bcl-2 protein was undetectable (Figure 9B), indicating that **6e** activated apoptotic pathways in HepG2 cells.

**FIGURE 10:**
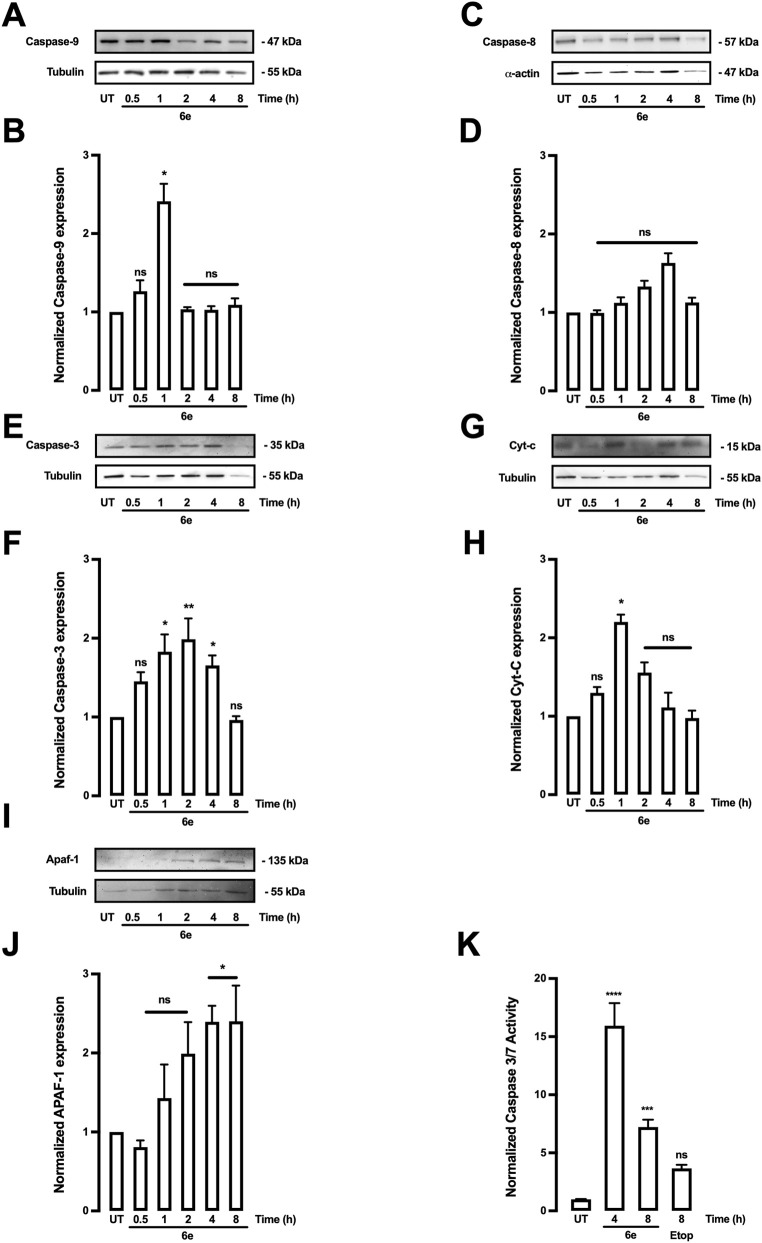
Compound **6e** induced apoptosis in HepG2 cells. **(A–K)** Cells were treated with **6e** (10 μM), and protein expression was analysed. (**A,C,E,G,I)** Representative images of Western blots used for protein detection. (**B**,**D,F,H,J)** Densitometric analyses of the experiments shown in **(A,C,E,G,I)**. Protein levels were normalised to tubulin or actin, and data are expressed relative to the untreated (UT) condition. **(K)** Caspase-Glo assay results are expressed as normalised activity relative to UT control cells. Statistical differences were assessed using one-way analysis of variance (ANOVA) followed by Dunn’s *post hoc* test. *p < 0.05; **p < 0.01, ***p < 0.001, ****p < 0.0001 vs. UT group.

To corroborate the previous results, the effect of **6e** on the expression of pro-apoptotic proteins in HepG2 cells was investigated using Western blotting and caspase activity assays. The results showed that **6e** increased levels of several apoptotic factors, including Apaf-1, caspases 3, 7, 8, and 9, and Cyt c (Figure 10). As shown in Figures 10A,C,E,G,I, specific antibodies were raised against caspases 3, 8, 9, Apaf-1, and cytochrome c, respectively, and analysed using Western blotting. As shown in Figure 10B,D,F,H,J, densitometric analysis indicated that treatment with **6e** increased the expression of the aforementioned proteins. Cyt-c and Apaf-1 were upregulated between 1 and 8 h; however, caspases-3 and -9 levels increased at 1 and 4 h after treatment with **6e** at 10 μM. Apoptosis in HepG2 cells was also confirmed by caspase-3 and -7 assays (Figure 10K). Caspase-3 and -7 activity was detected at 4 and 8 h after exposure. In addition, etoposide, a known pro-apoptotic agent, did not increase caspase-3 or -7 activity at 8 h (Figure 10K). In HepG2 cells, its activity could be extended to 18 h. Western blot analyses showed that the expression levels of caspase-8 remained unchanged (Figures 10C,D), which are upstream activators of caspases-3 and -7 in both the extrinsic and intrinsic pathways.

**FIGURE 11:**
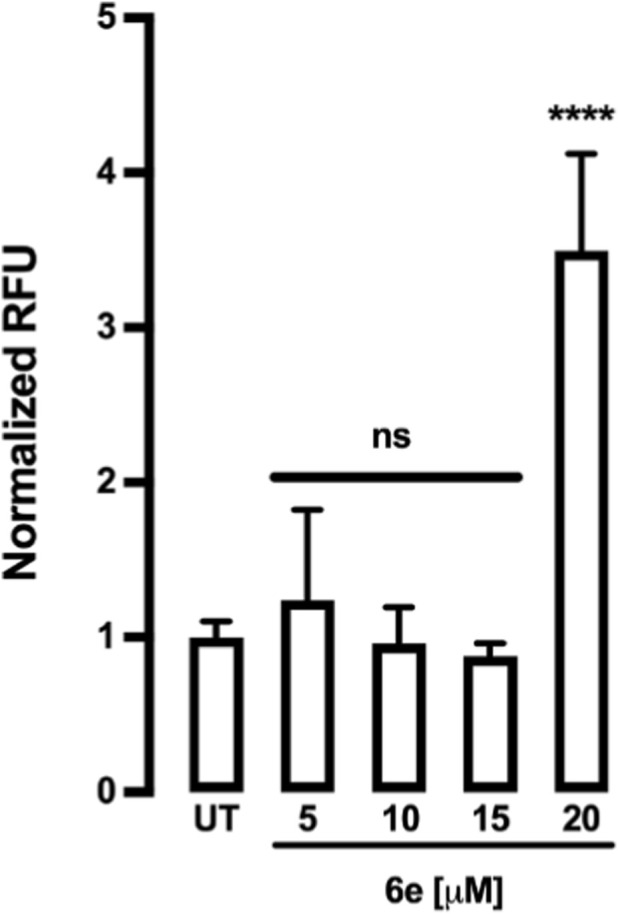
Effects of **6e** on intercellular ROS generation in HepG2 cells. The cells were exposed to **6e** at 5–20 μM for 4 h. The cells were stained with DCFDA and analysed using a fluorescence plate reader (Tecan Infinite® M200pro). Data are expressed as mean ± SEM from three independent experiments, each performed in triplicate. Statistical differences were assessed using one-way analysis of variance (ANOVA) followed by Dunn’s *post hoc* test. ****p < 0.0001 vs. untreated (UT) group. RFU = relative fluorescence units.

To investigate whether **6e** induced apoptosis by generating ROS in HepG2 cells, a fluorescence assay was performed using a DCFDA probe. As shown in Figure 11, cells treated with 20 μM **6e** showed significantly higher ROS-associated fluorescence intensity. This suggests that **6e** promoted ROS generation in HepG2 cells.

### 3.6 Phosphorylation of p53 and inhibition of ERK1/2 are induced by 6e stimuli in HepG2 cells

**FIGURE 12:**
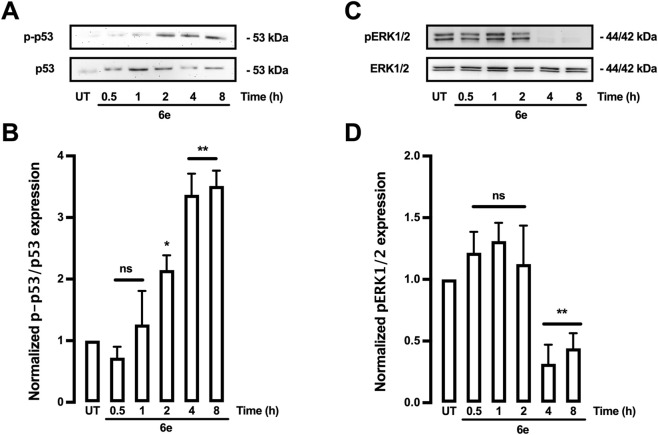
**6e** induced p53 phosphorylation and inhibited ERK1/2 phosphorylation in HepG2 cells. **(A-D)** Cells were treated with **6e** (10 μM), and protein expression was analysed. **(A**,**C)** Representative images of Western blot analysis for protein detection. **(B**,**D)** Densitometric analyses of the experiments shown in **(A**,**C)**. Protein levels were normalised to total p53 and actin, and data were expressed relative to the UT (untreated) condition. Statistical differences were assessed using one-way analysis of variance (ANOVA) followed by Dunn’s *post hoc* test. *p < 0.05 and **p < 0.01, vs. UT group.

To assess whether apoptosis was triggered via a p53-and ERK1/2-dependent pathway, the effect of **6e** on the phosphorylation of p53 and ERK1/2 in HepG2 cells was investigated. A notable increase in p-p53 (Figures 12A,B) and pERK1/2 (Figures 12C,D) proteins was detected at 2–8 and 1–2 h, respectively, following the initiation of treatment with **6e** at a concentration of 10 μM. This suggests that the apoptosis induced by **6e** was Figures 12C,D facilitated via a p53-and ERK1/2-dependent pathway, consistent with other studies on surfactin, which demonstrated that apoptosis induced by this compound was linked to ROS production and a decrease in the ERK1/2 signalling pathway (Wang et al., 2013; Zhang et al., 2021).

### 3.7 Evaluation of 6e on tumour growth *in vivo*

**FIGURE 13:**
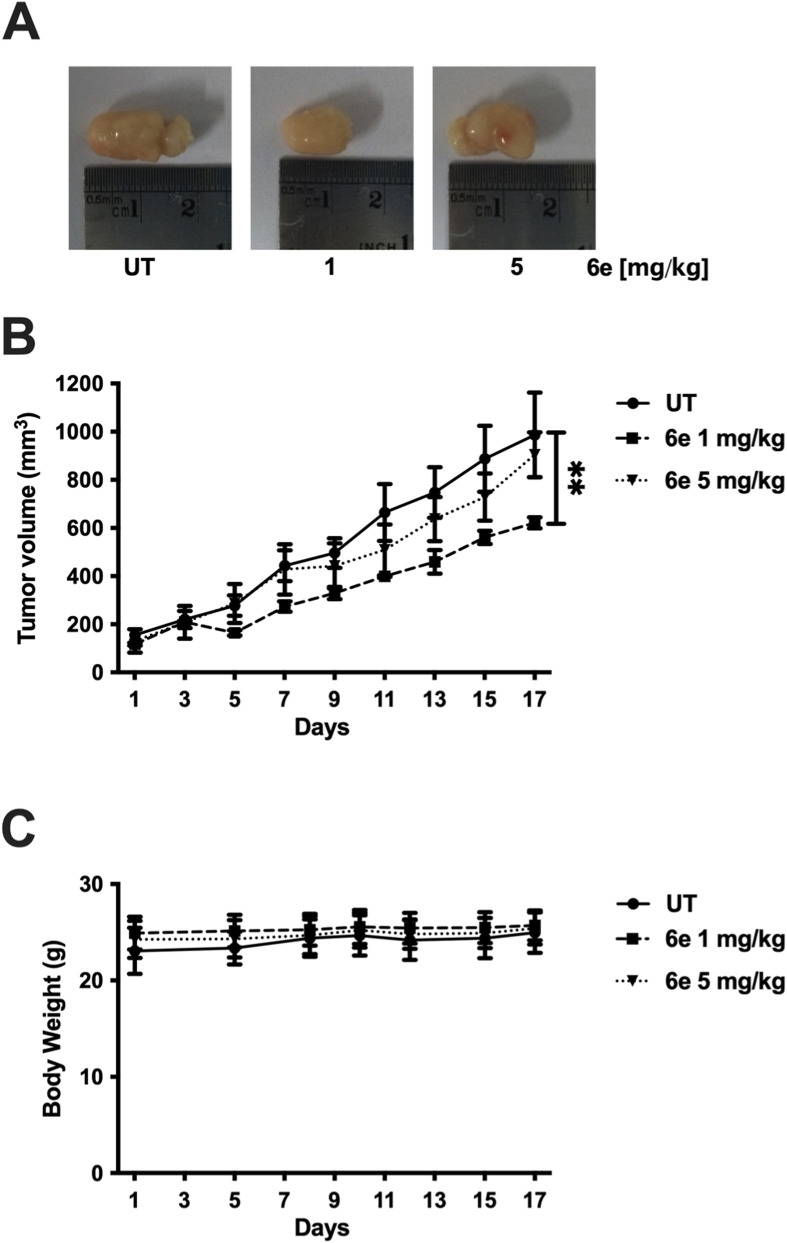
Effect of **6e** on tumour growth in a xenograft mouse model. **(A)** Representative images of tumours from each treatment group in the hepatoma cell xenograft tumour growth. **(B)** Tumour volumes of HepG2 xenografts during the 3-week treatment period for each compound and the saline control. *p < 0.05 for each compound *versus* saline control group (*n* = 6 each). **(C)** Representative body weight curves of mice bearing HepG2 xenografts during the 3-week treatment period with all four compounds. Statistical differences were assessed using one-way analysis of variance (ANOVA) followed by Dunn’s *post hoc* test. *p < 0.05 *versus* untreated (UT) group.

To evaluate the *in vivo* antitumor activity of **6e**, a HepG2 cell xenograft tumour model was used to attenuate tumour growth in mice. As described in 2.5 (Experimental section), tumour-bearing mice were treated with **6e** (1 and 5 mg/kg/mouse) or vehicle solution twice a week for 3 weeks. All tumours were harvested at the end of the study period. Representative tumours are shown in Figure 13A. Compared with the untreated (UT) group, **6e** treatment reduced tumour growth rate at 1 mg/kg throughout the study (Figure 13B). These results indicate that **6e** effectively suppressed or delayed tumorigenesis in HepG2 cells *in vivo*. Additionally, **6e** treatment did not affect the body weight of the mice (Figure 13C).

### 3.8 Calculated physicochemical properties and ADME parameters

**FIGURE 14:**
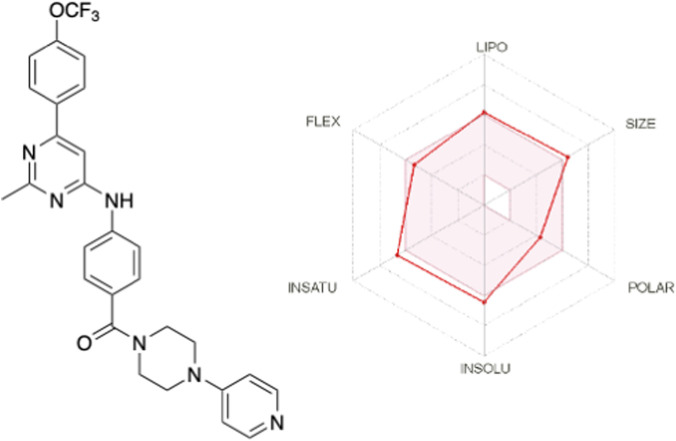
Radar plot for the bioavailability of compound **6e**. The pink area indicates the range of optimal values for each property associated with oral bioavailability. Red lines indicate the predicted properties. FLEX = flexibility, LIPO = lipophilicity, INSOLU = solubility, SIZE = size, POLAR = polarity, and SATU = saturation.

Finally, it is worth noting that both pharmacological properties and pharmacokinetic profiles are essential for drug discovery and development. Therefore, it is necessary to predict or determine the latter properties related to administration, distribution, metabolism, and excretion (ADME), and to consider them during the optimisation of a bioactive compound until it becomes a successful candidate for preclinical studies (Meanwell, 2011). The free online platform SwissADME (http://www.swissadme.ch/index.php) was used to determine the physicochemical properties of compound **6e**, in accordance with Lipinski’s rules. As shown in Table 3, **6e** meets the criteria for good permeability and bioavailability based on the hydrogen-bond donor (HBD), hydrogen-bond acceptor (HBA), and cLogP values (Lipinski et al., 2001). Still, the molecular weight (MW) of **6e** is closer to the optimal value. Furthermore, according to Veber’s rules, **6e** has a topological polar surface area (TPSA) and several rotatable bonds (NRB) of <140 Å^2^ and ≤10 NRB (Table 3) (Veber et al., 2002). These values indicate that **6e** has a high ability to penetrate cell membranes and good oral absorption, according to the Lipinski and Veber rules. In addition, the SwissADME platform provides a bioavailability radar plot that considers the following parameters: flexibility (FLEX), lipophilicity (LIPO), solubility (INSOLU), size (SIZE), polarity (POLAR), and saturation (INSATU), and if all parameters are within the desired range (pink region), good oral absorption is expected for this compound. Figure 14 shows that almost all these criteria were met for **6e**.

**TABLE 3:** Molecular properties of compound **6e**.

Compound	MW (Da)	HBA	HBD	cLogP	TPSA (Å^2^)	NRB
Desirable value	≤500	≤10	≤5	≤5	≤140	≤10
**6e**	534.53	8	1	4.35	83.48	8

MW, molecular weight; HBA, number of hydrogen bond acceptors; HBD, number of hydrogen bond donors; cLogP, consensus LogP value; TPSA, topological polar surface area; NRB, number of rotatable bonds. All the values were predicted using SwissADME (http://www.swissadme.ch/index.php).

## 4 Discussion

In this study, novel 2,4,6-trisubstituted pyrimidine derivatives were synthesised and evaluated for their potential as antitumour agents against HCC. As noted in the introduction, pyrimidine is a crucial heterocyclic compound in medicinal chemistry owing to its diverse biological and pharmacological functions. Pyrimidine is present in natural substances such as nucleotides, nucleic acids, and purines, among others (Mahapatra et al., 2021). It exhibits high synthetic versatility, enabling the formation of several derivatives by substitution at the 2-, 4-, or 6-positions, as well as at the nitrogen atoms. Each of these derivatives has interesting biological properties (Basha and Goudgaon, 2021). This makes pyrimidine a building block for many anticancer drugs (Mahapatra et al., 2021), including gemcitabine, 5-fluorouracil, and floxuridine (Albratty and Alhazmi, 2022).

In our study, a four-step synthetic process yielded 12 compounds, two of which were promoted by MW irradiation. This methodology is a valuable and direct approach for the synthesis of required compounds.

After testing these compounds against HepG2 cells, compound **6e** showed the best antiproliferative effect among the pyrimidine derivatives studied. In addition, **6e** demonstrated cytotoxicity in two HCC cell lines with some specificity, as it showed reduced efficacy in other cancer cell lines and in non-transformed Vero cells. Compound **6e** showed an IC_50_ range of 5.6–11.6 µM in HCC cell lines. It is important to note that a potential antitumour drug must demonstrate low toxicity in mammalian host cells. Therefore, these more selective compounds are highly promising for the development of new antitumour agents. These results are consistent with the National Cancer Institute (NCI) protocols, which consider compounds with IC_50_ values < 10 μM or 15 μM to be active (Shoemaker, 2006). It is also worth noting that when evaluating potential antitumour drugs, it is crucial to prioritise compounds that show low toxicity in non-tumour mammalian cells. A compound that meets these selectivity requirements shows promise for the development of new and effective antitumor agents, and compound **6e** could fall into this category. Nonetheless, it is difficult to establish a chemical relationship between these compounds, as only **6e** showed activity against cancer cell lines. However, it appears that the presence of a pyridine ring linked to the piperazine fragment is crucial for the cytotoxicity of these pyrimidine derivatives. Therefore, **6e** demonstrated potential for specificity and cytotoxicity in HCC cell lines, making it a promising candidate for therapeutic applications.

Given sorafenib’s pharmacological effects on HCC, we investigated whether **6e** targets the same kinases as sorafenib *in silico*. Docking studies indicated that **6e** exhibits binding affinities comparable to those of sorafenib for two well-known targets, VEGFR-2 and B-Raf. Likewise, the interaction patterns at the respective binding sites involved similar amino acids via hydrophobic interactions and hydrogen bonds. Molecular dynamics studies have, over time, corroborated the proposed binding mode of **6e** at the ATP site of VEGFR-2. Therefore, it is possible to hypothesise that **6e** may be a multikinase inhibitor, and that this behaviour is related to its cytotoxicity in HCC cells and its ability to induce apoptosis. Specifically, inhibition of VEGFR-2 in cancer cells has been widely reported (Abdallah et al., 2022; Farzaneh Behelgardi et al., 2022).

As mentioned above, it is essential to develop chemical agents that can induce apoptosis in cancer cells (Kist and Vucic, 2021). In this regard, compound **6e** induced cell death in HepG2 cells, as evidenced by increased annexin-V-positive cells at 10 µM after 24 h of treatment. Moreover, the induction of apoptosis appears to stem from a disruption in the balance between antiapoptotic and proapoptotic factors. This is evidenced by a reduction in Bcl-2 expression, along with an increase in caspase activity, as well as heightened expression of Apaf-1 and p53, and an elevated release of Cyt-c into the cytoplasm (Singh et al., 2022).

ROS, the primary molecules generated by oxidative stress, have been identified in various tumour cells and are considered significant contributors to tumour initiation, progression, and recurrence. Previous studies have reported that ROS can modulate the ERK, nuclear factor kappa B, PI3K/Akt, and VEGFR-2 pathways, thereby influencing cell proliferation, apoptosis, and metastasis (Sosa et al., 2013). However, excessive ROS levels are cytotoxic, leading to cell death (Moloney and Cotter, 2018). The present study demonstrated that ROS levels increase rapidly following **6e** stimulation, subsequently activating ERK and p53. Based on its apoptosis-related effects, compound **6e** is likely to activate the intrinsic mitochondrial apoptotic pathway. Under exogenous challenge, the apoptotic program involves the inhibition of anti-apoptotic factors (e.g., Bcl-2), followed by mitochondrial membrane permeabilisation, which releases Cyt-c into the cytosol. This is followed by the formation of apoptosomes (Cyt-c, Apaf-1, and caspase-9 complexes), which activate pro-caspases (caspase-3 and -7) (Hussar, 2022). In turn, p53 and ERK1/2, through the p53-upregulated modulator of apoptosis (PUMA), transcriptionally regulate BH3 proteins that inhibit the anti-apoptotic Bcl-2 family member. The intrinsic pathway involves, among other things, DNA damage or endoplasmic reticulum stress. Whether the apoptotic activity of **6e** is responsible for these deleterious cellular effects remains to be investigated. In addition, compound **6e** inhibited HepG2 xenograft tumorigenesis *in vivo* at a dose of 1 mg/kg with no evidence of toxicity in the animals.

## 5 Conclusion

In conclusion, given that compounds based on a pyrimidine scaffold have demonstrated antitumor properties, we designed and synthesised 12 trisubstituted pyrimidines in this study. One of these, pyrimidine derivative **6e**, exhibited *in vitro* cytotoxicity against two HCC cell lines, but low cytotoxicity in Vero cells. Compound **6e** appears to trigger the intrinsic mitochondrial apoptotic pathway and may inhibit proteins involved in cancer cell survival, proliferation, and tumorigenesis. A search for biological targets involved in the antiproliferative effect using *in silico* methodologies would indicate that **6e** could bind to two kinases, VEGF-2 and B-raf, with a similar affinity as sorafenib. In addition, **6e** reduced the *in vivo* growth of HepG2 cell tumours in an animal model at a dose of 1 mg/kg and improved mouse survival. Therefore, it would be worthwhile to explore the potential of **6e**, including its pharmacological targets, not only as a lead for structural modifications but also as a basis for the search for new agents against HCC.

## Data Availability

The original contributions presented in the study are included in the article/Supplementary Material, further inquiries can be directed to the corresponding authors.
