# Supplementary material for: New pyrimidine derivatives as potential agents against hepatocellular carcinoma: design, synthesis, and in vitro and in vivo biological evaluations
Source: Front Pharmacol. 2026 Jan 30;17:1745214. doi: 10.3389/fphar.2026.1745214 (PMC12901390; doi:10.3389/fphar.2026.1745214)
Supplement: Supplementary file 1 [file DataSheet1.docx]

Supplementary materials

New pyrimidine derivatives as potential agents against hepatocellular carcinoma: Design, synthesis, and *in vitro* and *in vivo* biological evaluations

Jeanluc Bertrand^1^, Ignacio Montorfano^2^, Ramón Pérez-Castro^3,4^, Ricardo Valdés-Valdés^3^, Jacqueline Romero^4^, Thalía Delgado^1^, Iván Brito^5^, Juan F. Santibáñez^6^, Alan R. Cabrera^7^, María Paola Vieytes^2^, Javier Echeverría^8*^, Cristian O. Salas^1*^, César Echeverría^2,9,10*^

^1^ Departamento de Química Orgánica, Facultad de Química y de Farmacia, Pontificia Universidad Católica de Chile, Santiago, Chile.

^2^ ATACAMA-OMICS, Laboratorio de Biología Molecular y Genómica, Facultad de Medicina, Universidad de Atacama, Copiapó, Chile.

^3^ *In vivo* Tumor Biology Research Facility, Centro Oncológico, Universidad Católica del Maule, Talca, Chile.

^4^ Laboratorio de Investigaciones Biomédicas, Facultad de Medicina, Universidad Católica del Maule, Talca, Chile.

^5^ Departamento de Química, Facultad de Ciencias Básicas, Universidad de Antofagasta, Antofagasta, Chile.

^6^ Group for Molecular Oncology, University of Belgrade, Institute for Medical Research, National Institute of Republic of Serbia, Belgrade, Serbia.

^7^ Departamento de Química Inorgánica, Facultad de Química y de Farmacia, Pontificia Universidad Católica de Chile, Santiago, Chile.

^8^ Departamento de Ciencias del Ambiente, Facultad de Química y Biología, Universidad de Santiago de Chile, Santiago, Chile.

^9^ Instituto de Ciencias Naturales, Facultad de Medicina Veterinaria y Agronomía, Universidad de Las Américas, Santiago, Providencia, Chile.

^10^ Centro de Investigación en Ciencias Biológicas y Químicas, Universidad de Las Américas, Santiago, Chile.

**Corresponding authors:** Javier Echeverría, e-mail: javier.echeverriam@usach.cl; Cristian Salas, e-mail: cosalas@uc.cl; César Echeverría, e-mail: cecheverria@udla.cl.

**Index**

^1^H, ^13^C and ^19^F NMR of selected compounds…………………………………………………………pag 2-29

Mass spectra of final compounds……………………………………………………………….…….pag 30-35

HPLC of final compounds.….………………………………………………………………………...pag 36-47

**Table S1**. Selected crystallographic data of compound **6h**…..………………………….……………….pag 48

**Table S2.** Primary and secondary antibodies used in western blot experiments…………….…………..pag 49

**Table S3.** Primary and secondary antibodies used in immunocytochemistry……………….…………..pag 49

**Table S4.** *In vitro* cytotoxicity of compounds **6a-l** on cancer cell lines…………………………………pag 50

**Table S5.** Docking protocol validation for VEGFR-2………………………..…..……...……………pag 51

**Table S5.** Docking protocol validation for B-raf……………………………..…..……...……………pag 52

**Figure S1.** Superposition of the protein-ligand total contacts for **6e** (**A**), and sorafenib (**B**).…...………pag 53

^1^H NMR spectra of compound **3**


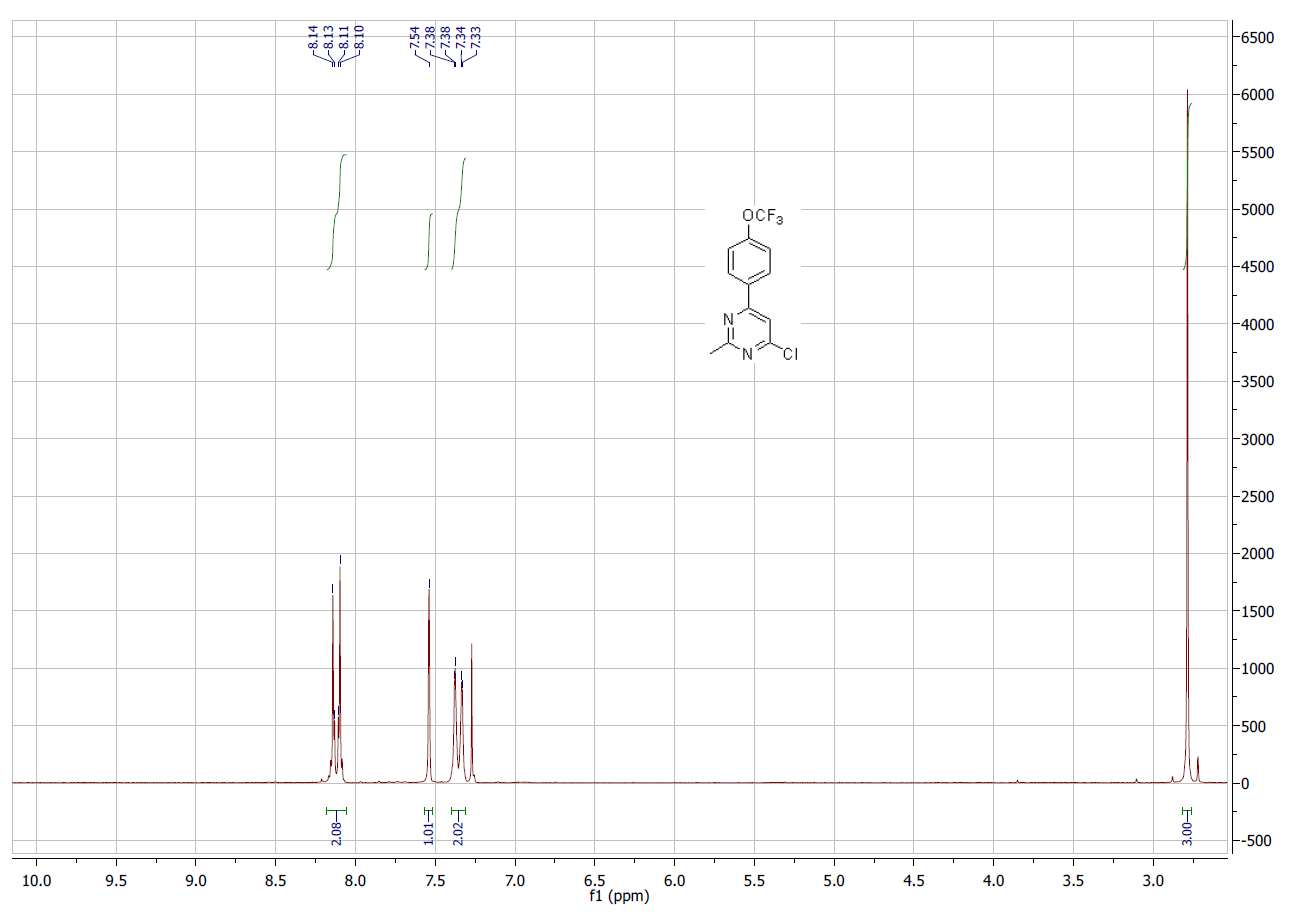


^13^C NMR spectra of compound **3**


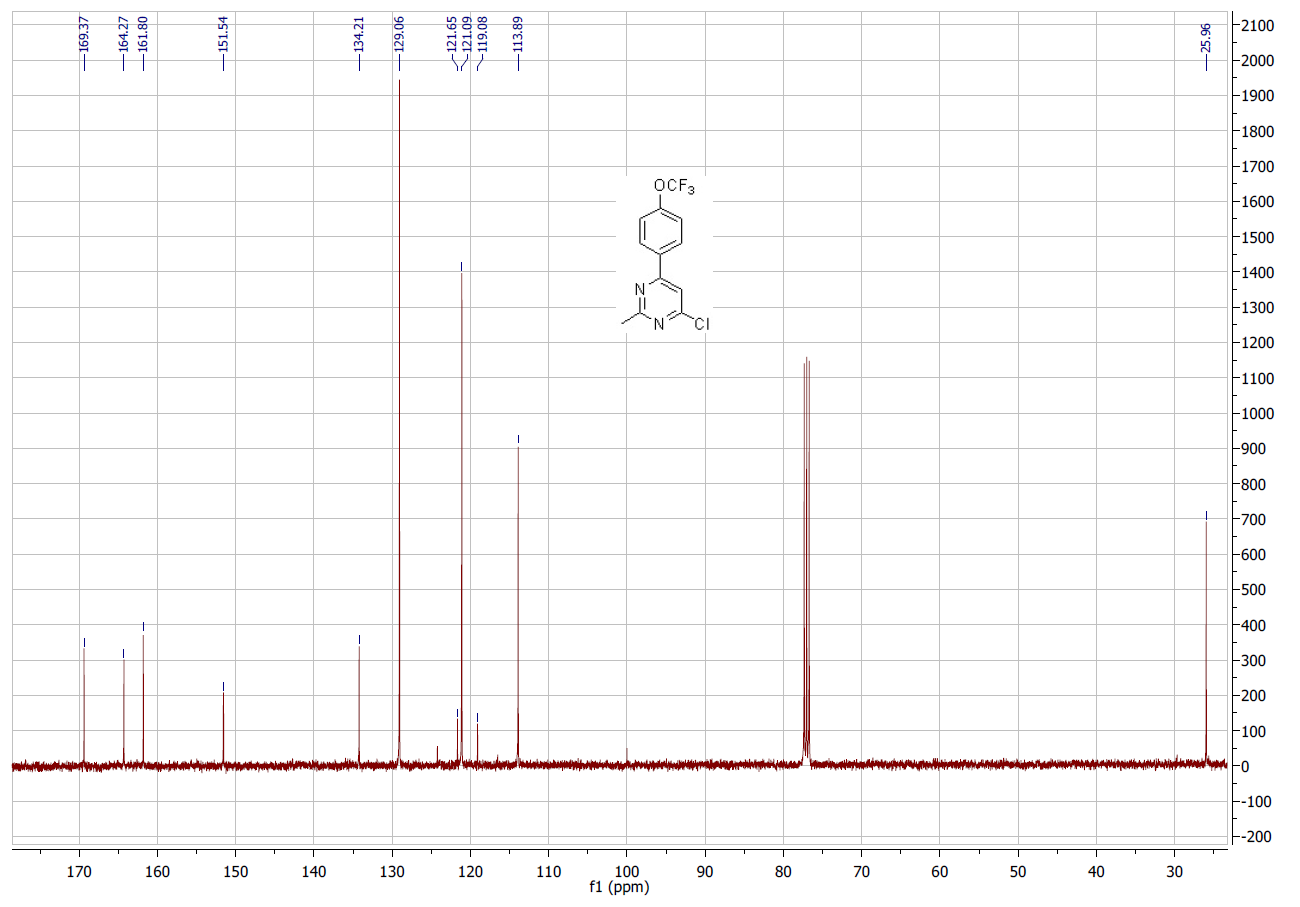


^19^F NMR spectra of compound **3**


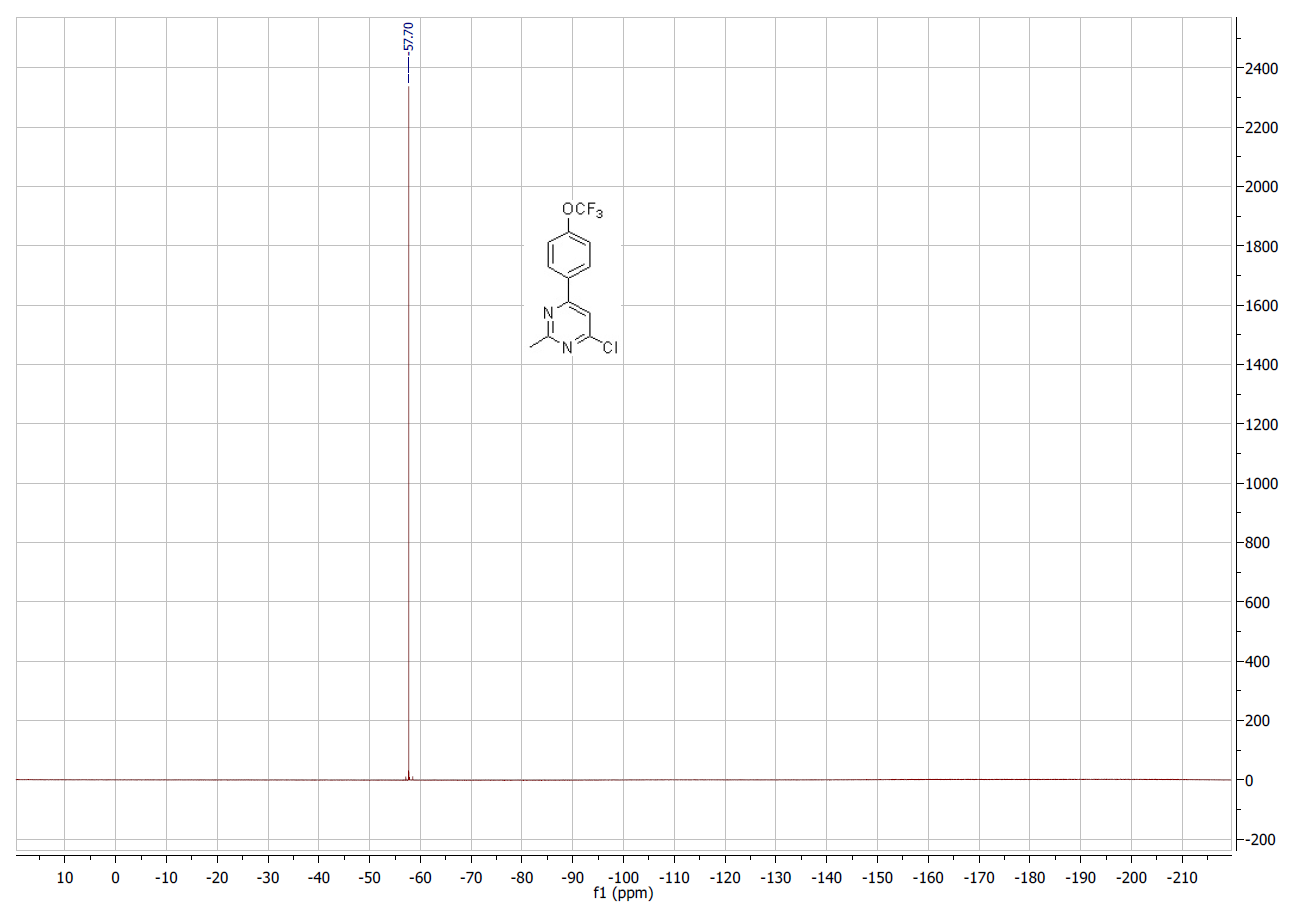


^1^H NMR spectra of compound **4**


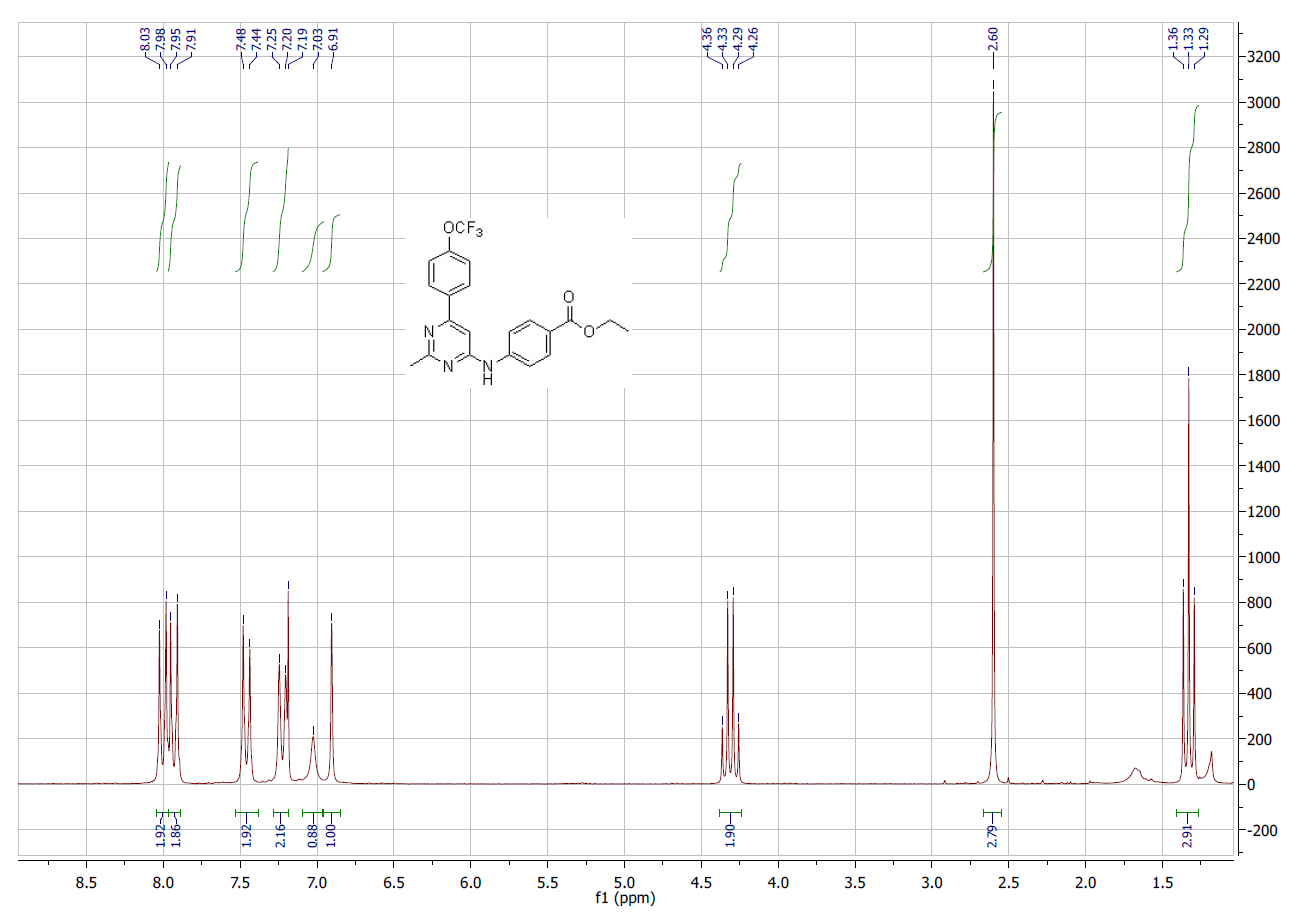


^13^C NMR spectra of compound **4**


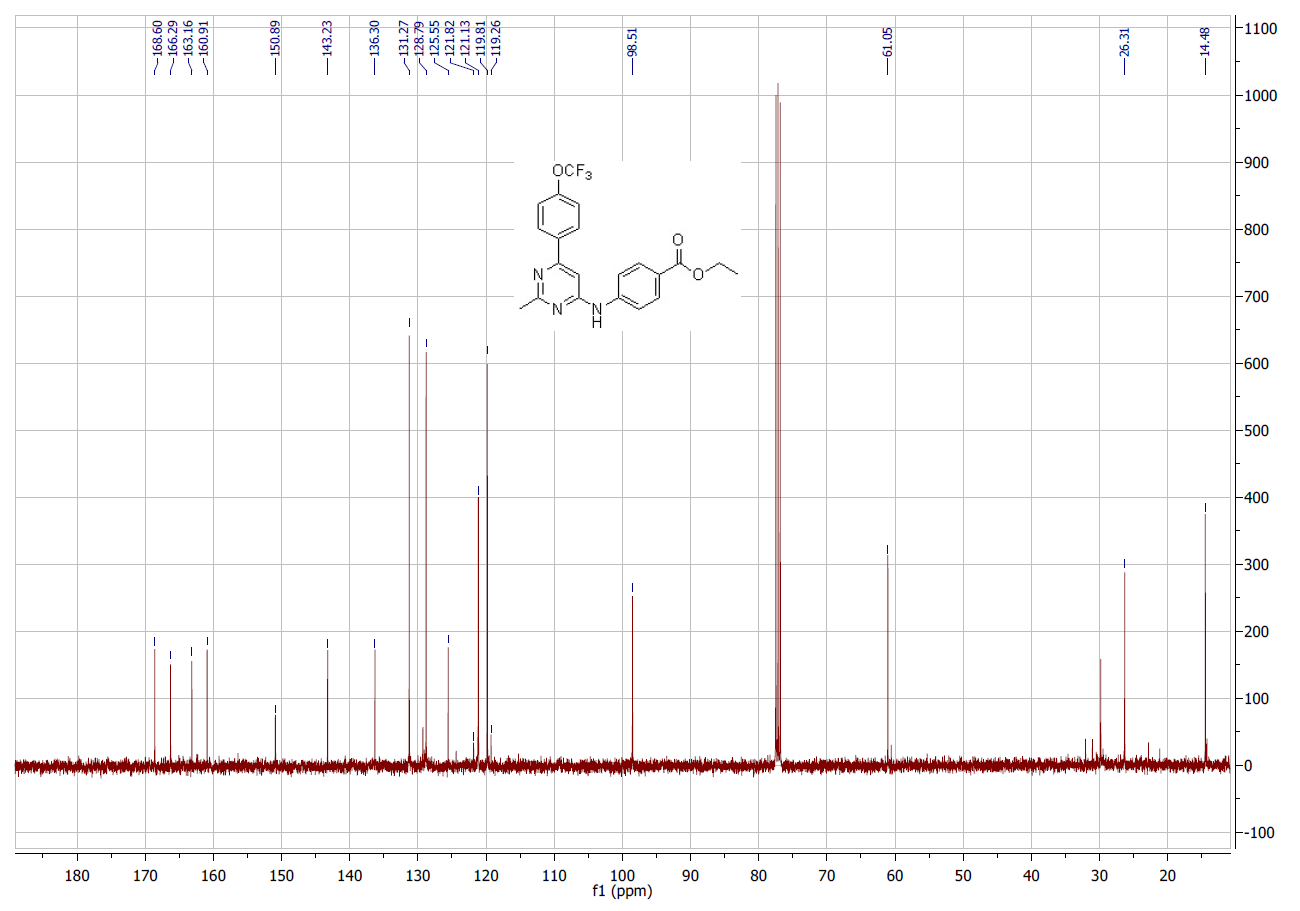


^19^F NMR spectra of compound **4**


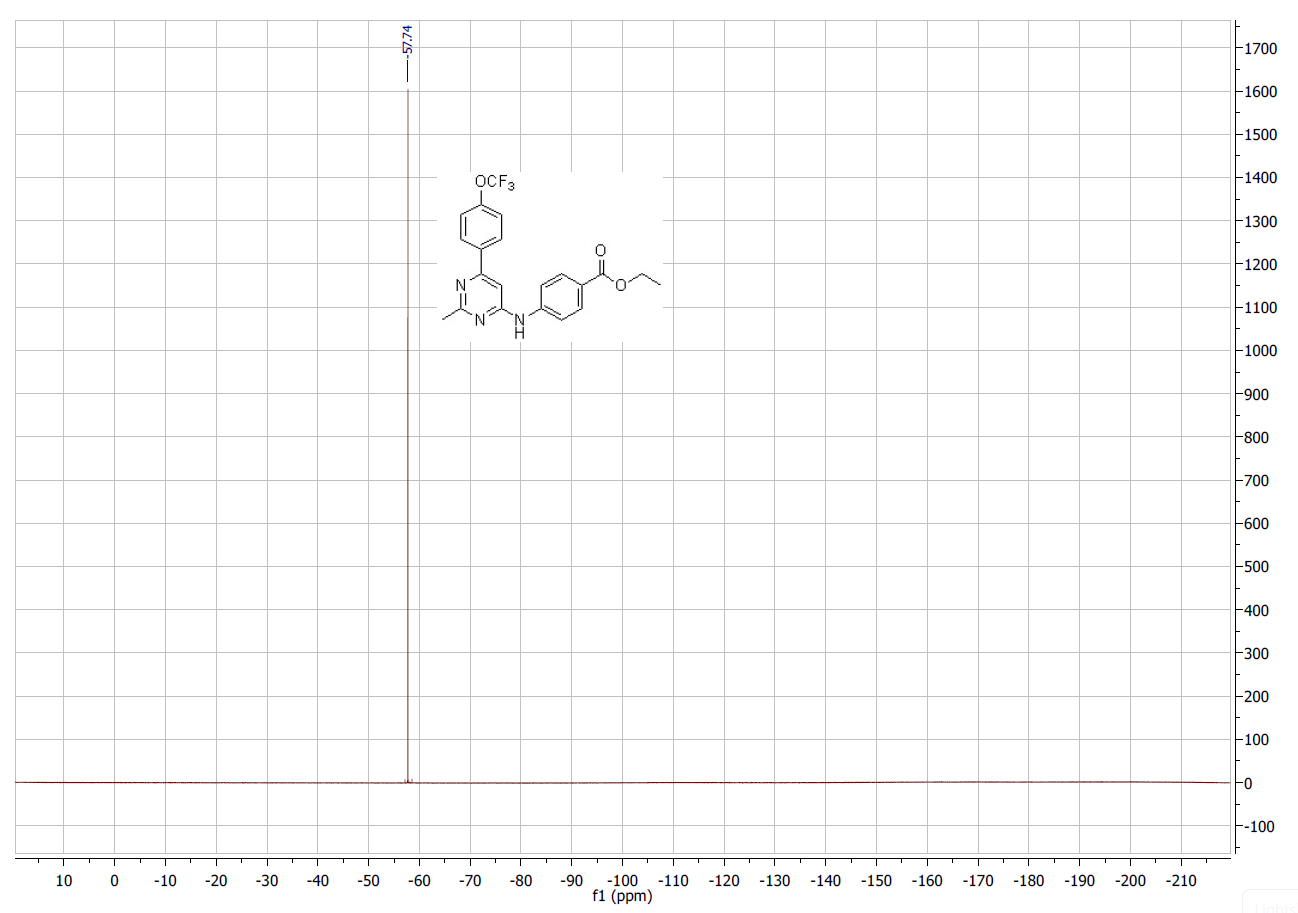


^1^H NMR spectra of compound **6a**


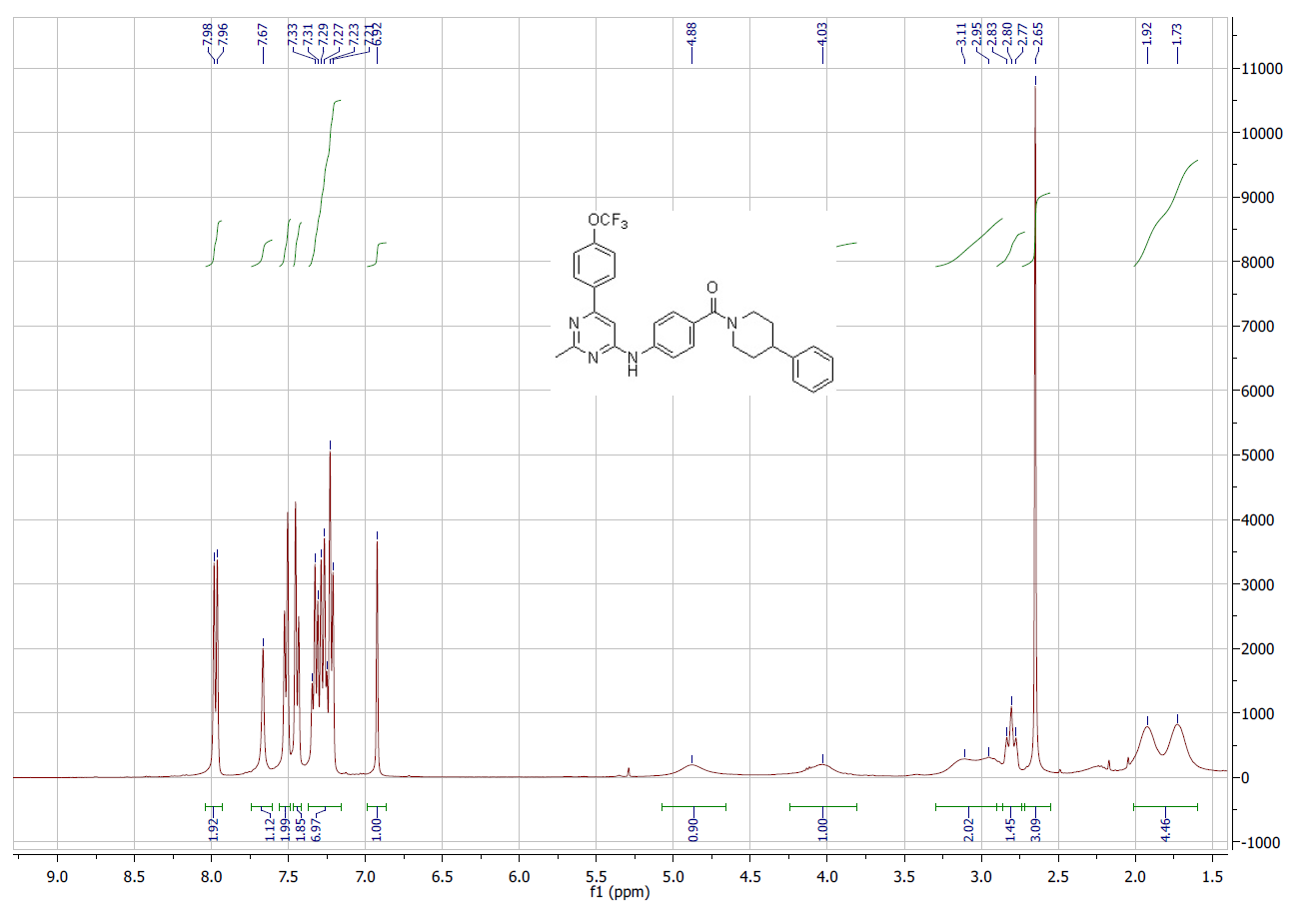


^13^C NMR spectra of compound **6a**


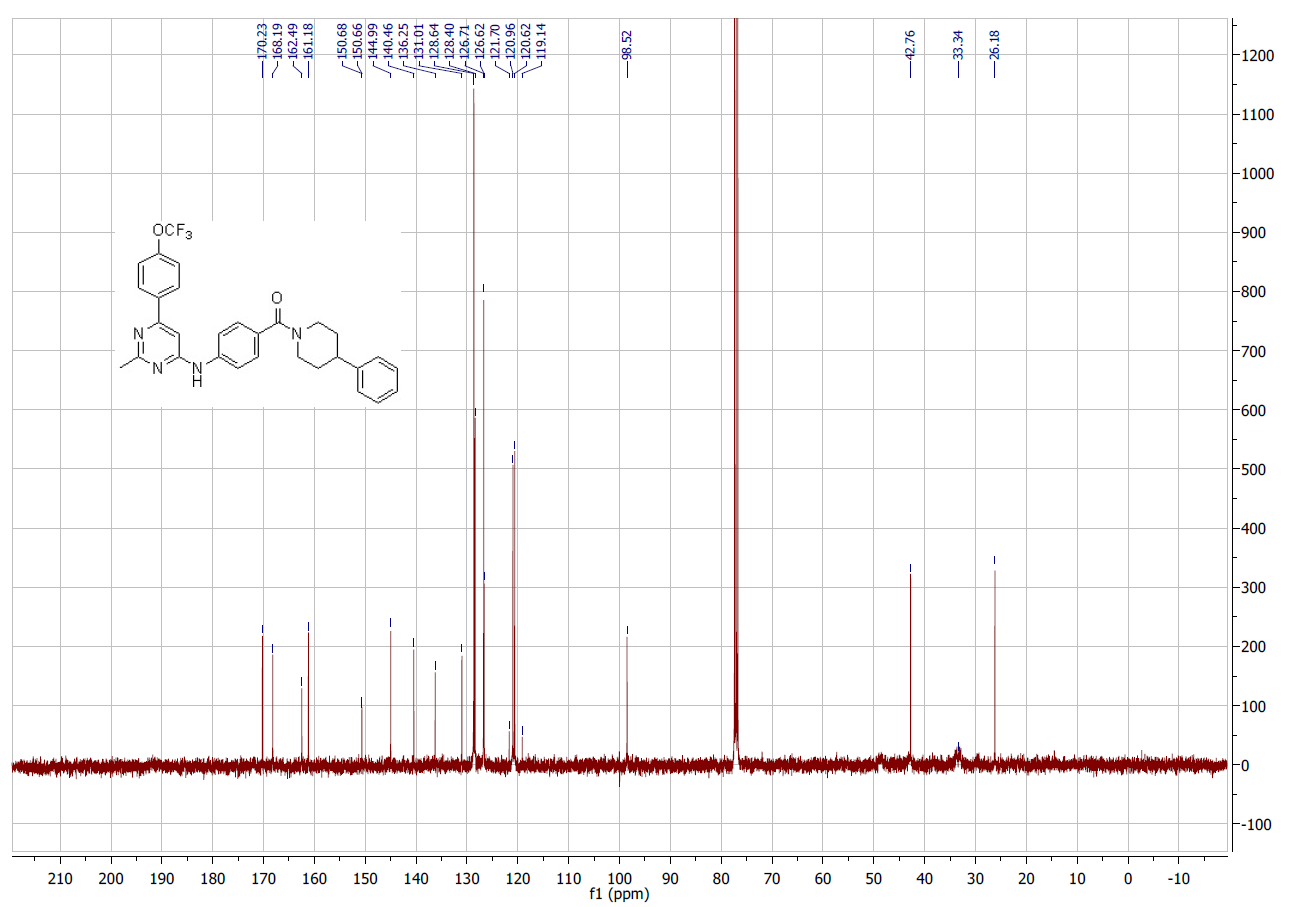


^19^F NMR spectra of compound **6a**


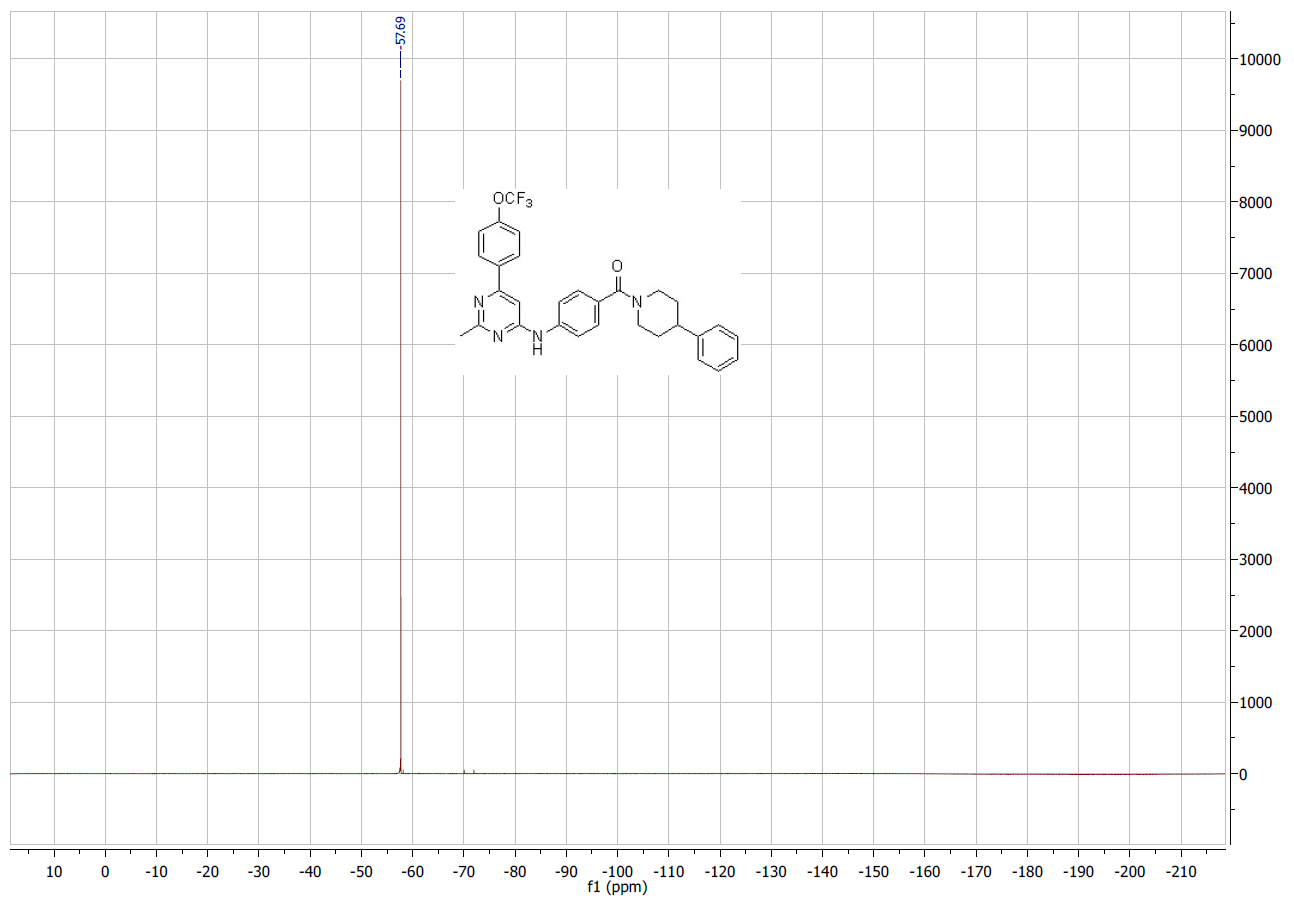


^1^H NMR spectra of compound **6b**


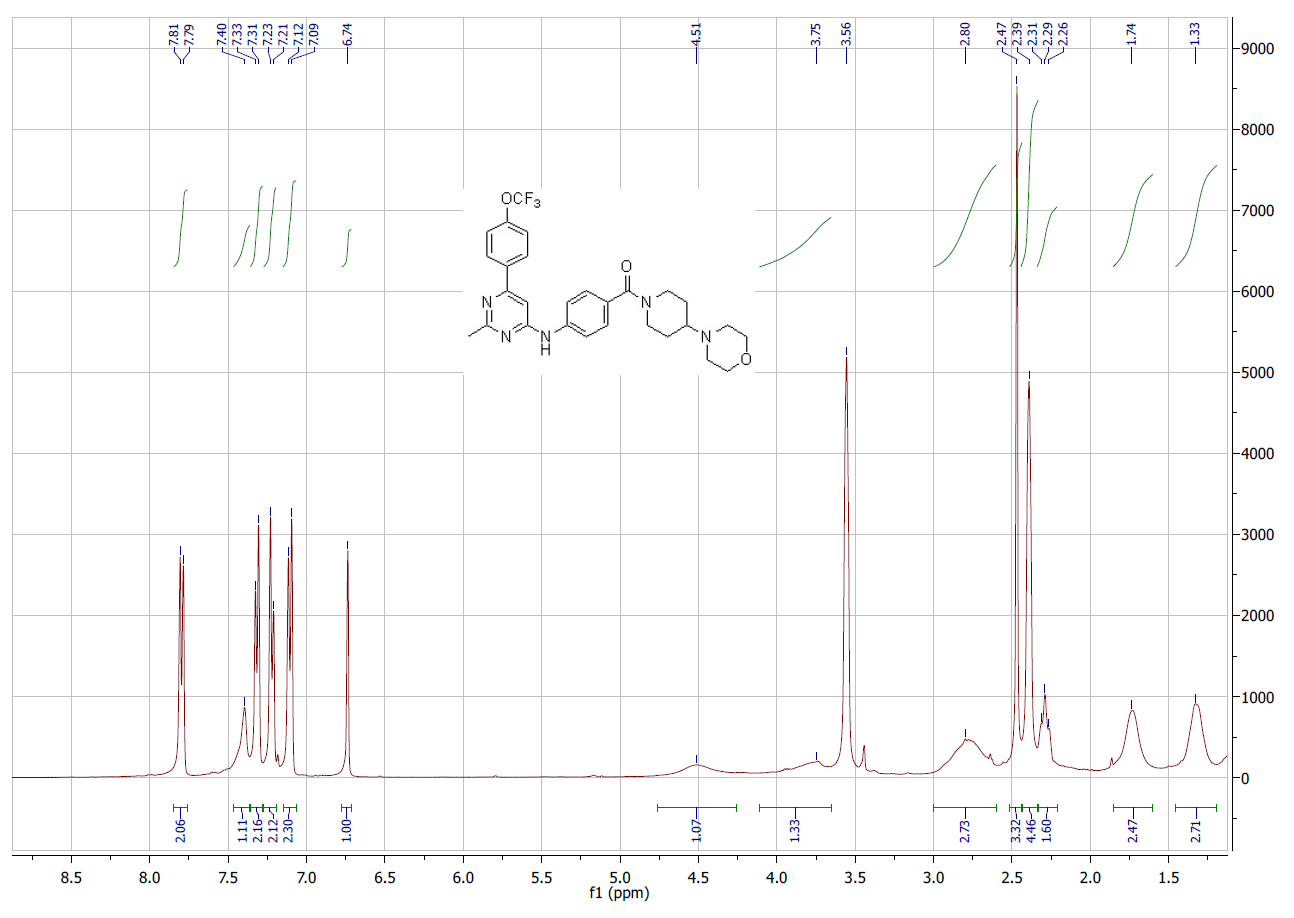


^13^C NMR spectra of compound **6b**


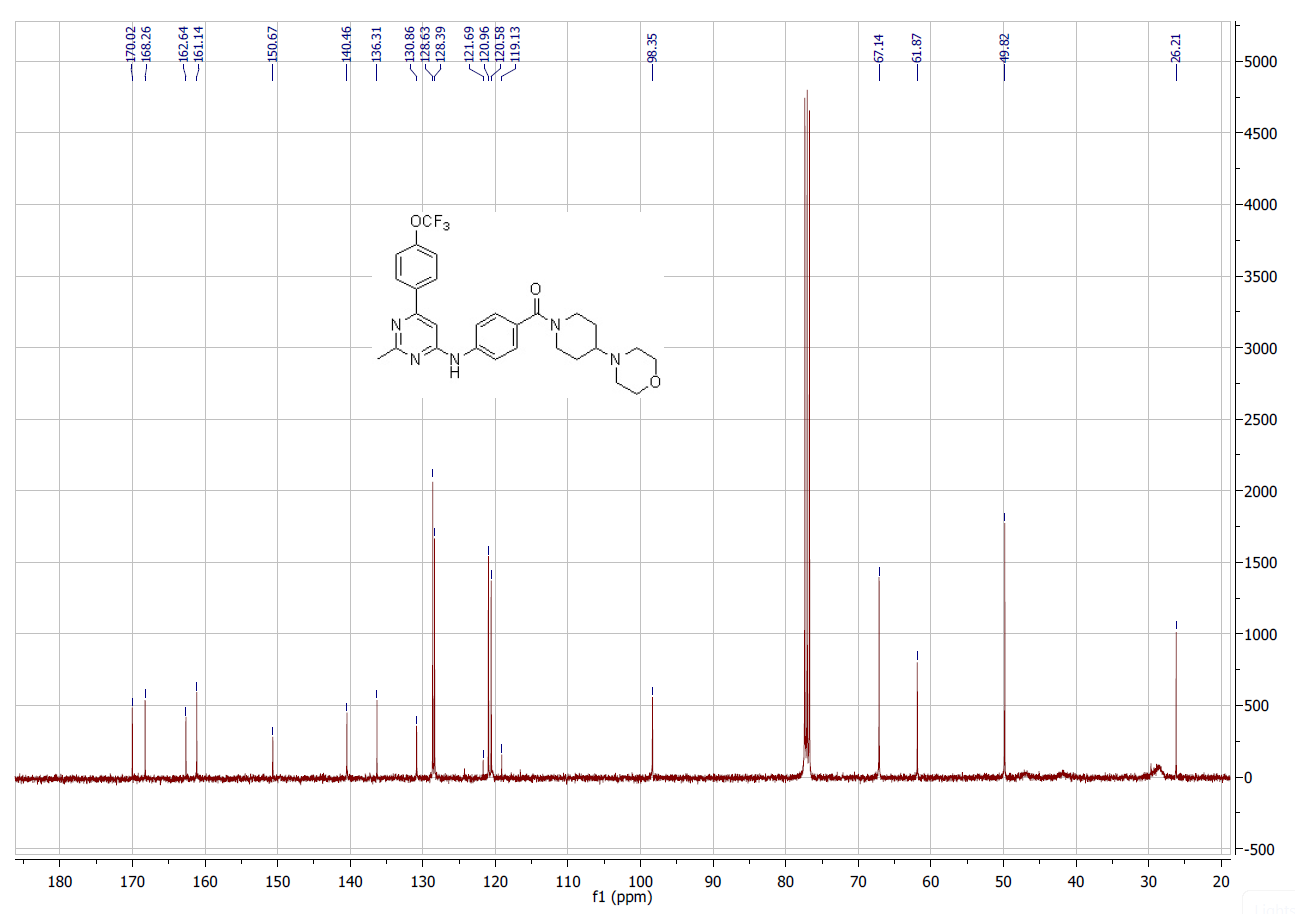


^19^F NMR spectra of compound **6b**


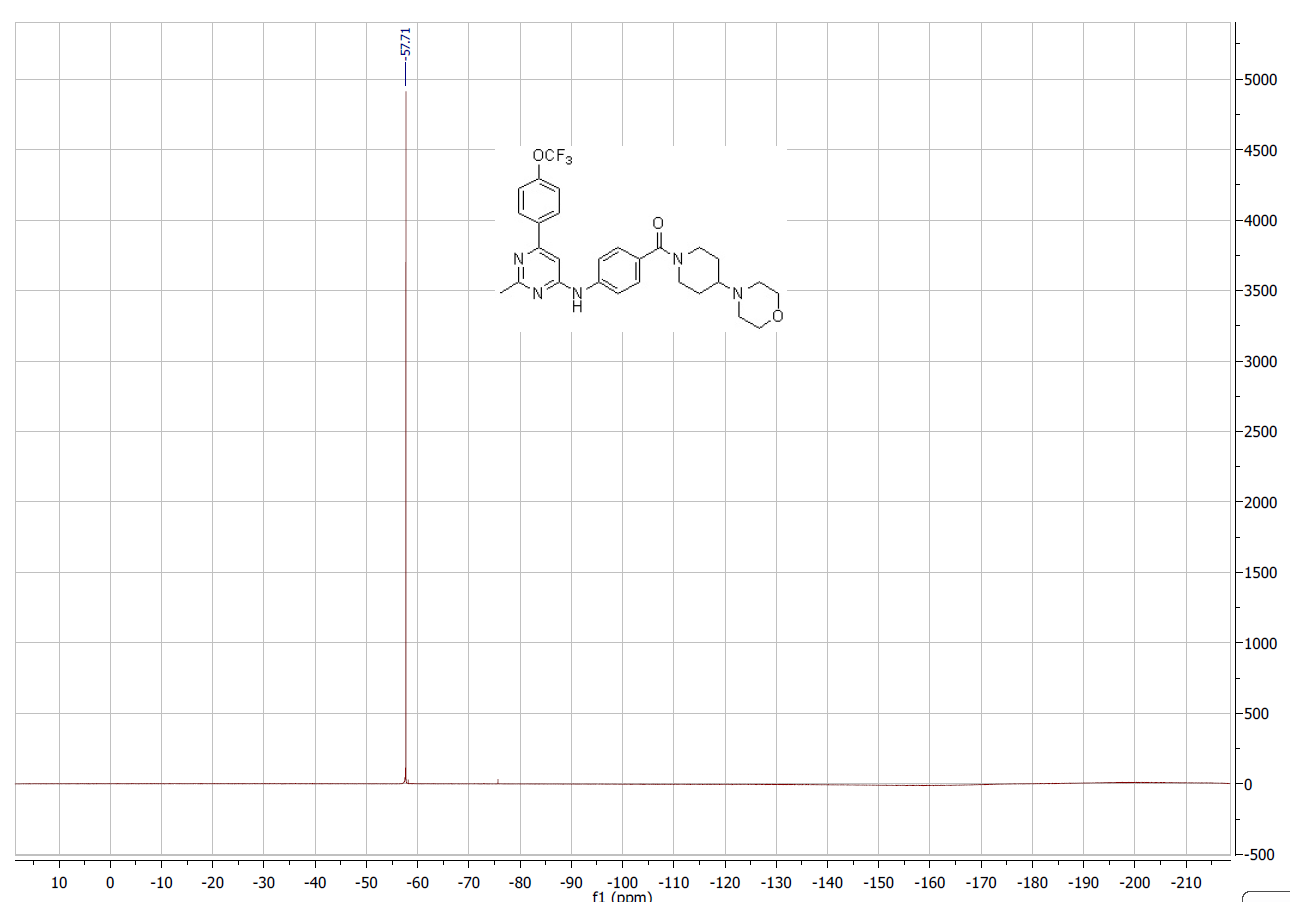


^1^H NMR spectra of compound **6c**

^
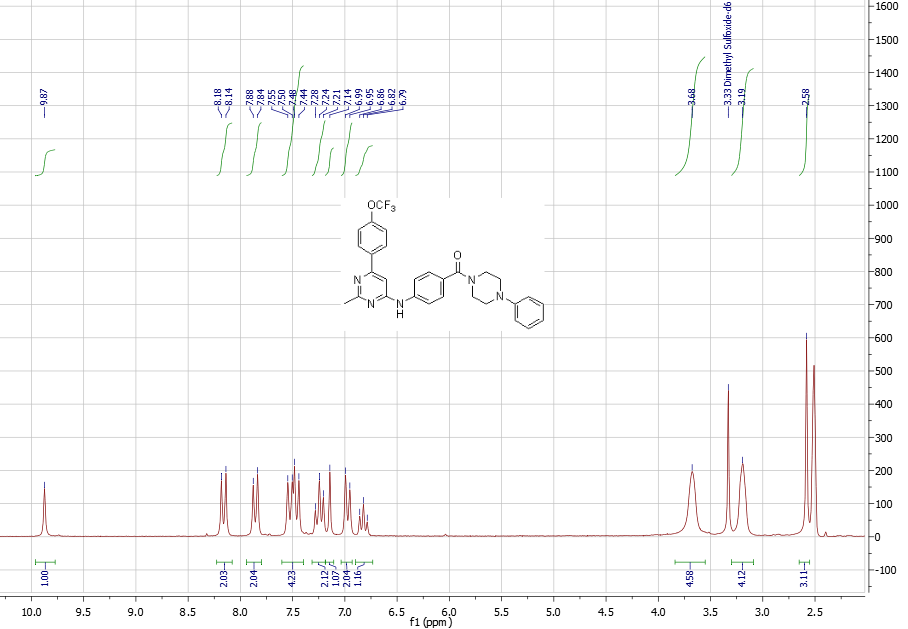
^

^13^C NMR spectra of compound **6c**


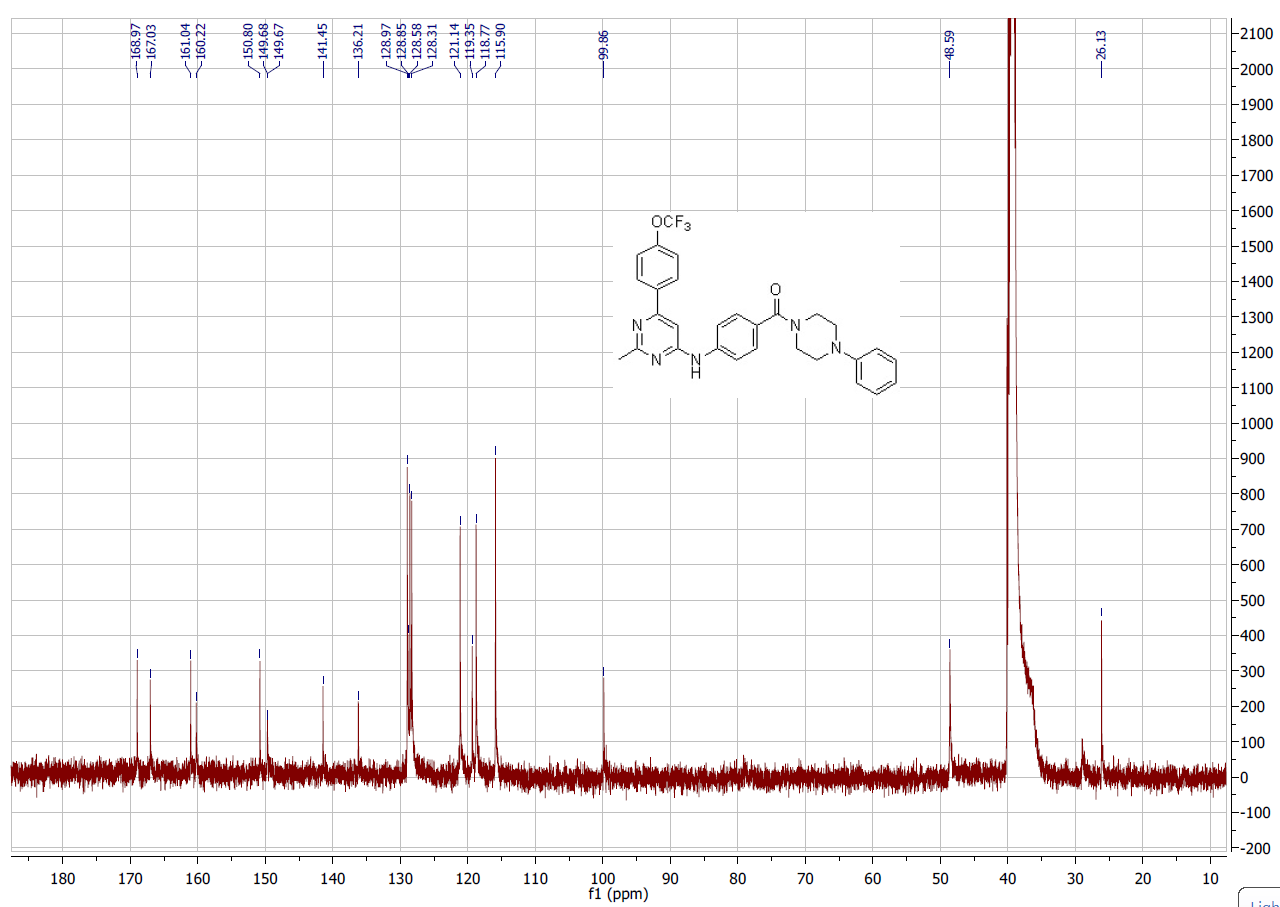


^19^F NMR spectra of compound **6c**


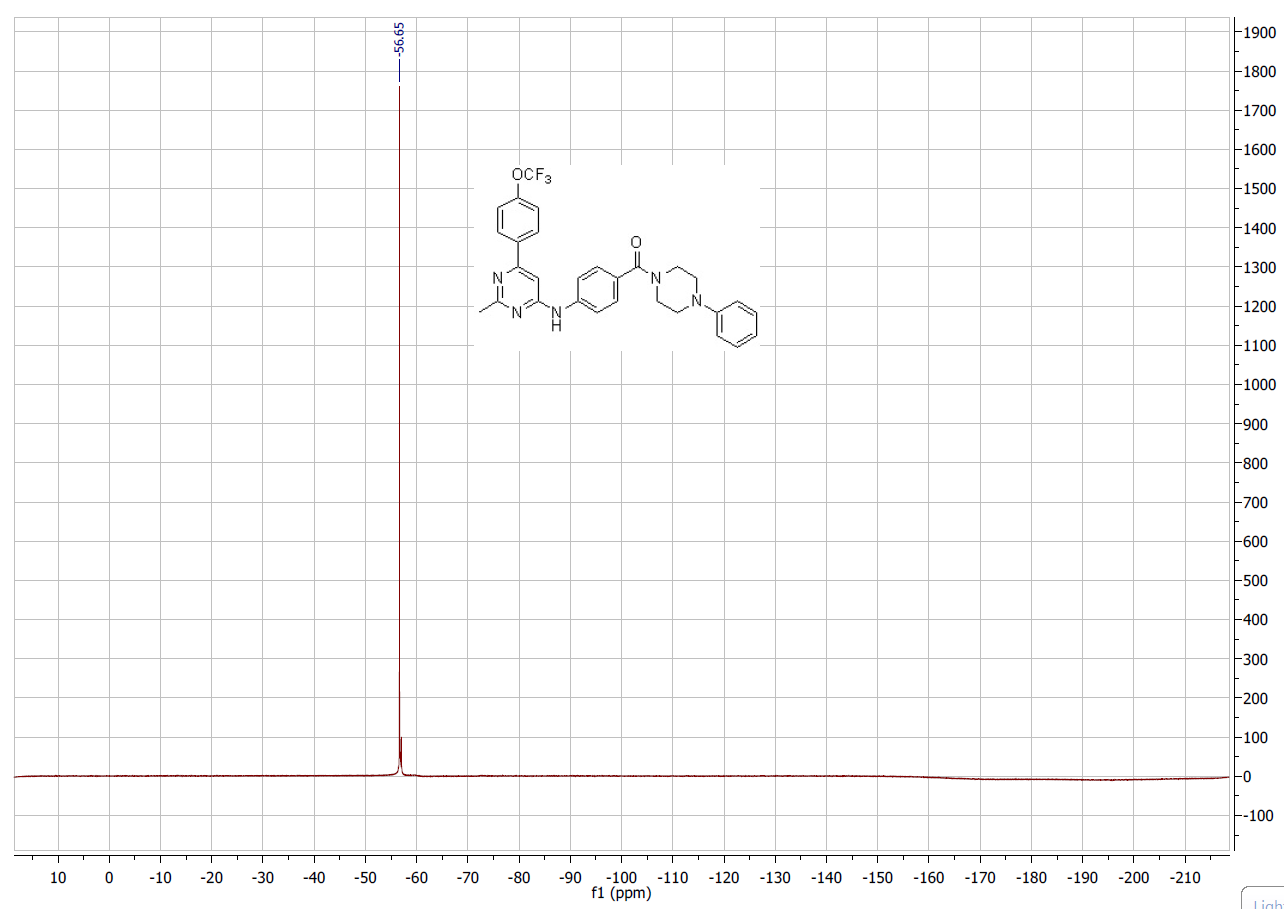


^1^H NMR spectra of compound **6d**
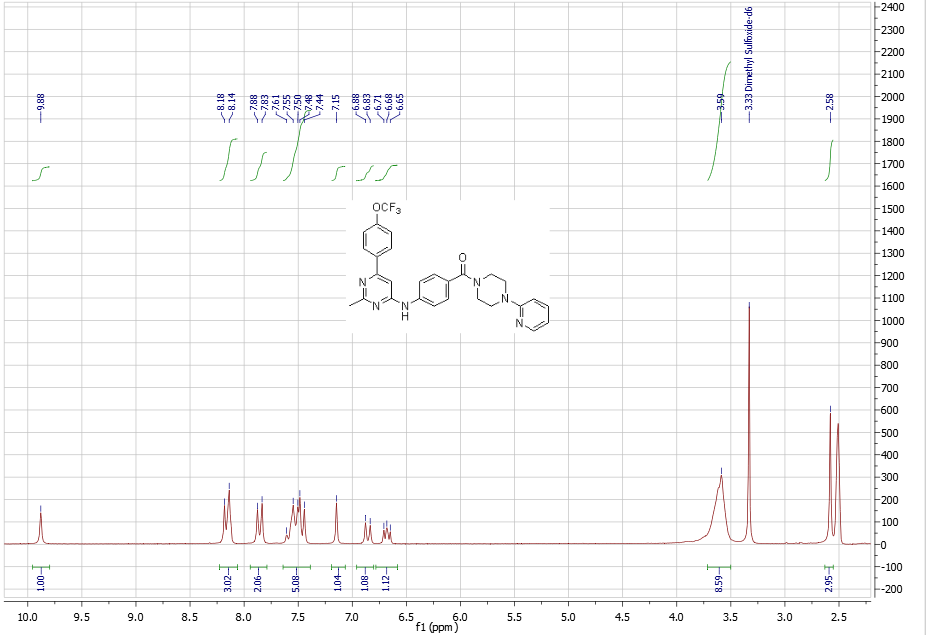


^13^C NMR spectra of compound **6d**


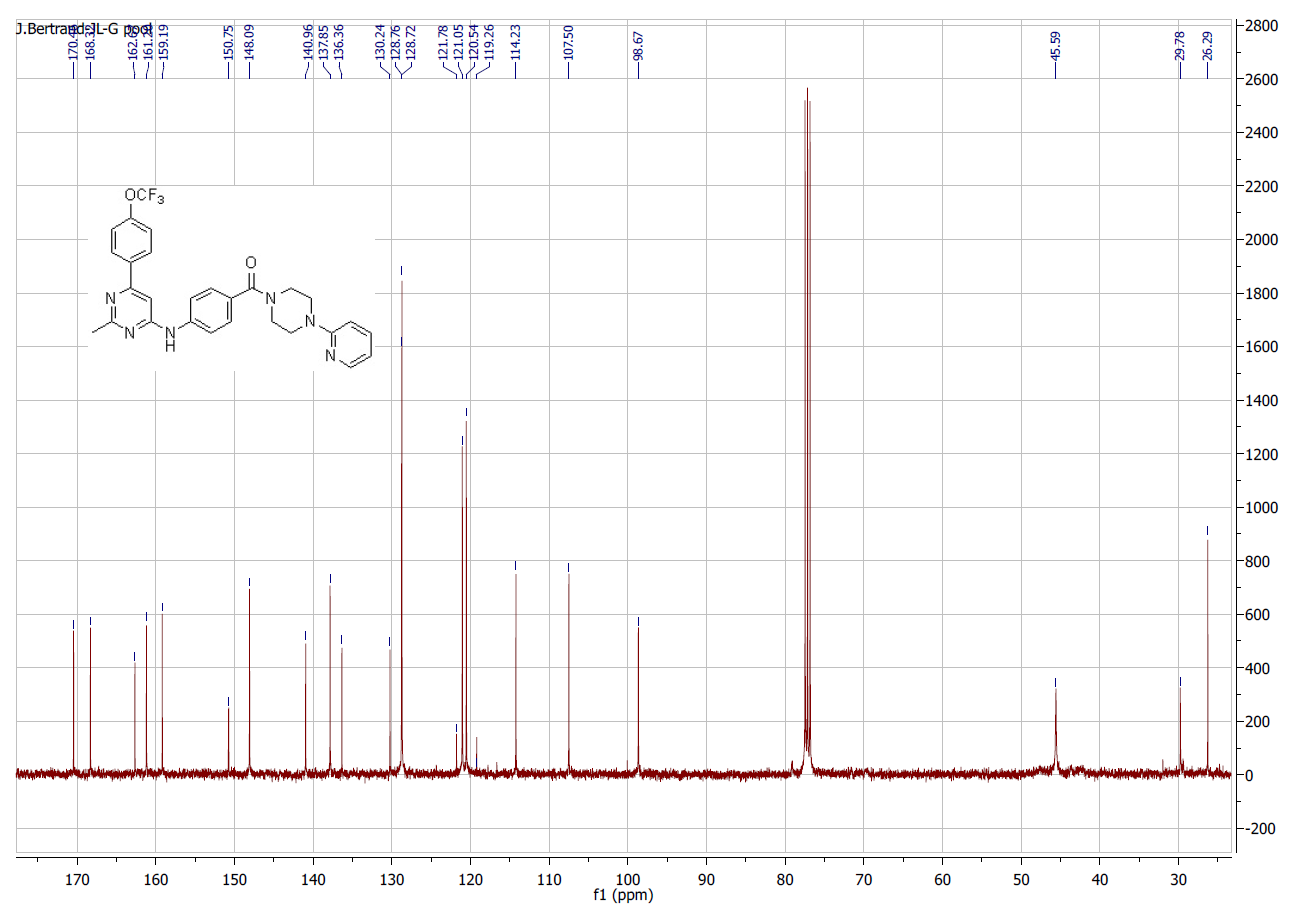


^19^F NMR spectra of compound **6d**


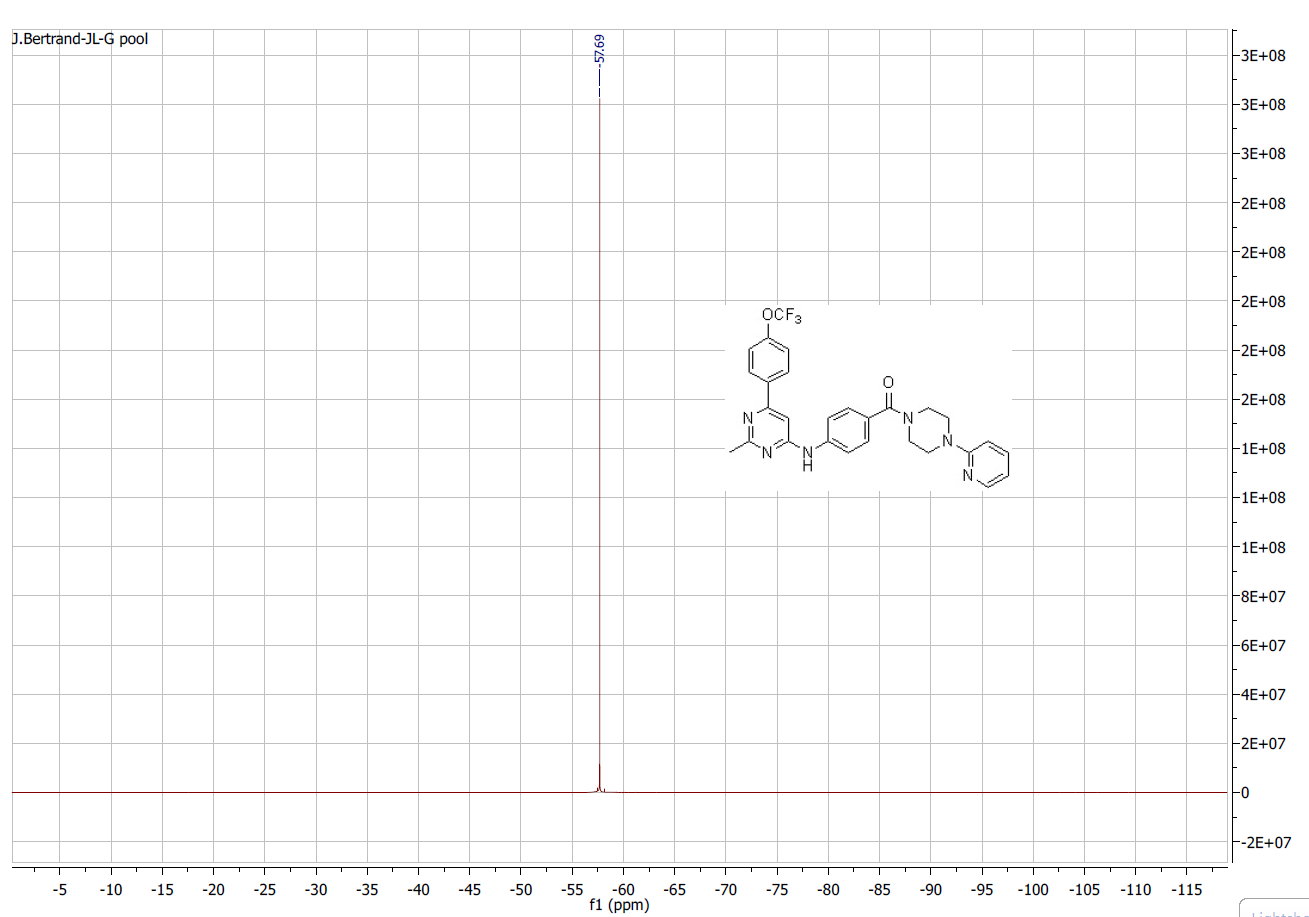


^1^H NMR spectra of compound **6e**


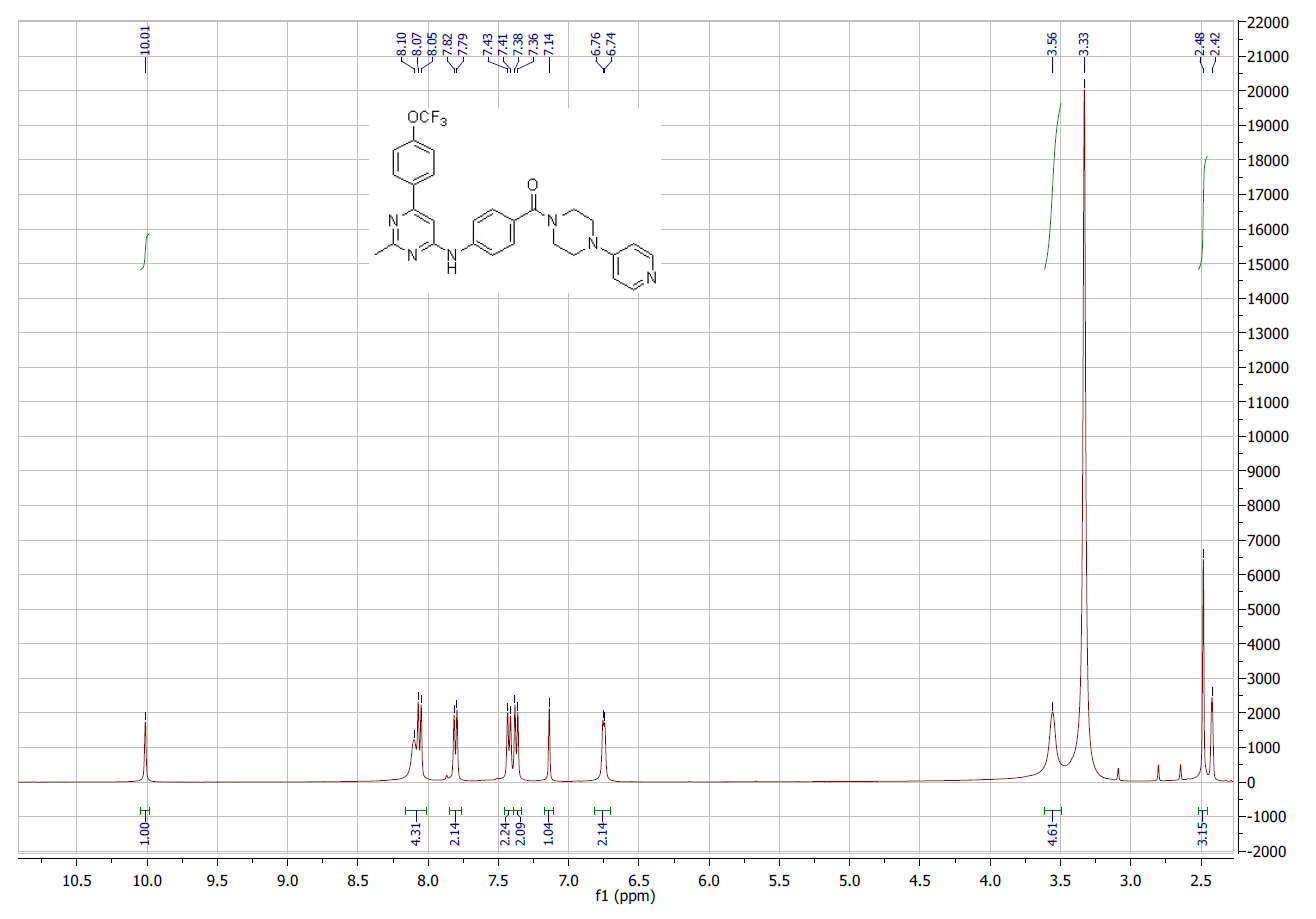


^13^C NMR spectra of compound **6e**


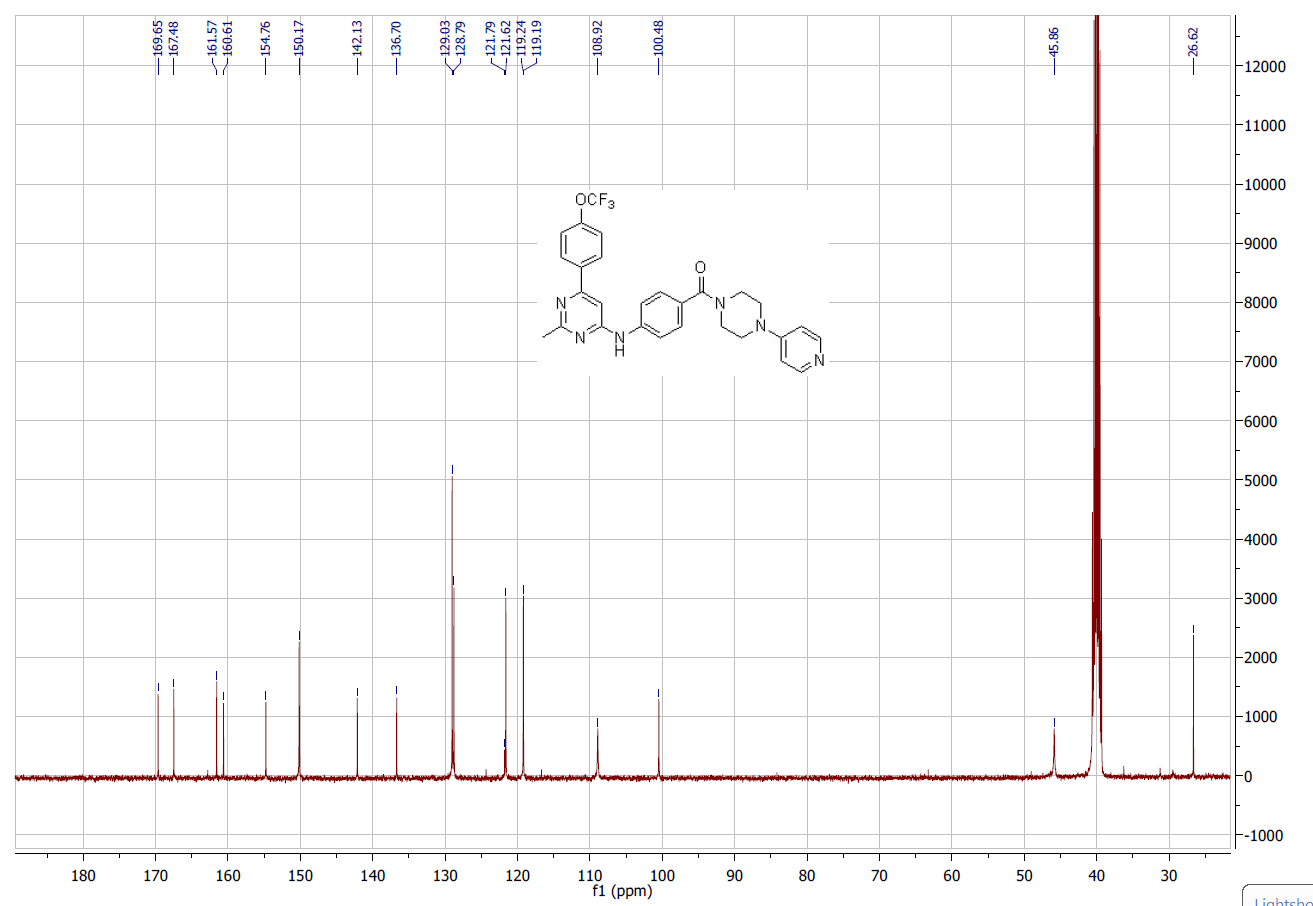


^19^F NMR spectra of compound **6e**


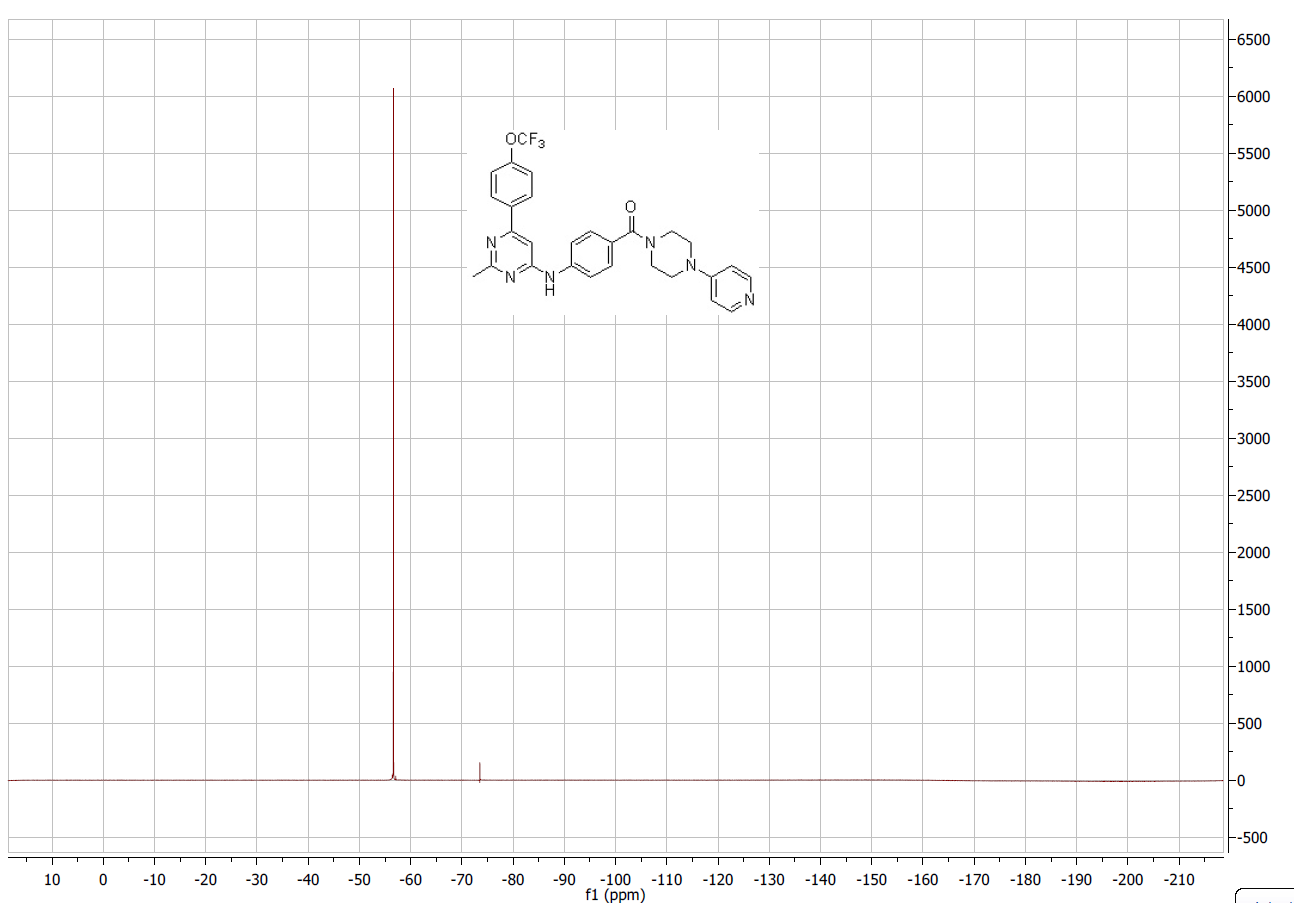


^1^H NMR spectra of compound **6f**

^
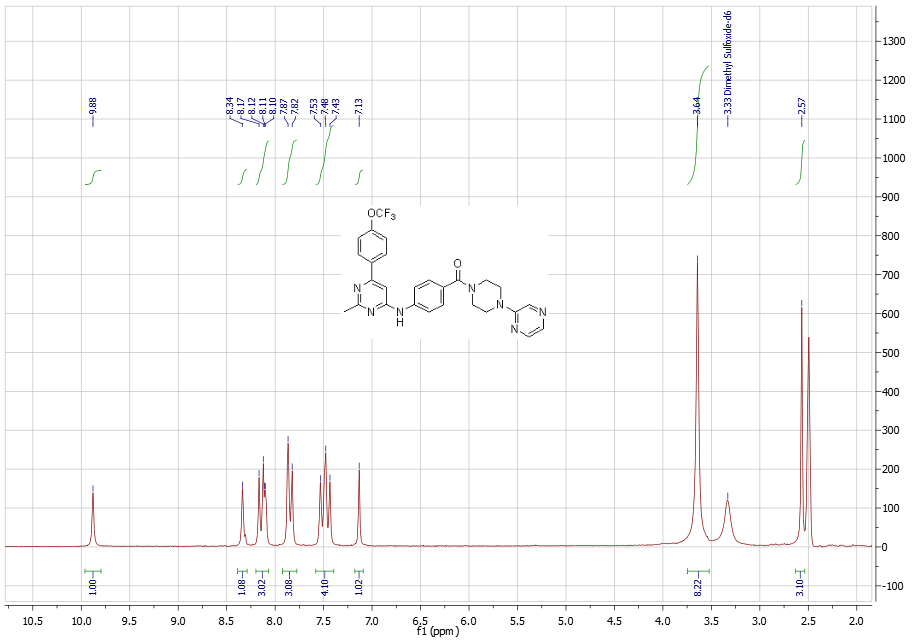
^

^13^C NMR spectra of compound **6f**


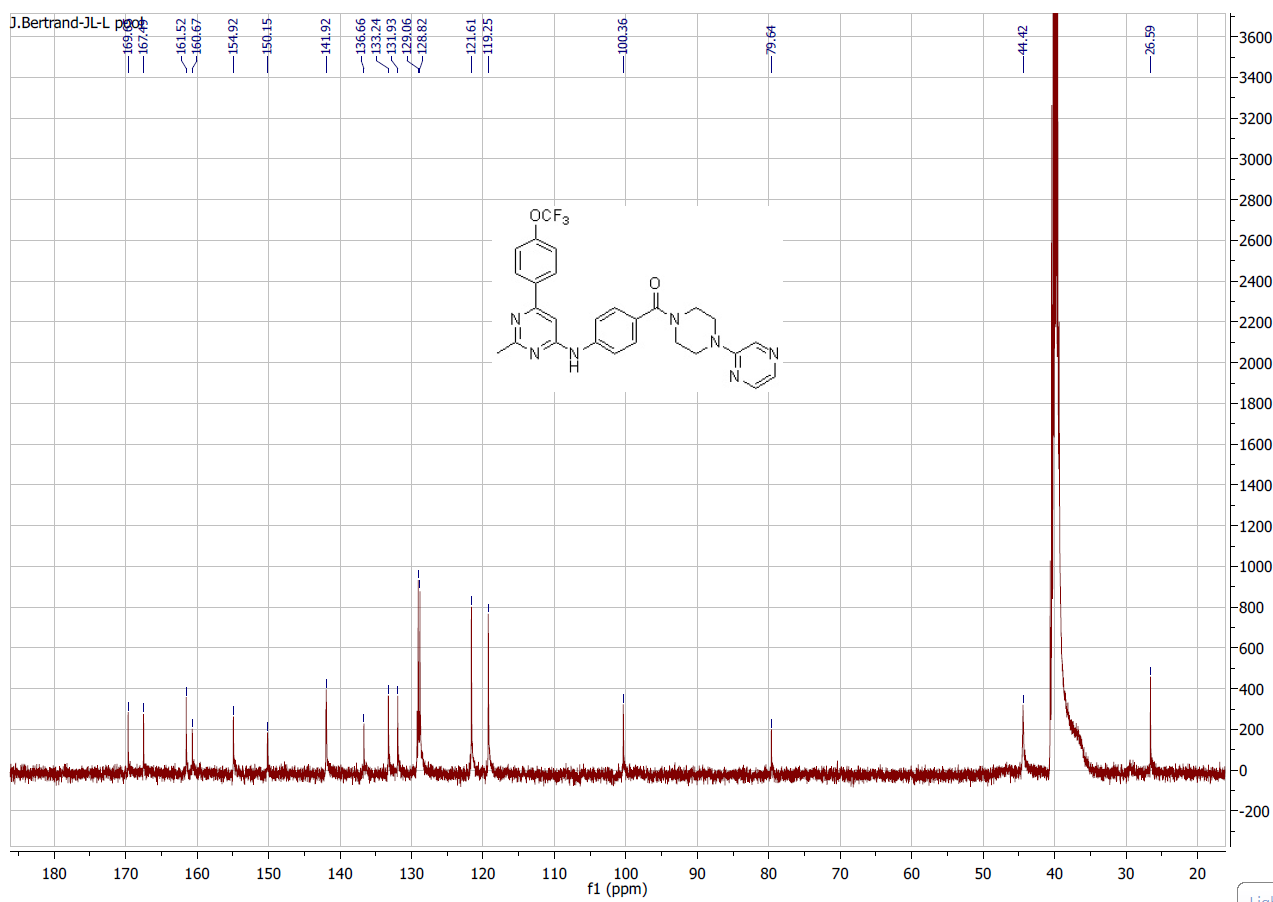


^19^F NMR spectra of compound **6f**


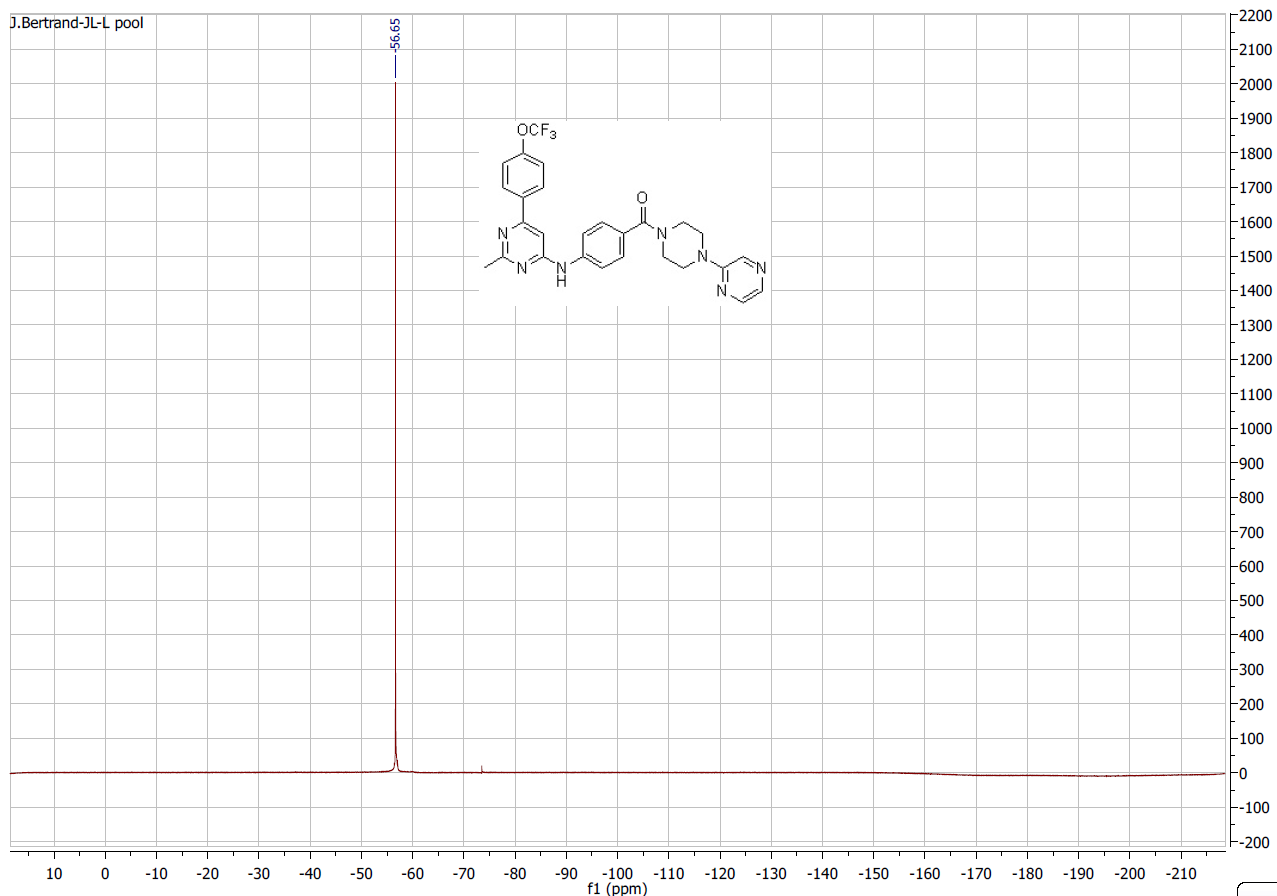


^1^H NMR spectra of compound **6g**


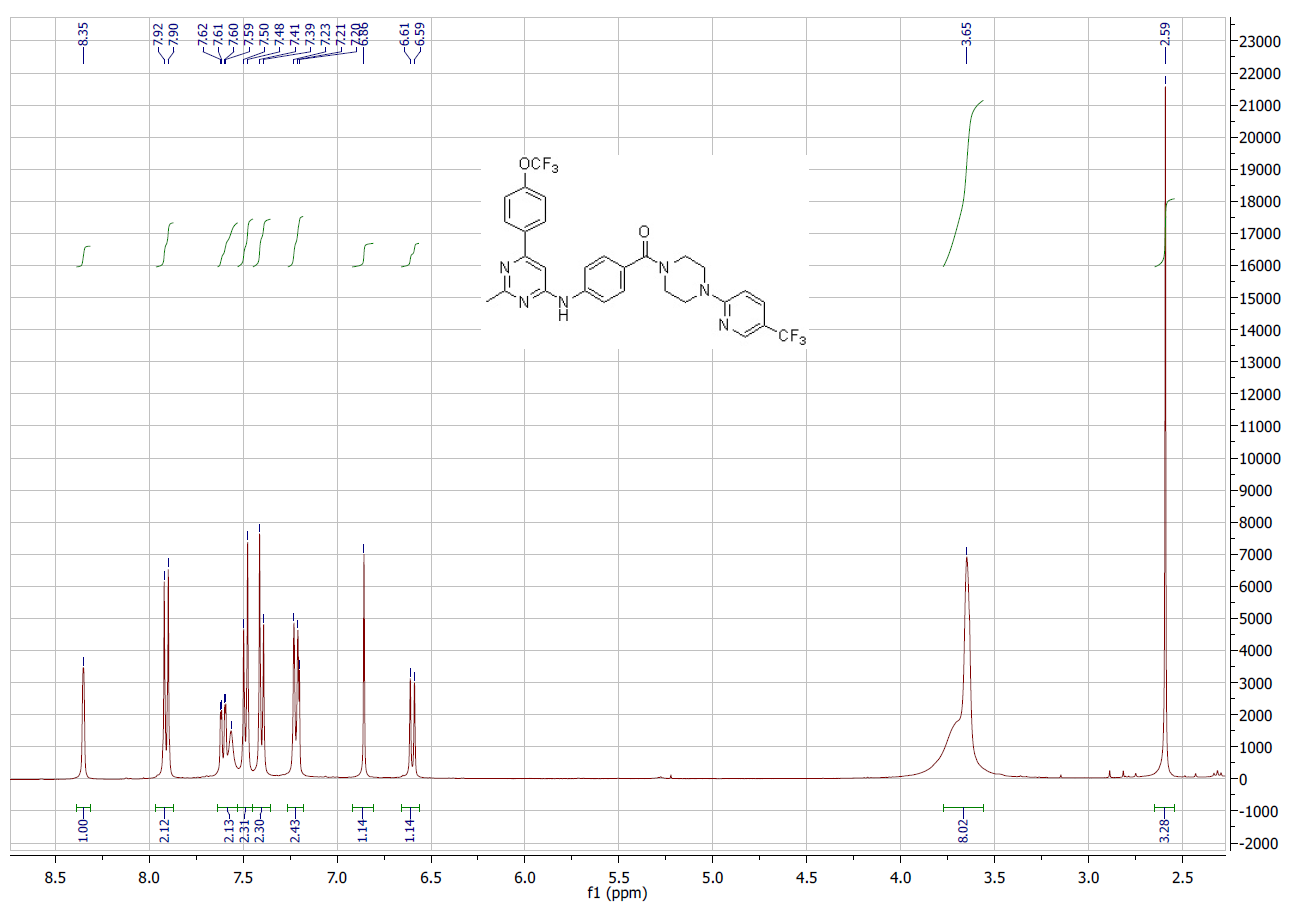


^13^C NMR spectra of compound **6g**


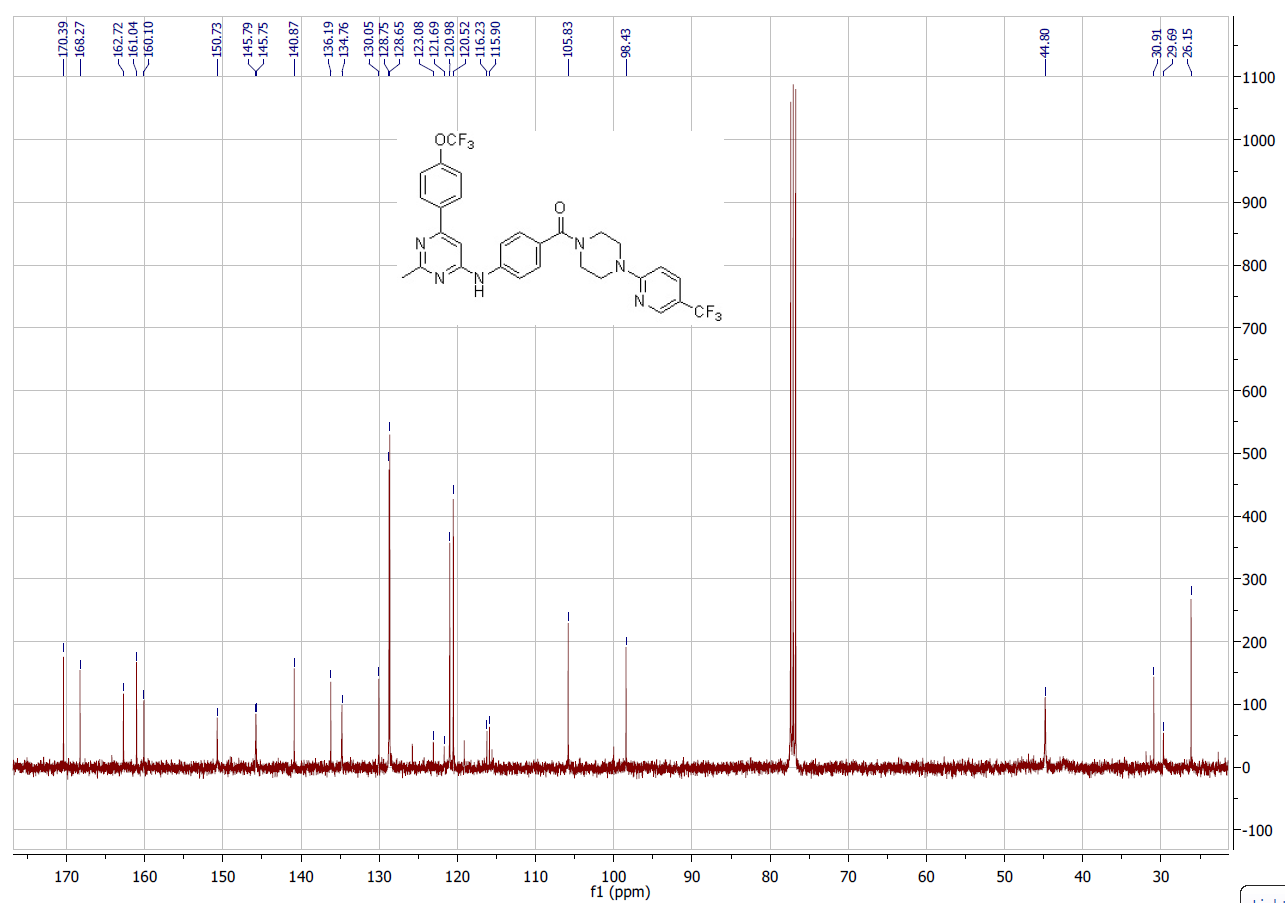


^19^F NMR spectra of compound **6g**


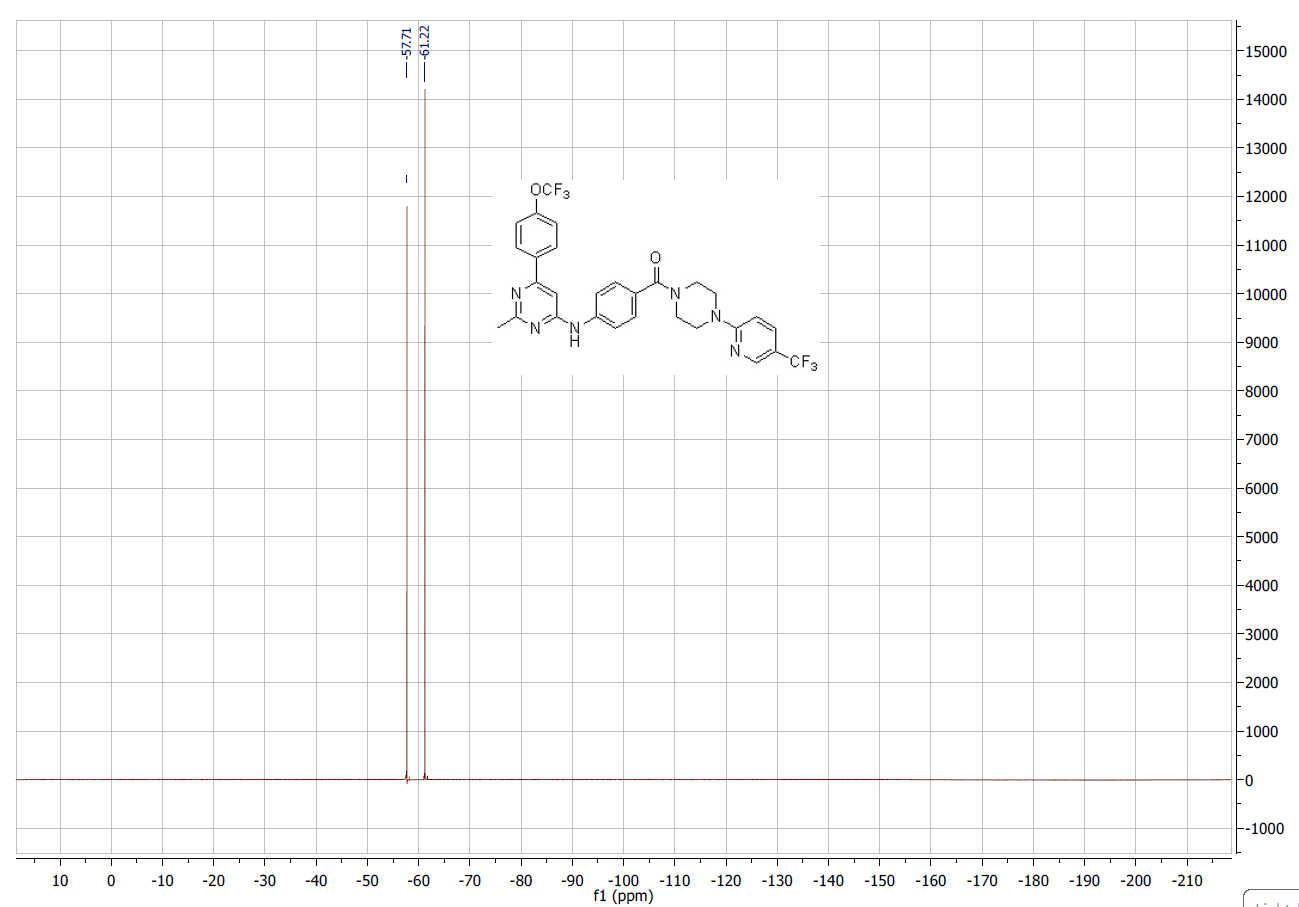


^1^H NMR spectra of compound **6h**


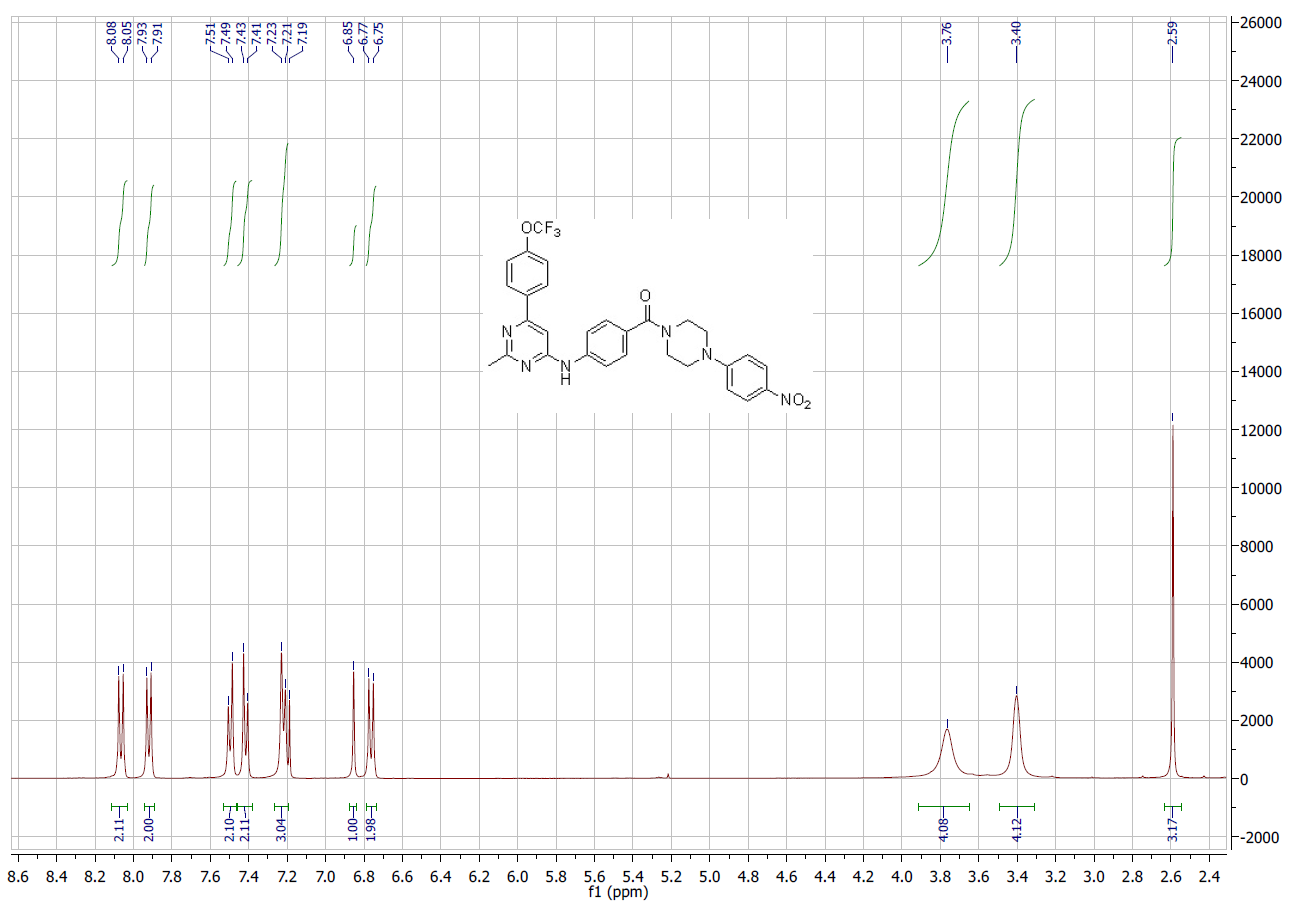


^13^C NMR spectra of compound **6h**


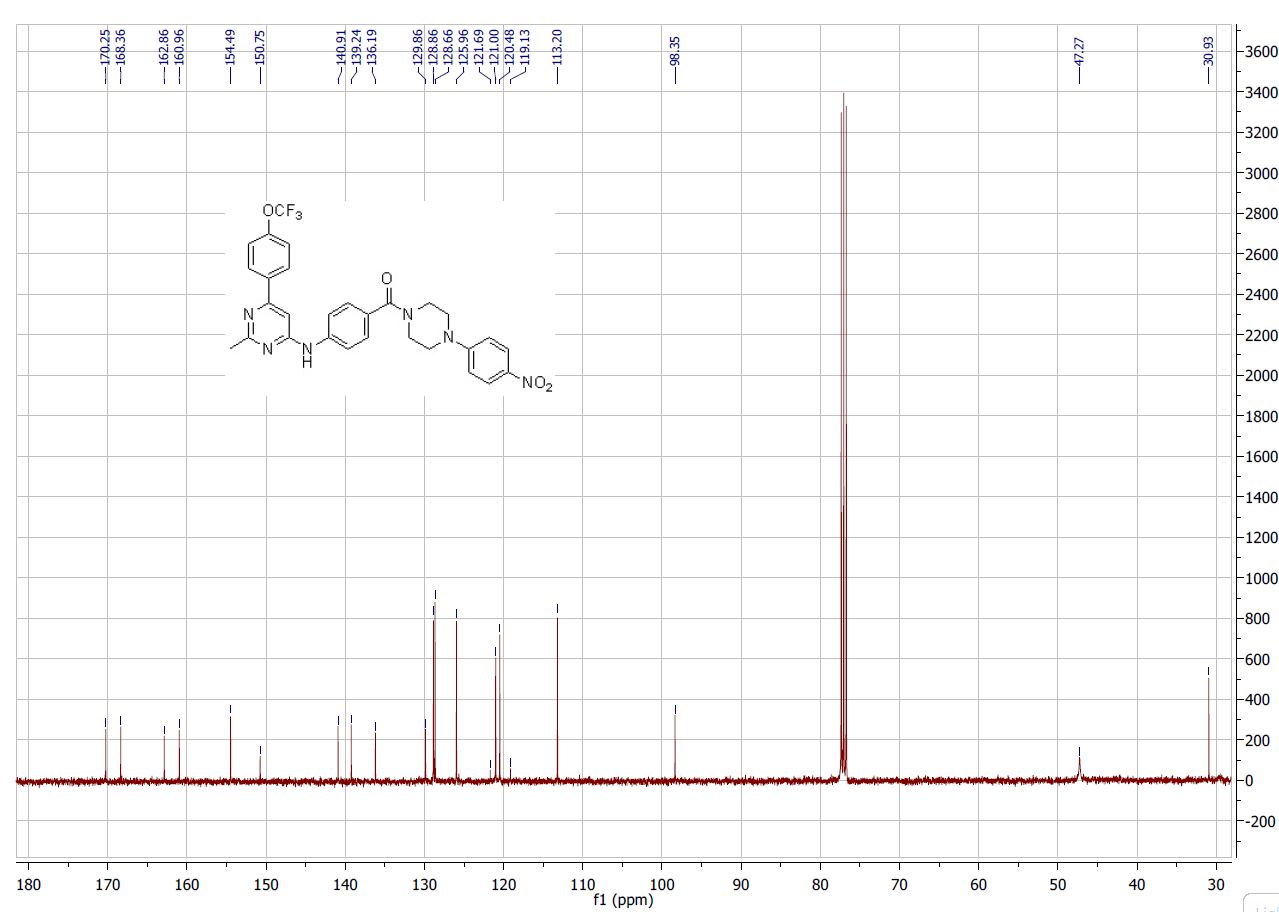


^19^F NMR spectra of compound **6h**


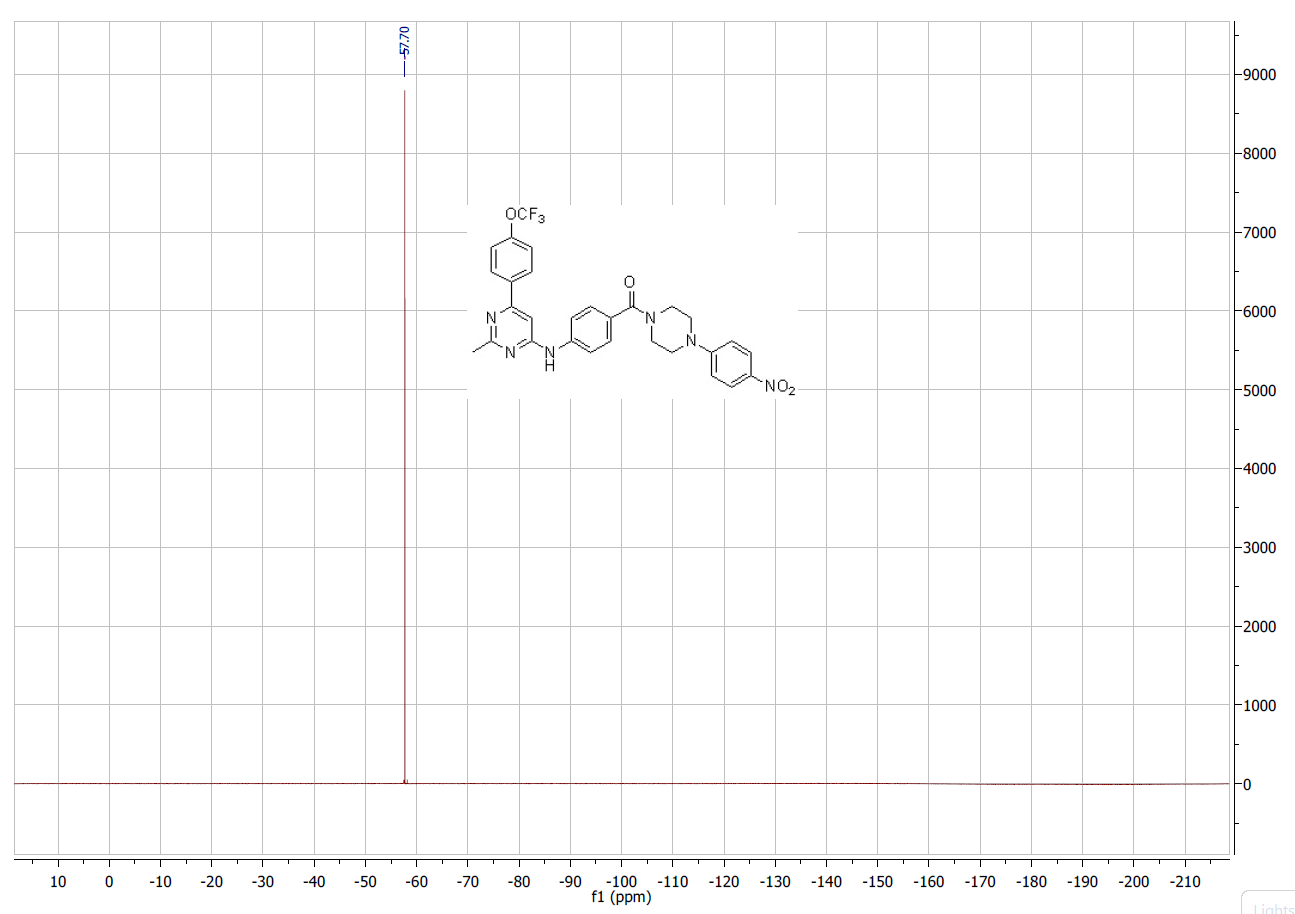


^1^H NMR spectra of compound **6i**


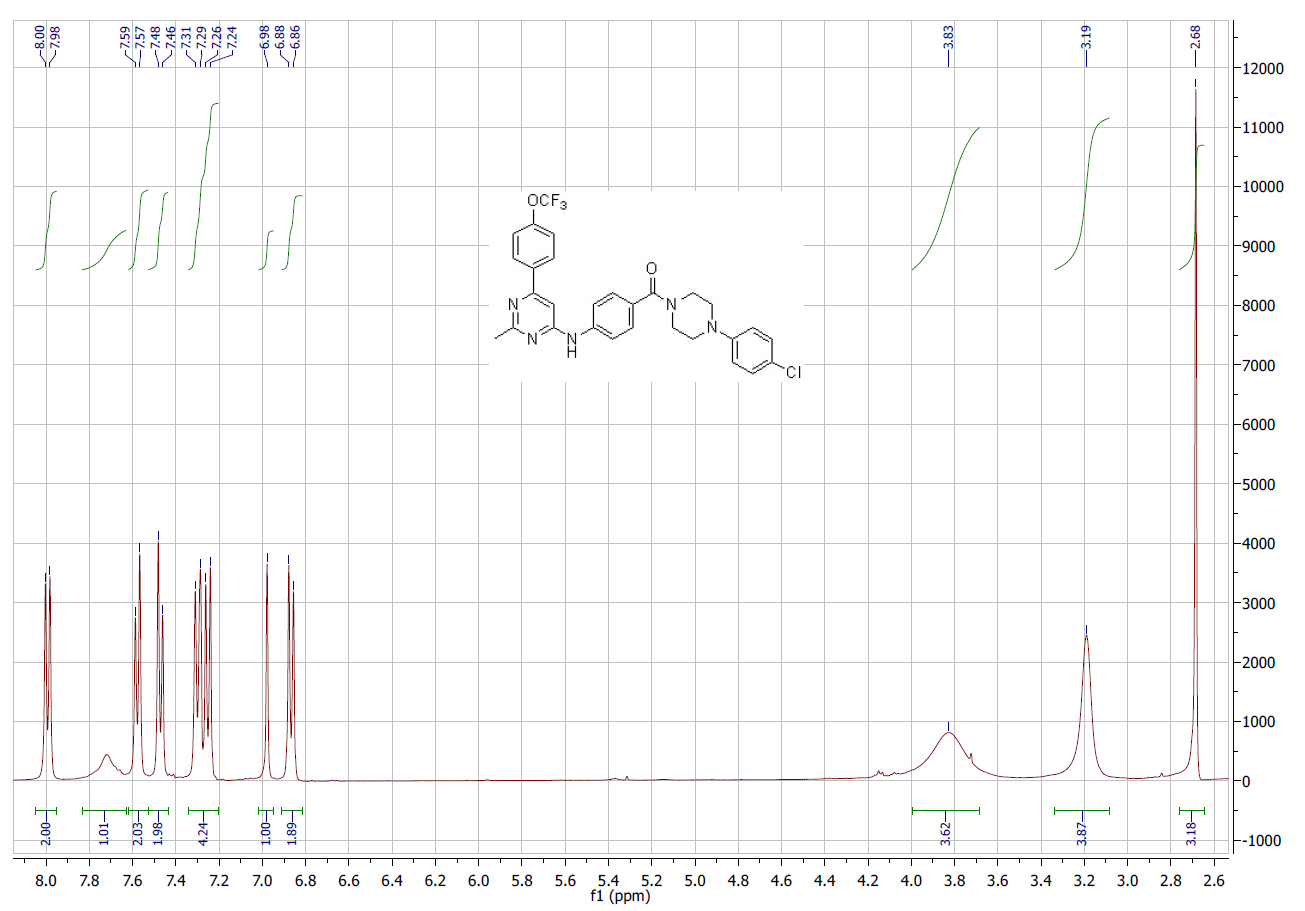


^13^C NMR spectra of compound **6i**


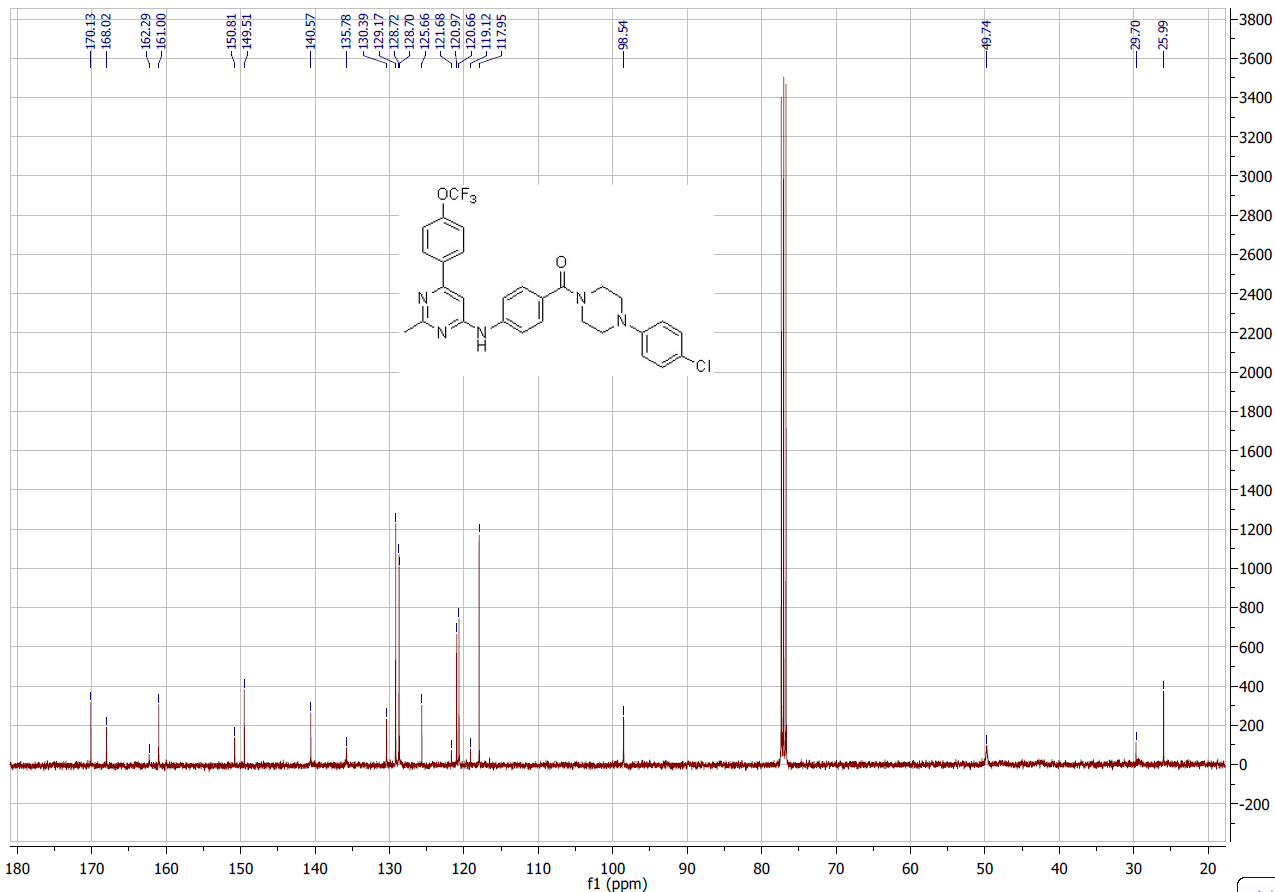


^19^F NMR spectra of compound **6i**


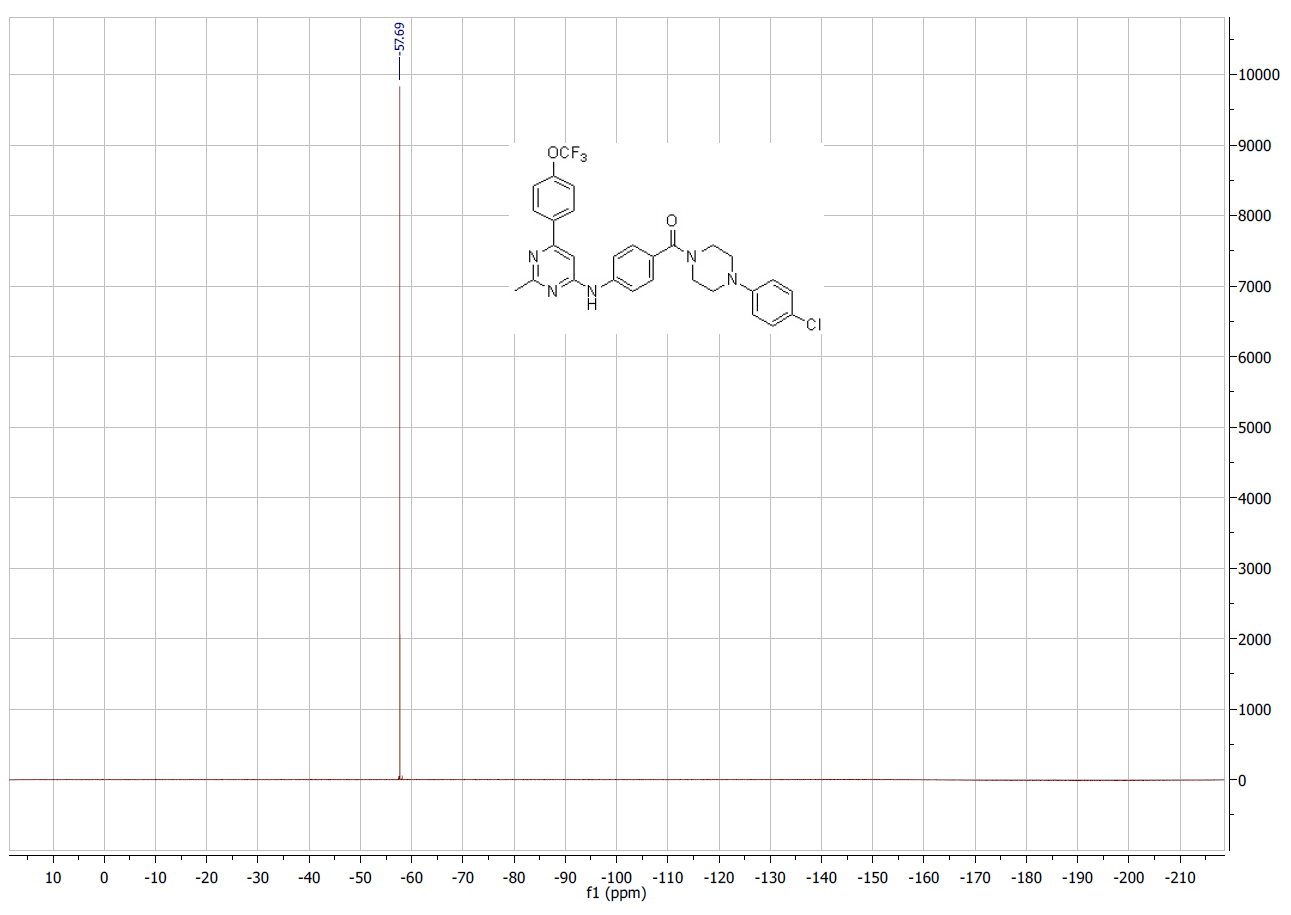


^1^H NMR spectra of compound **6j**


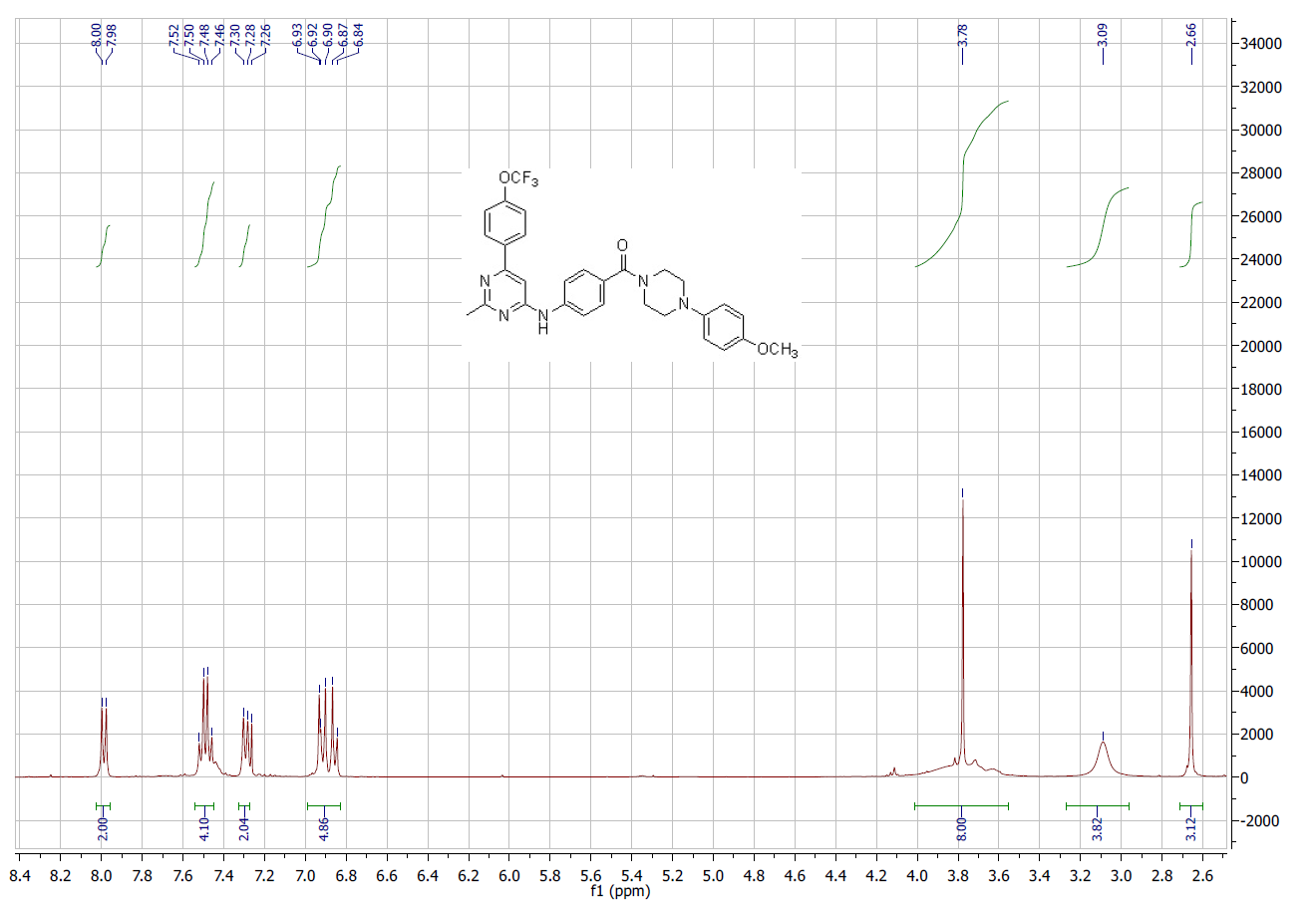


^13^C NMR spectra of compound **6j**


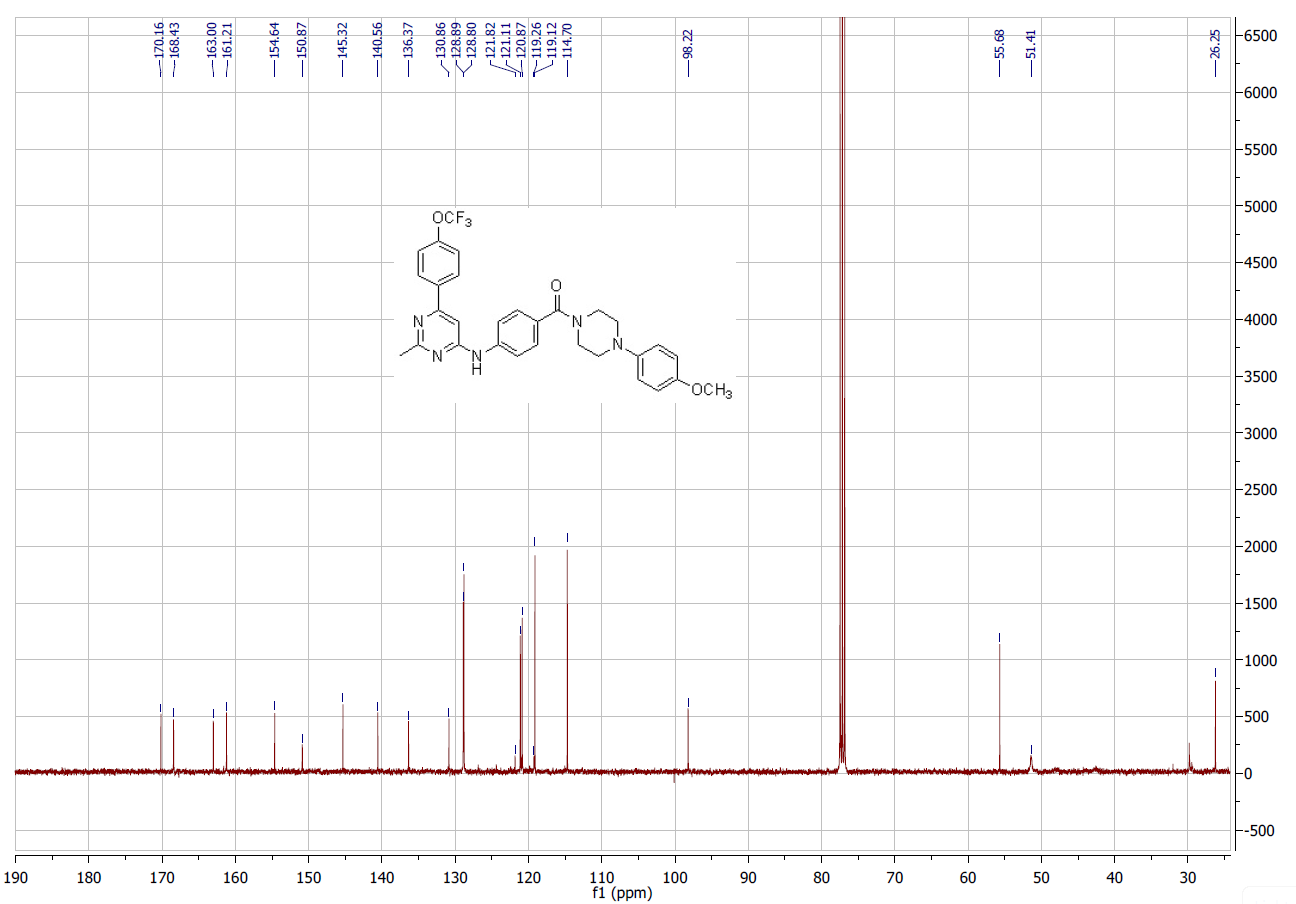


^19^F NMR spectra of compound **6j**


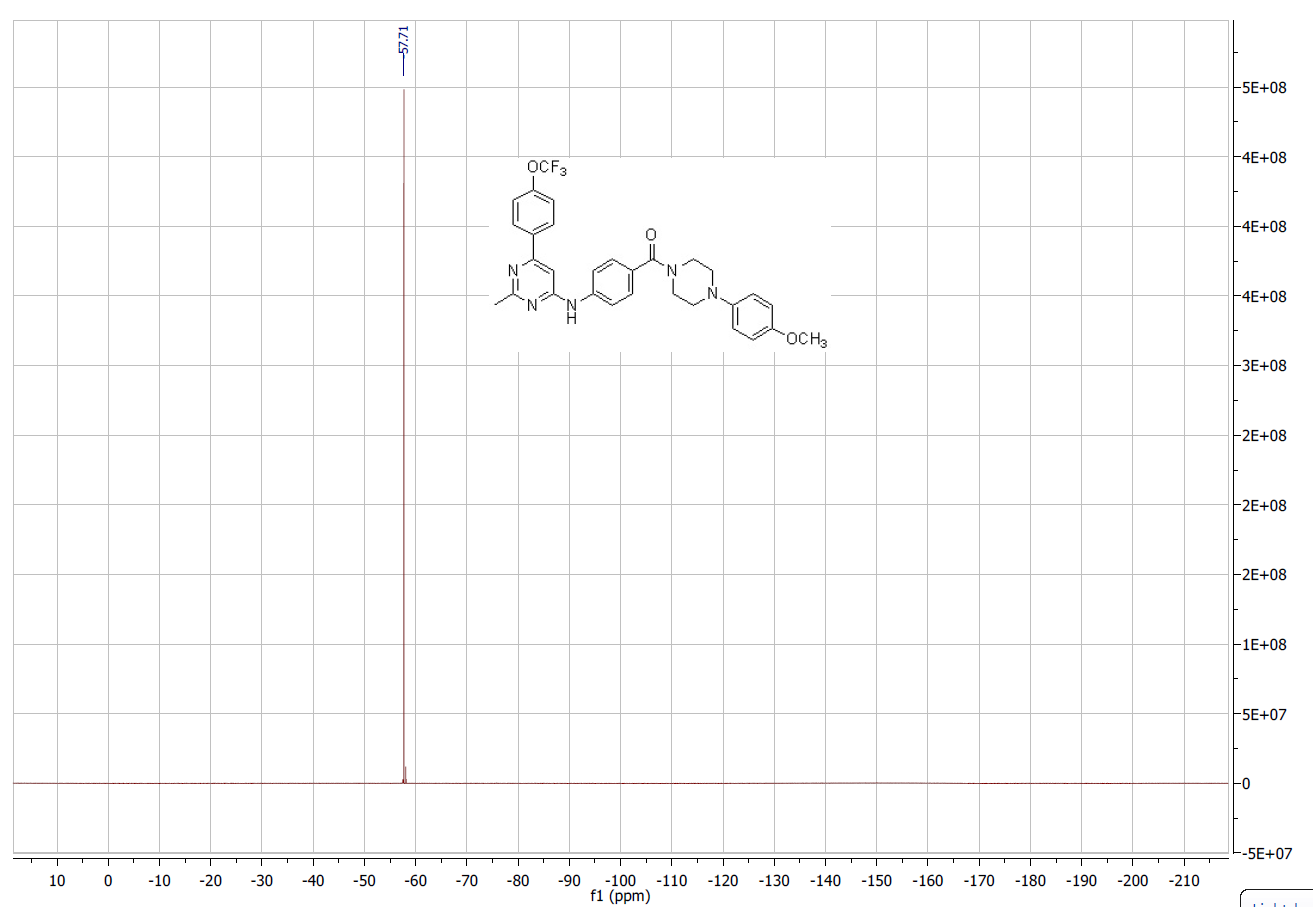


^1^H NMR spectra of compound **6k**


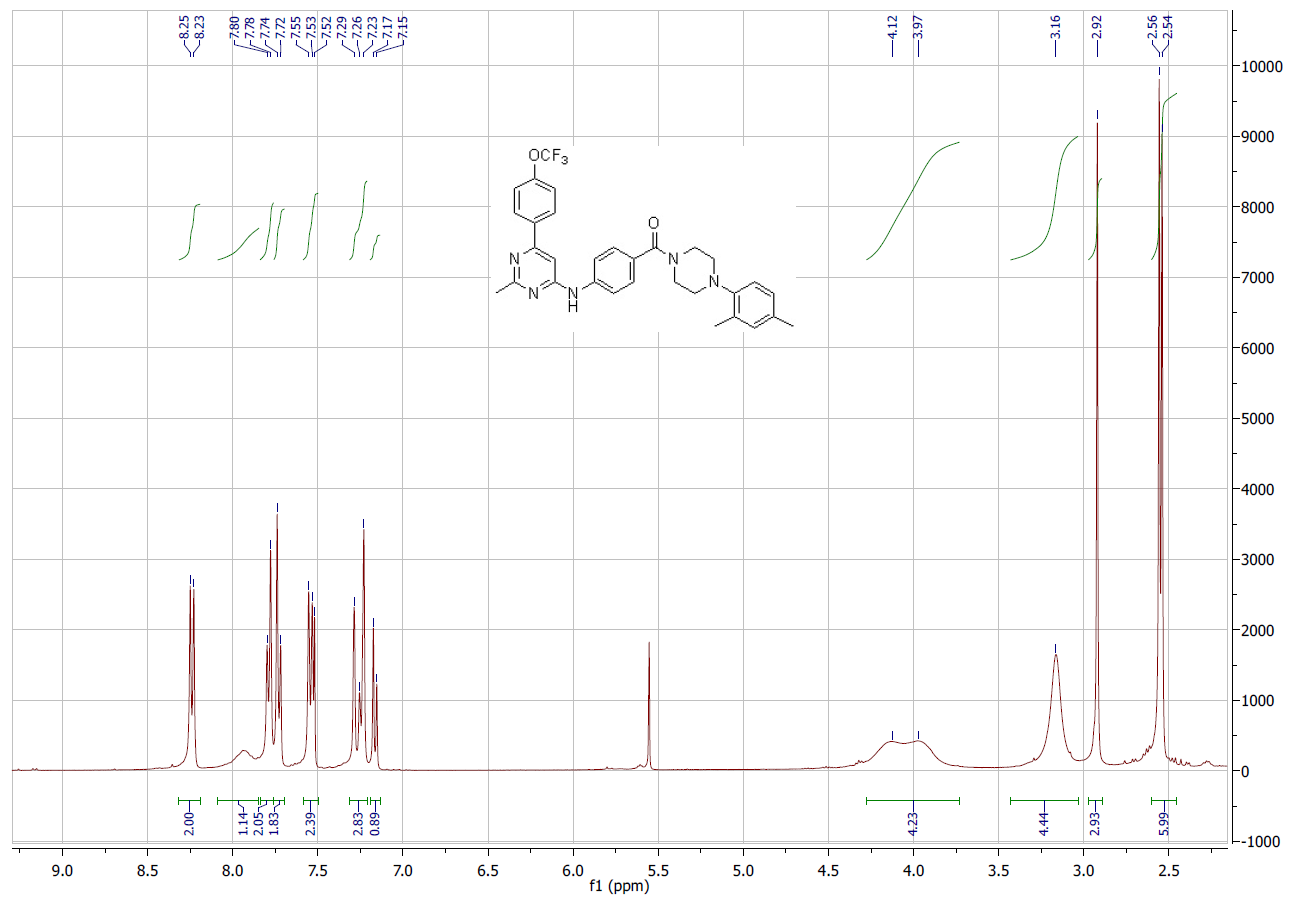


^13^C NMR spectra of compound **6k**


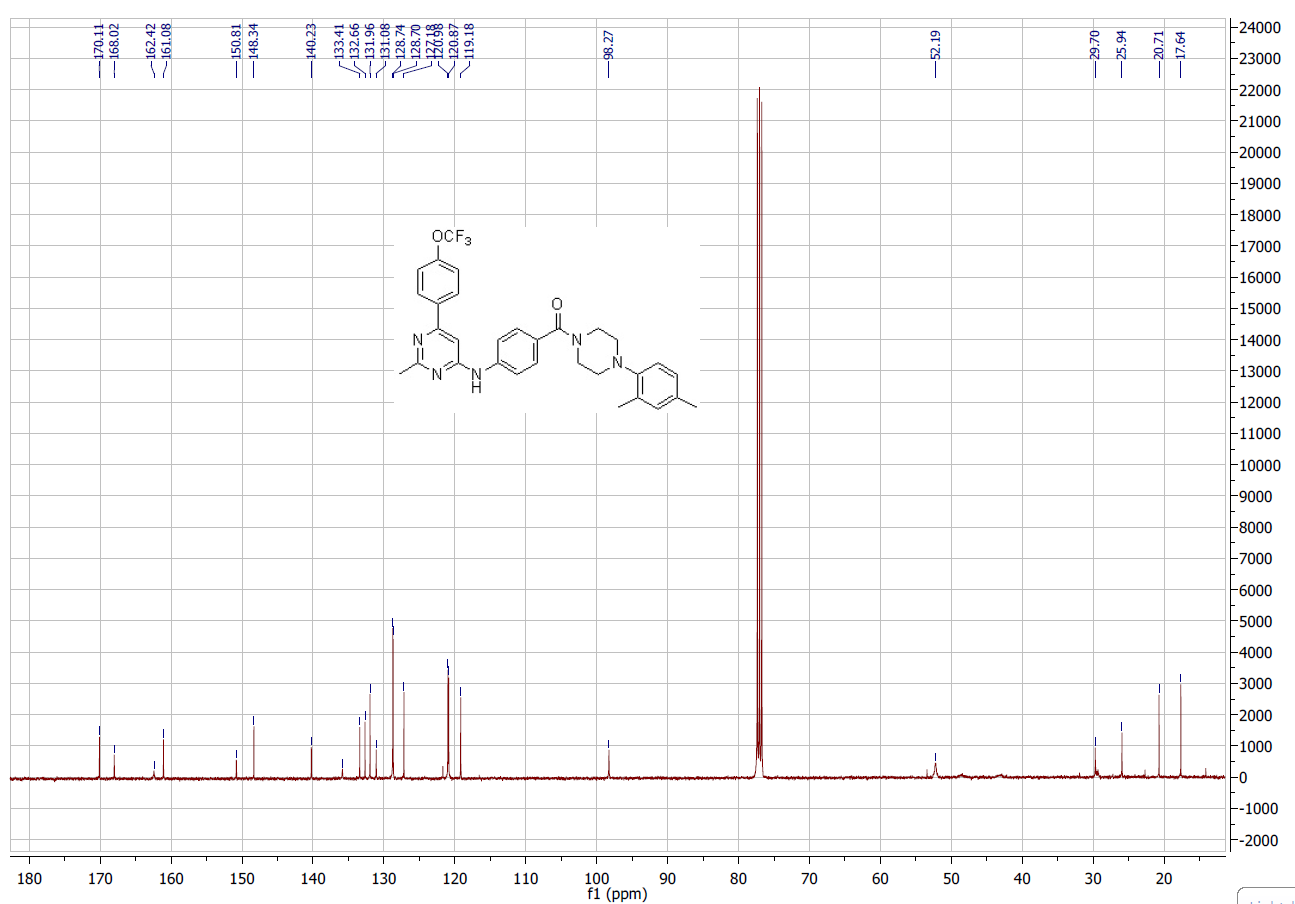


^19^F NMR spectra of compound **6k**


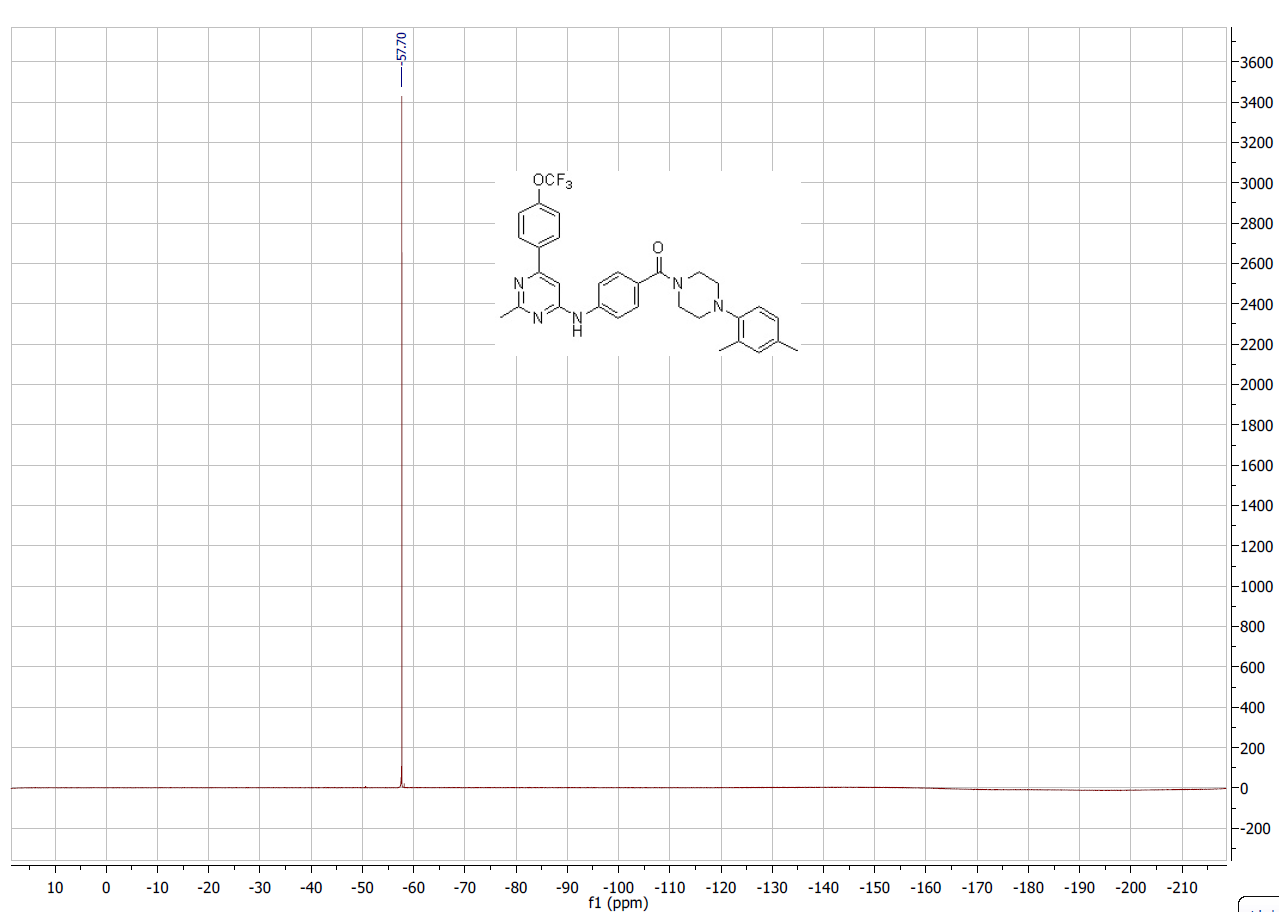


^1^H NMR spectra of compound **6l**


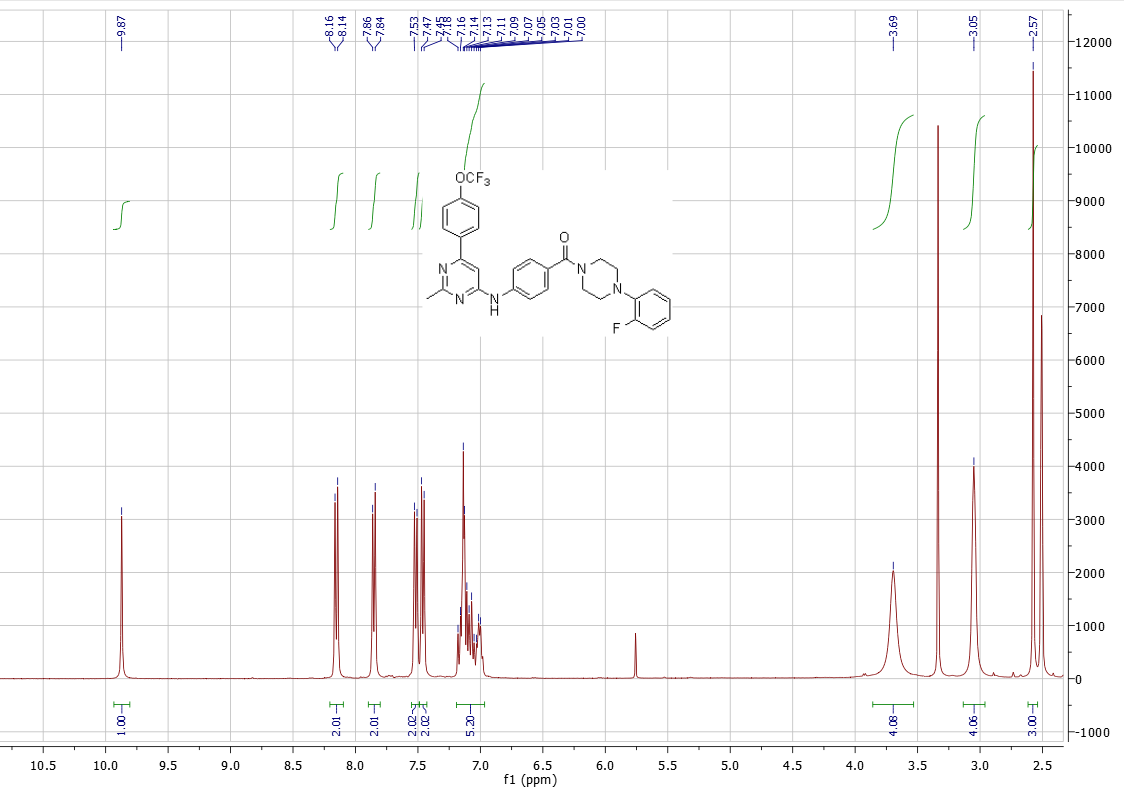


^13^C NMR spectra of compound **6l**


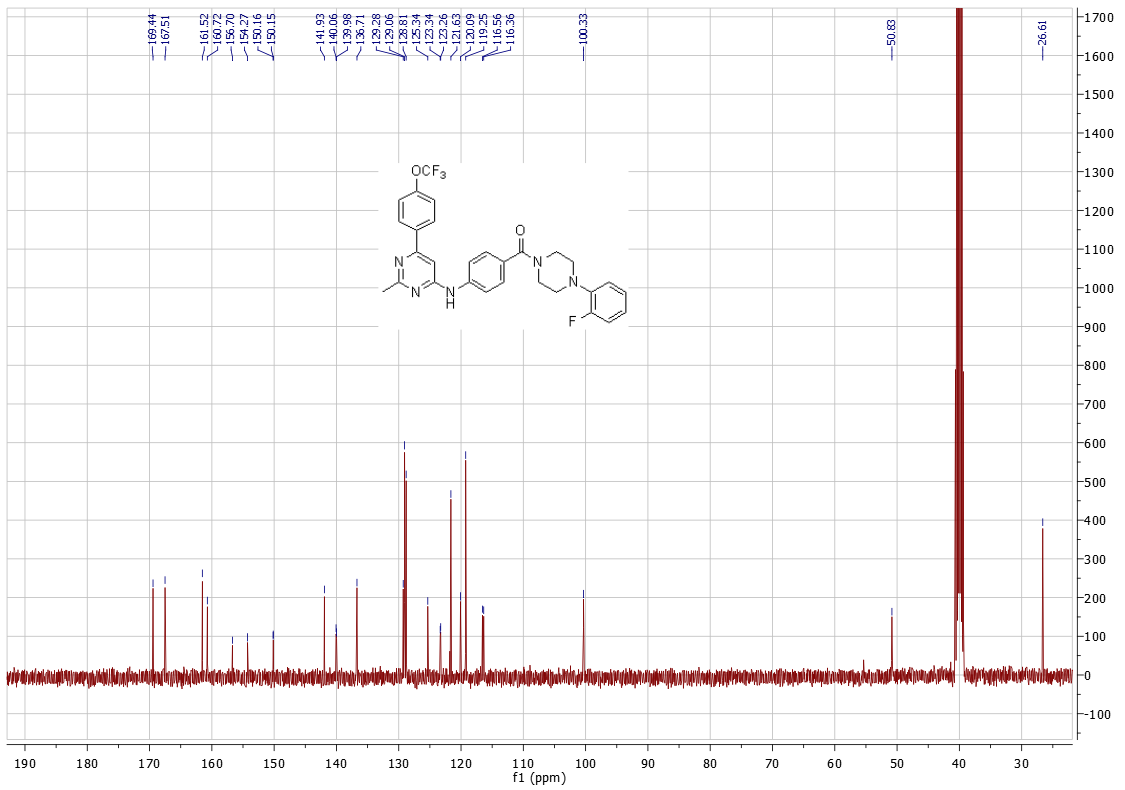


^19^F NMR spectra of compound **6l**


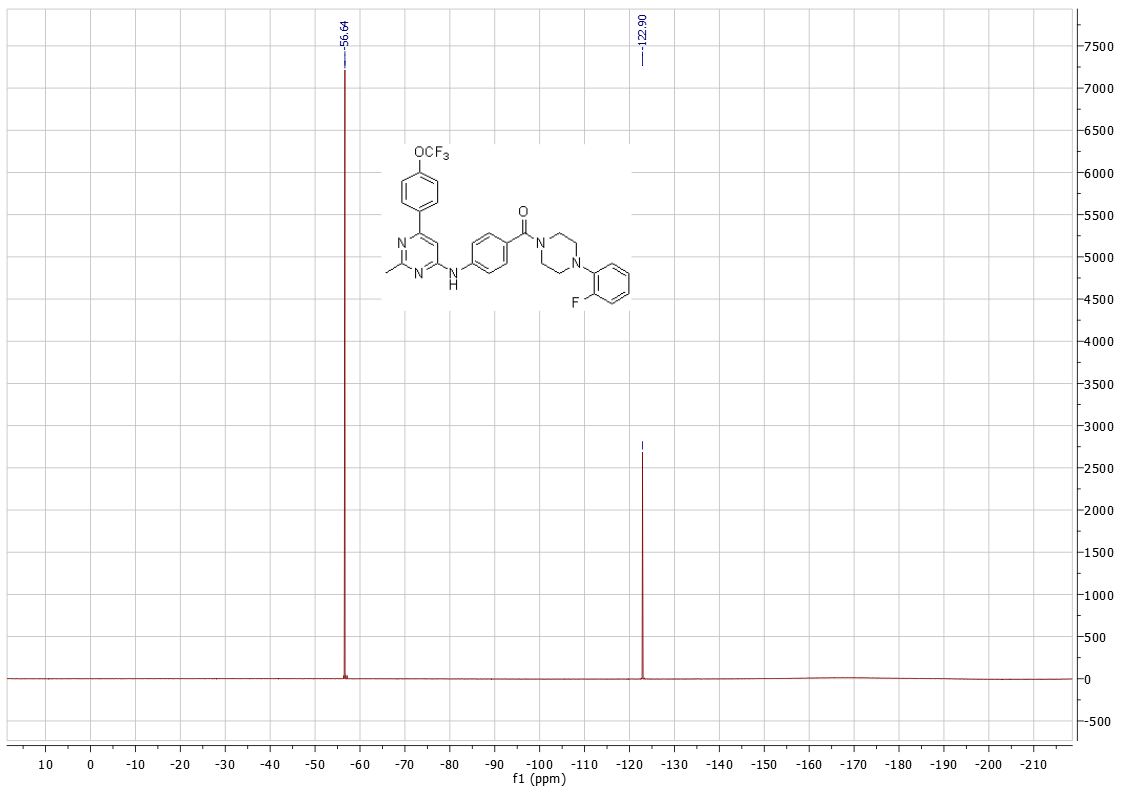


Mass spectra of compound **6a**

Mass spectra of compound **6b**

Mass spectra of compound **6c**

Mass spectra of compound **6d**

Mass spectra of compound **6e**

 Mass spectra of compound **6f**

Mass spectra of compound **6g**

Mass spectra of compound **6h**

Mass spectra of compound **6i**

Mass spectra of compound **6j**

Mass spectra of compound **6k**

Mass spectra of compound **6l**

**HPLC compound 6a**


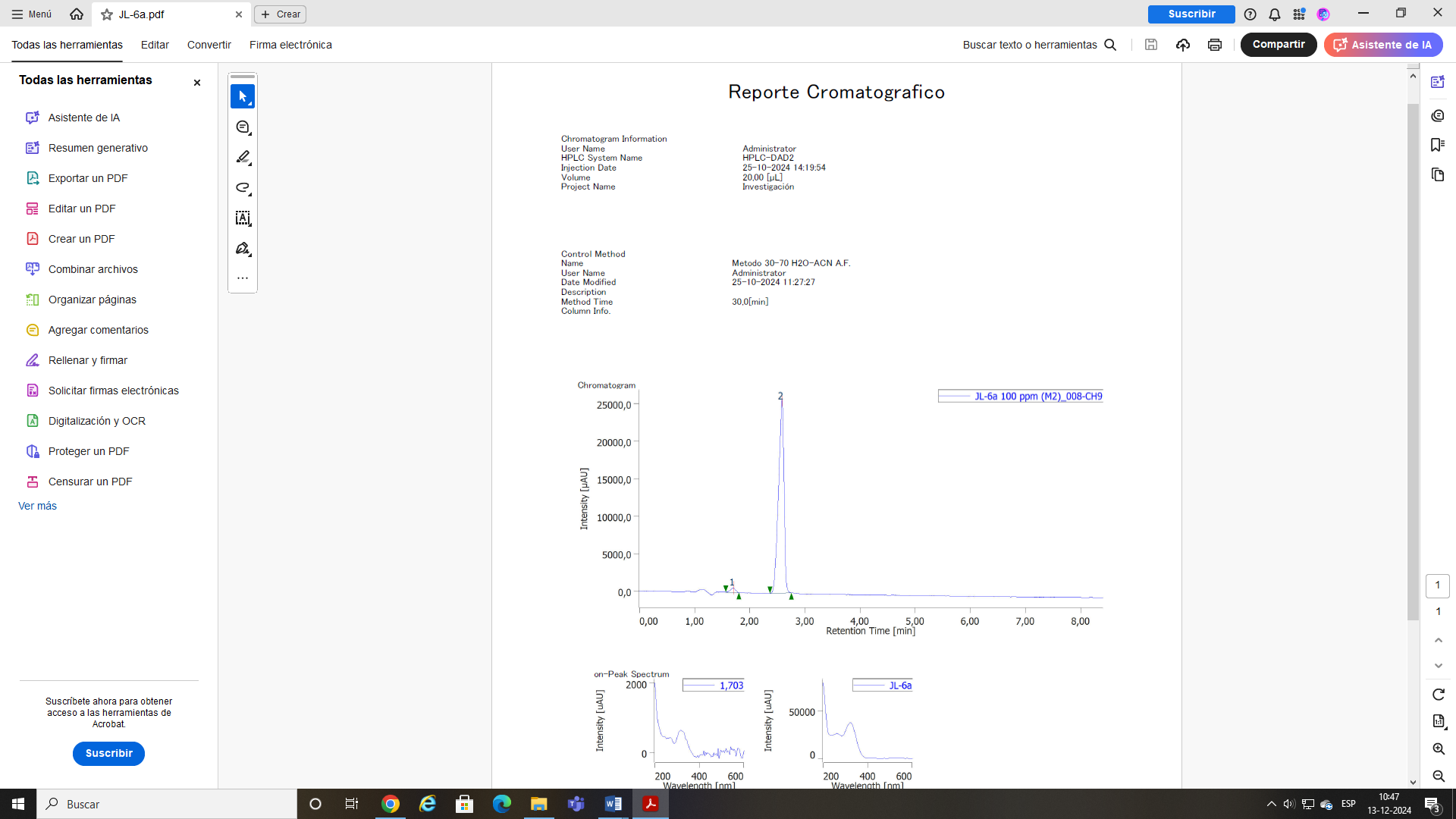


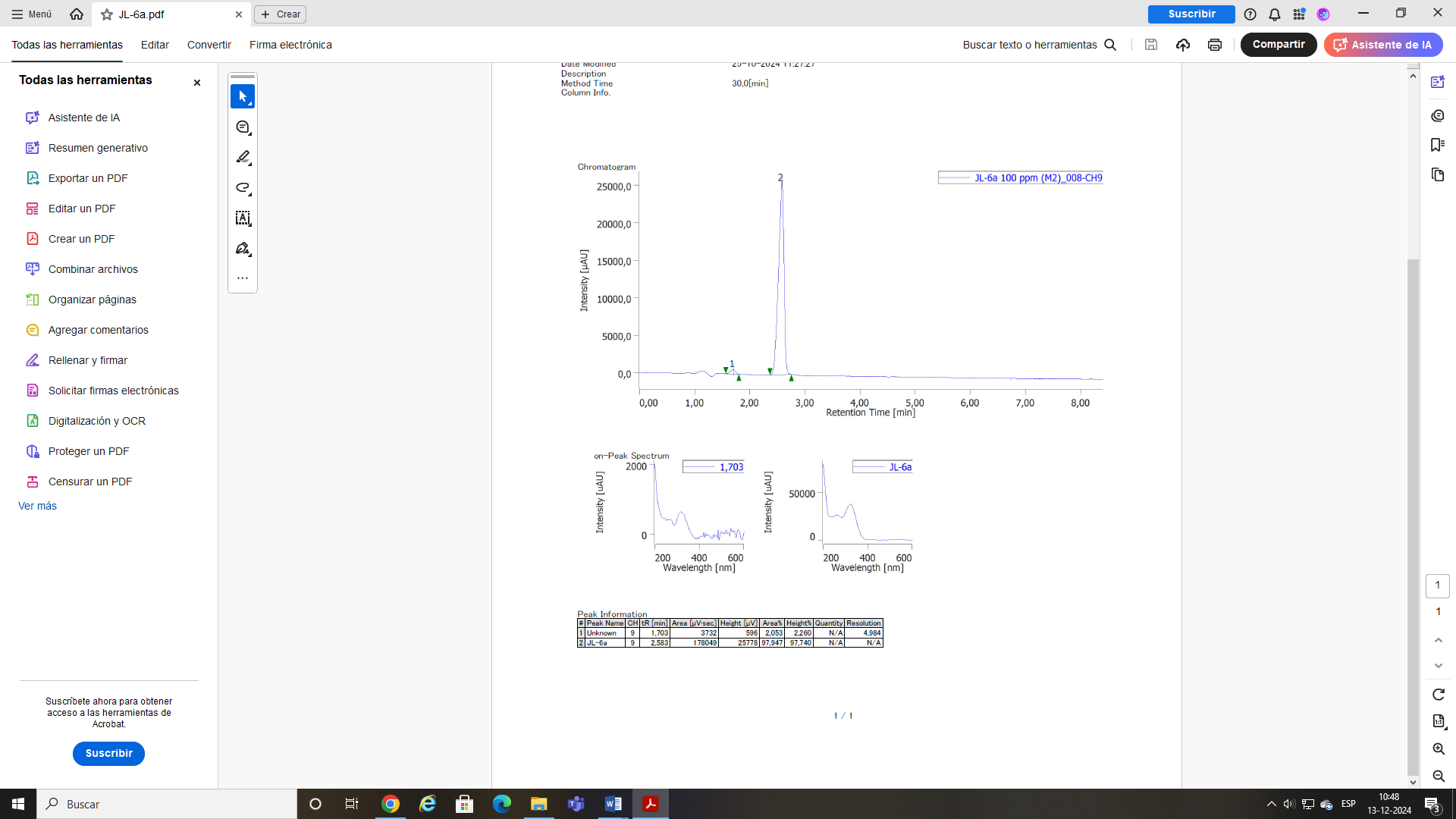


**HPLC compound 6b**


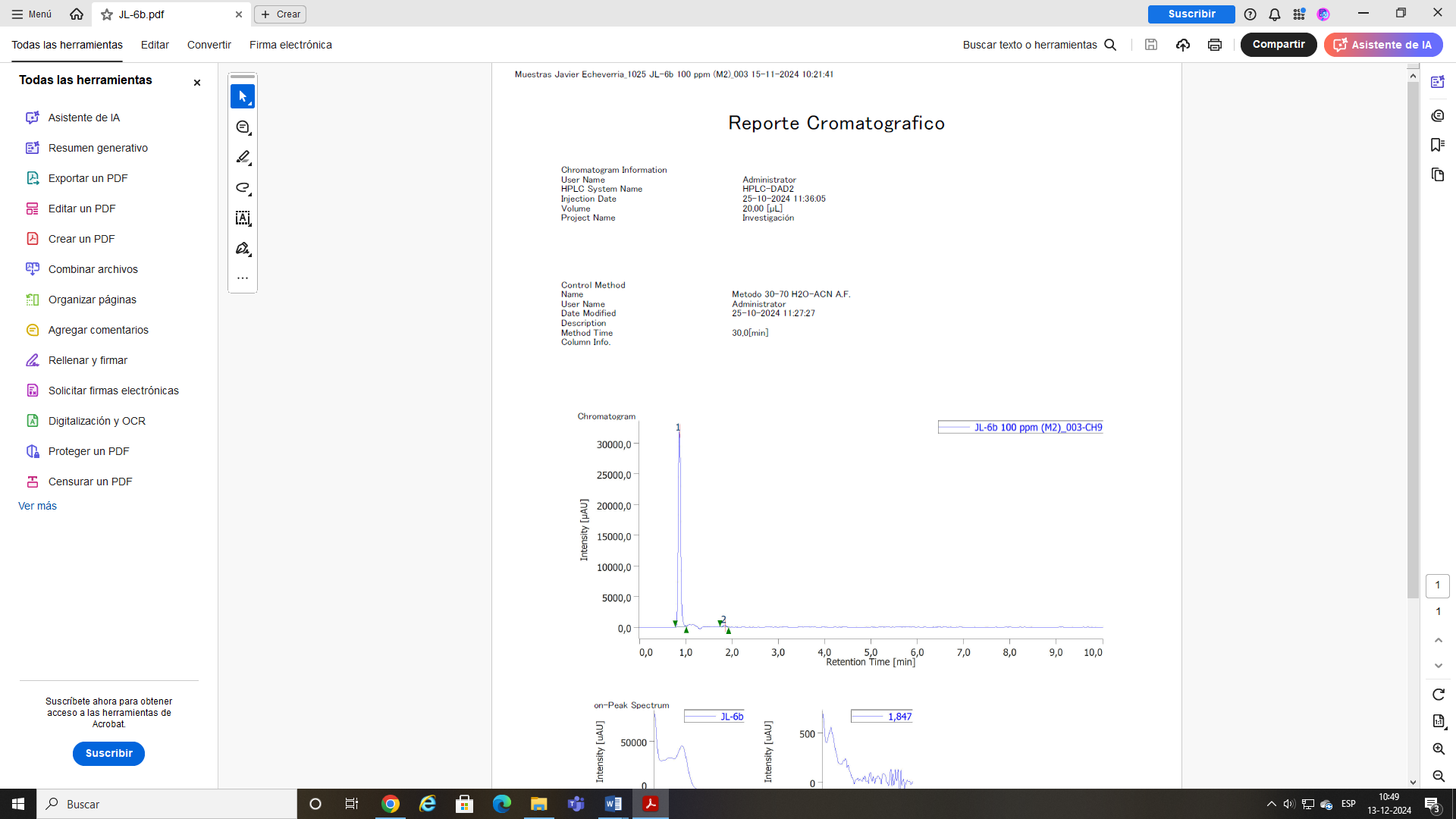


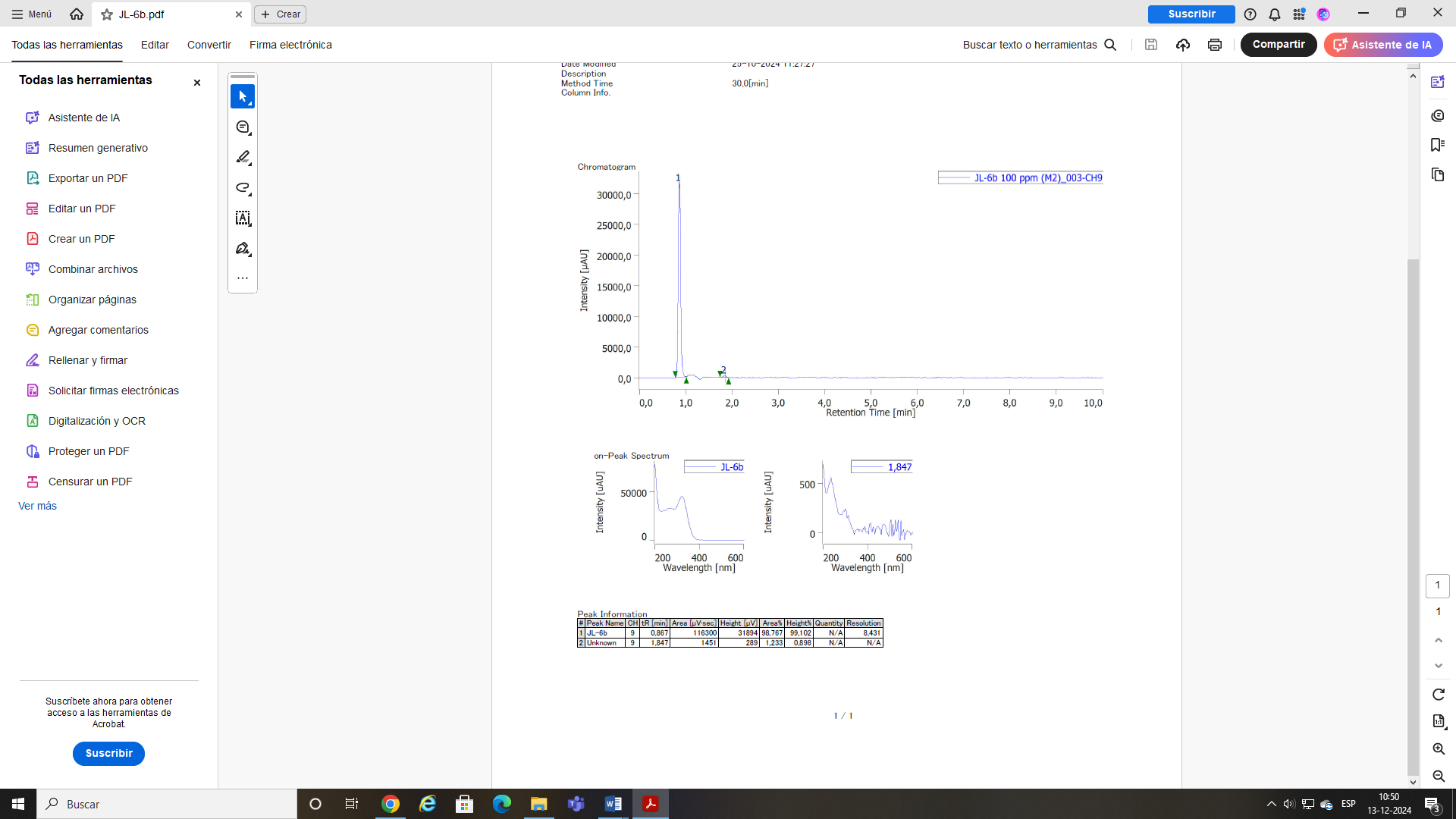


**HPLC compound 6c**


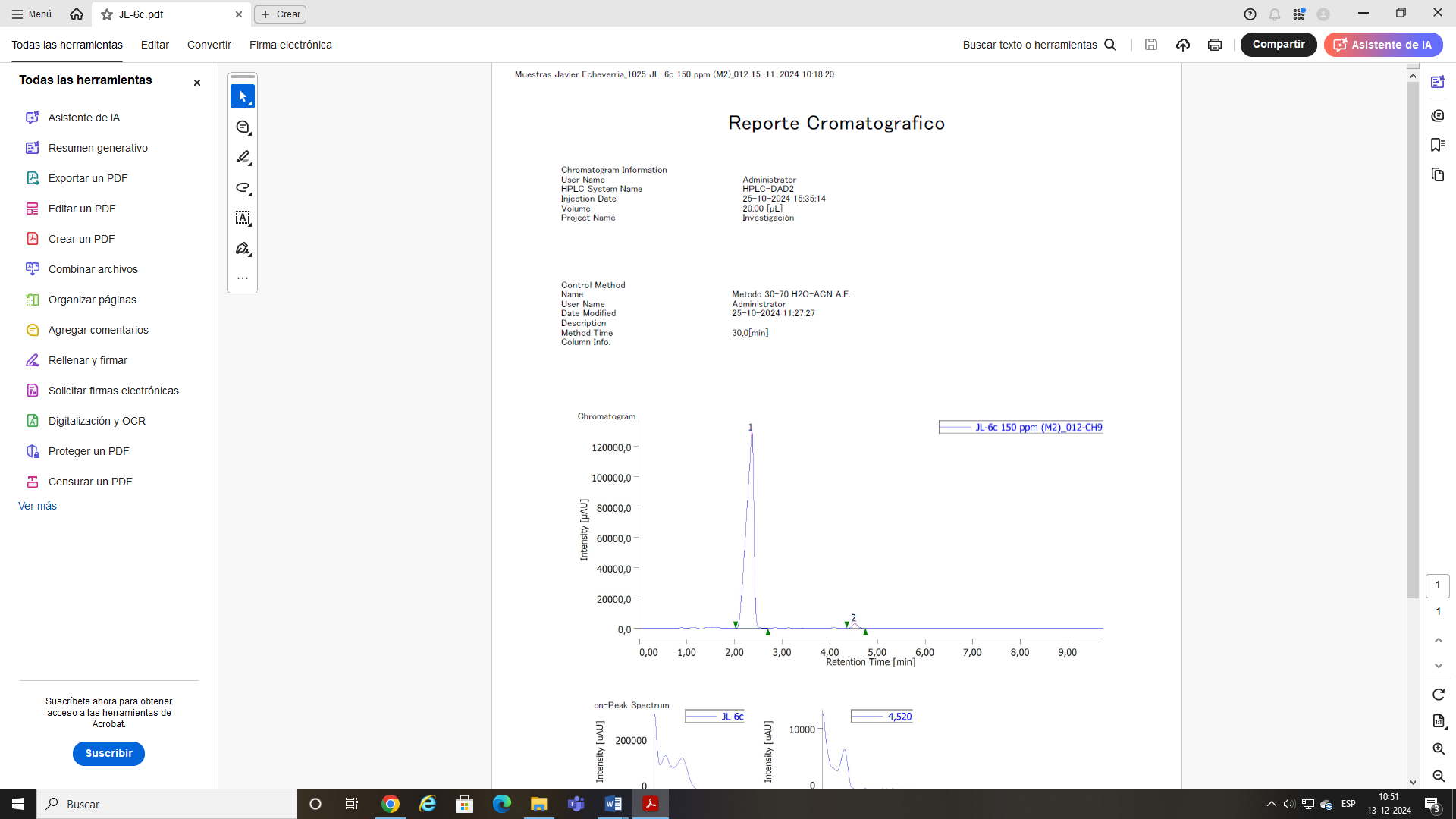


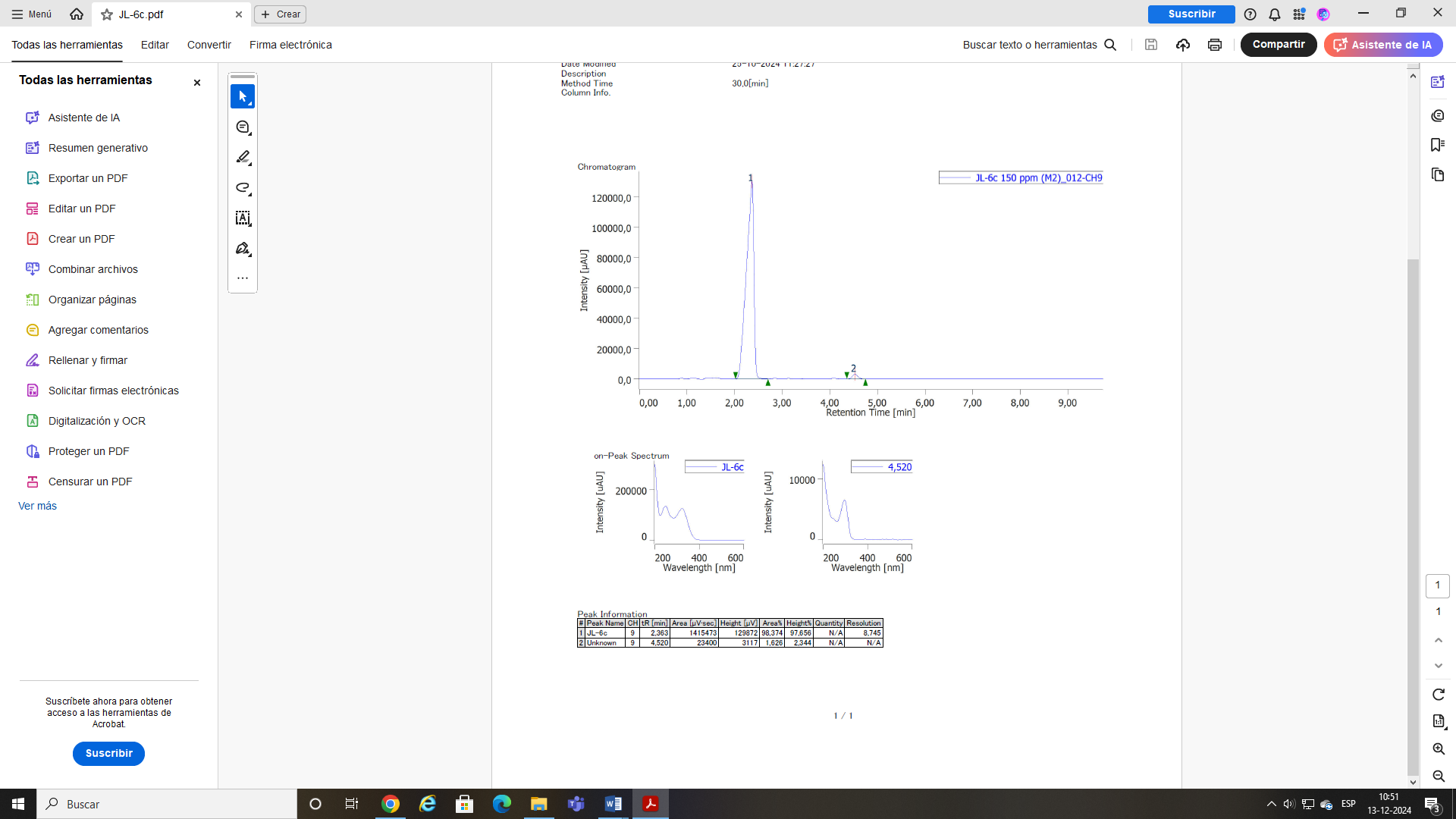


**HPLC compound 6d**


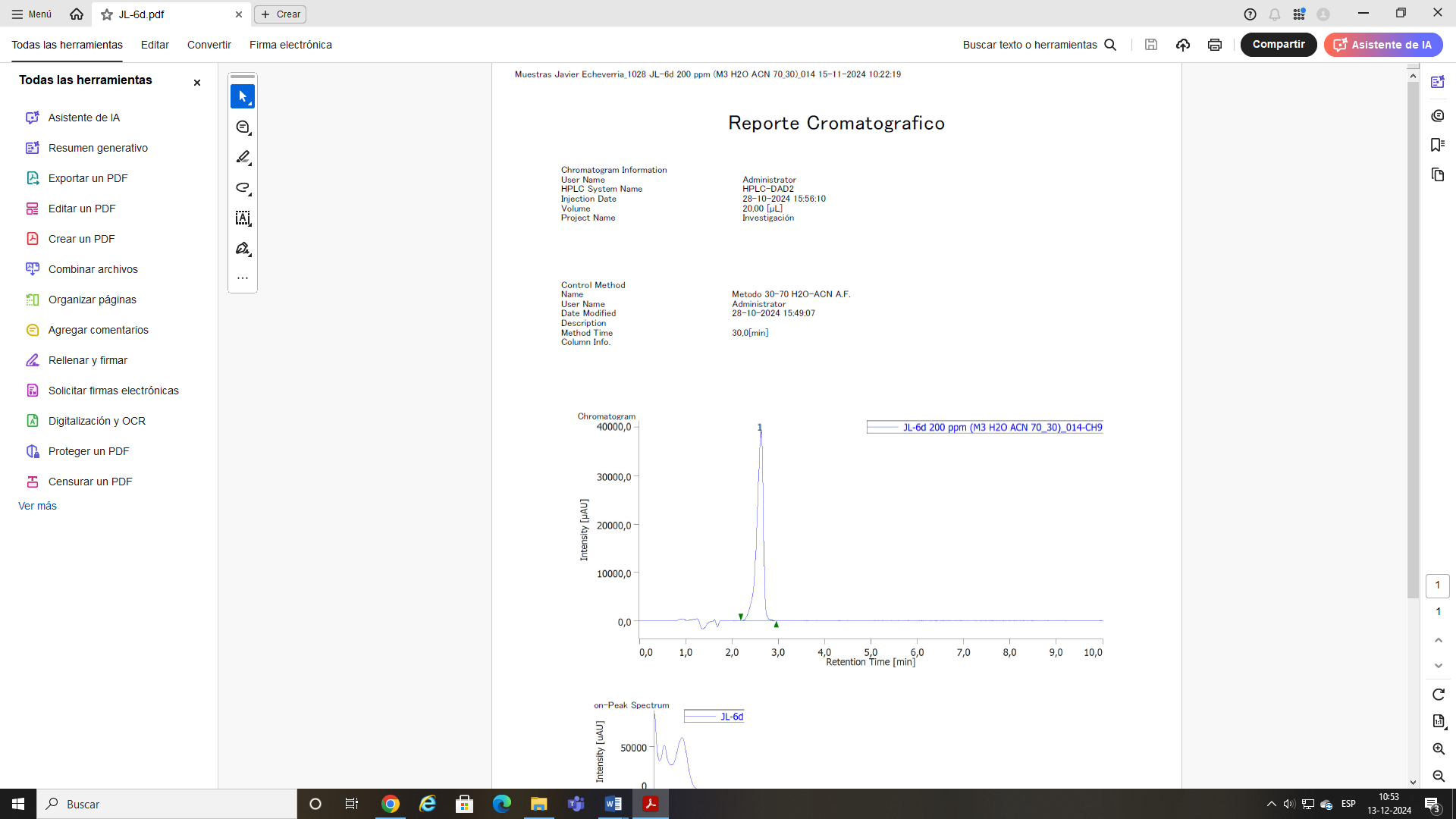


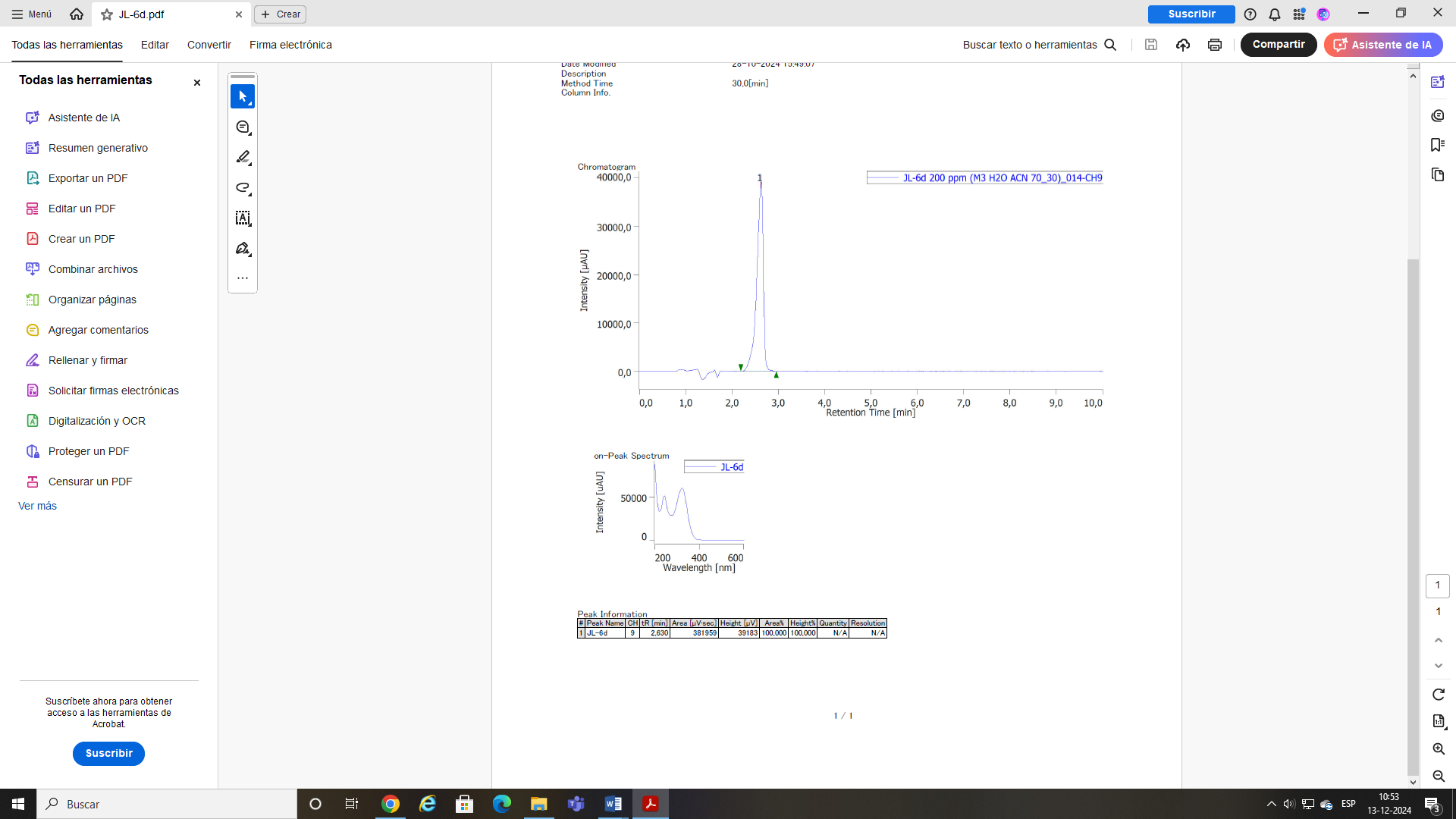


**HPLC compound 6e**


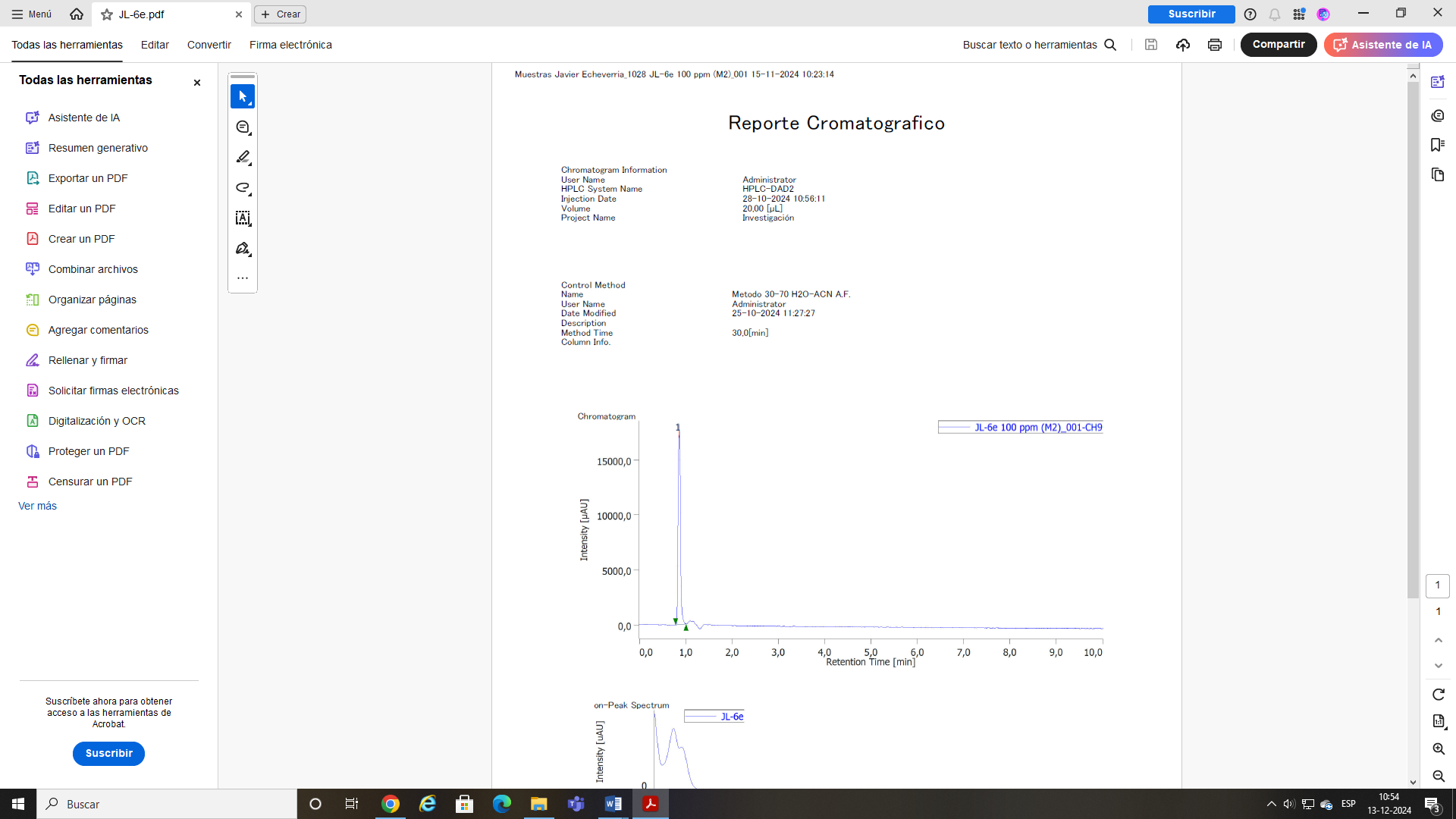


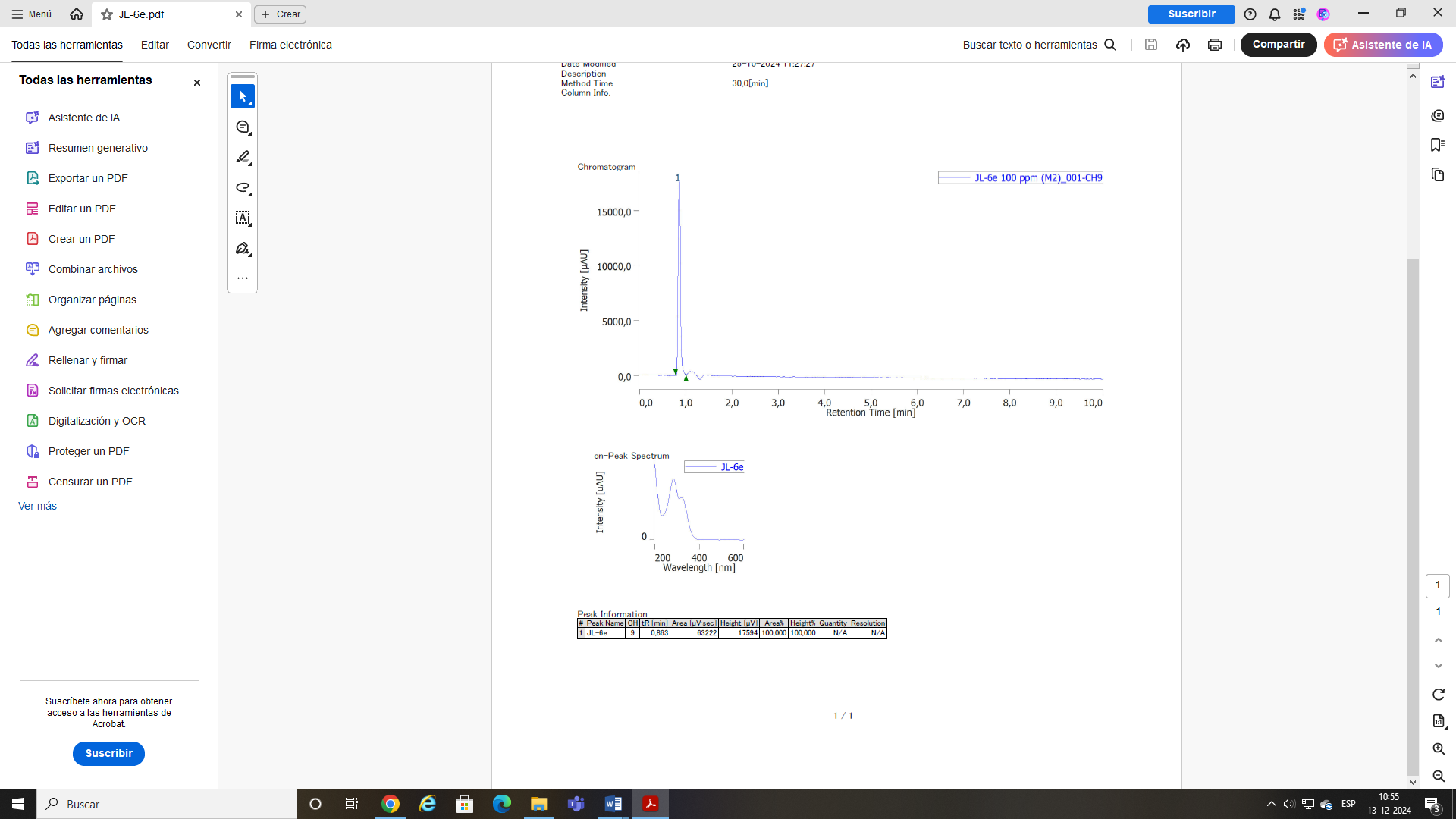


**HPLC compound 6f**


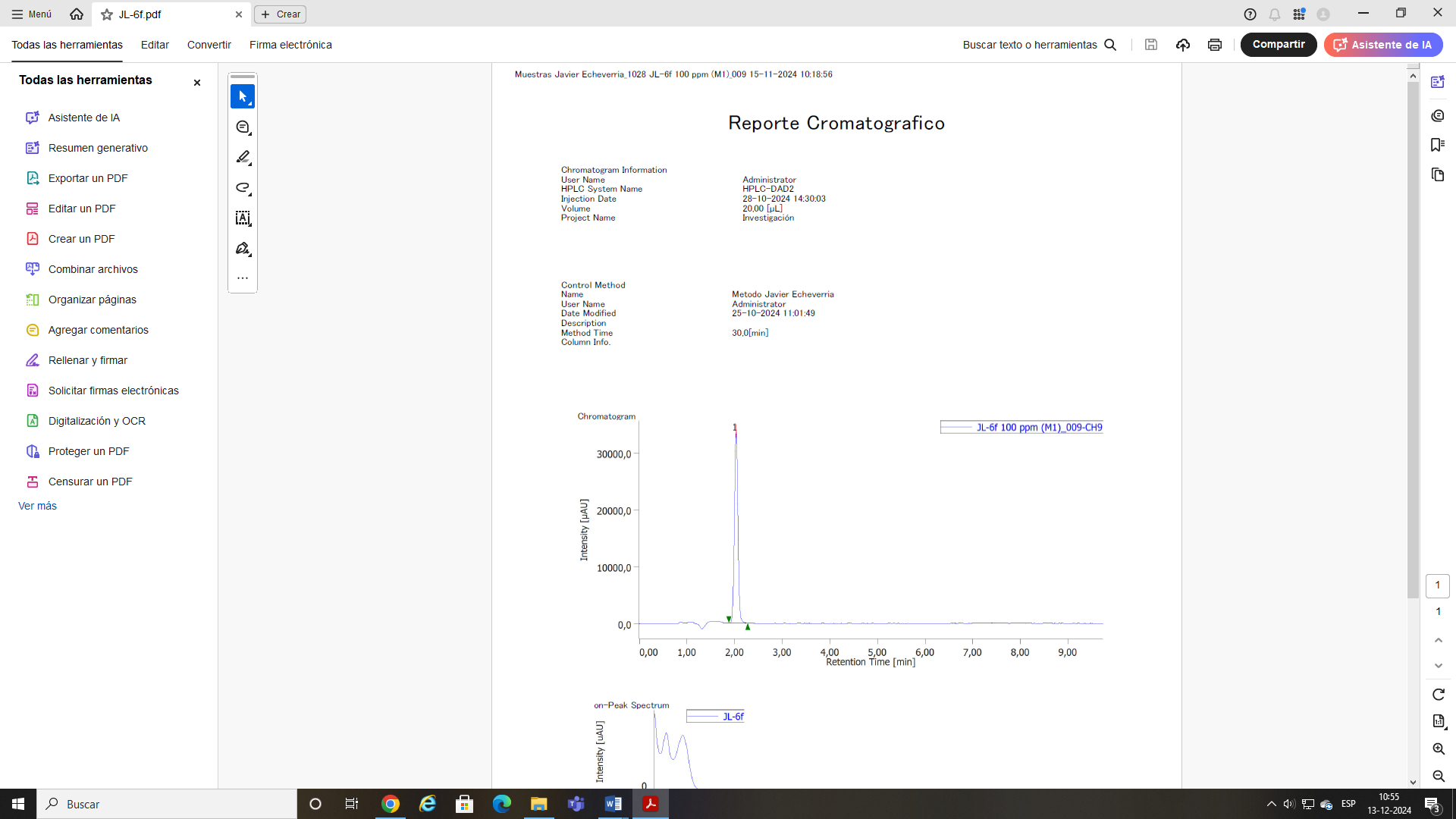


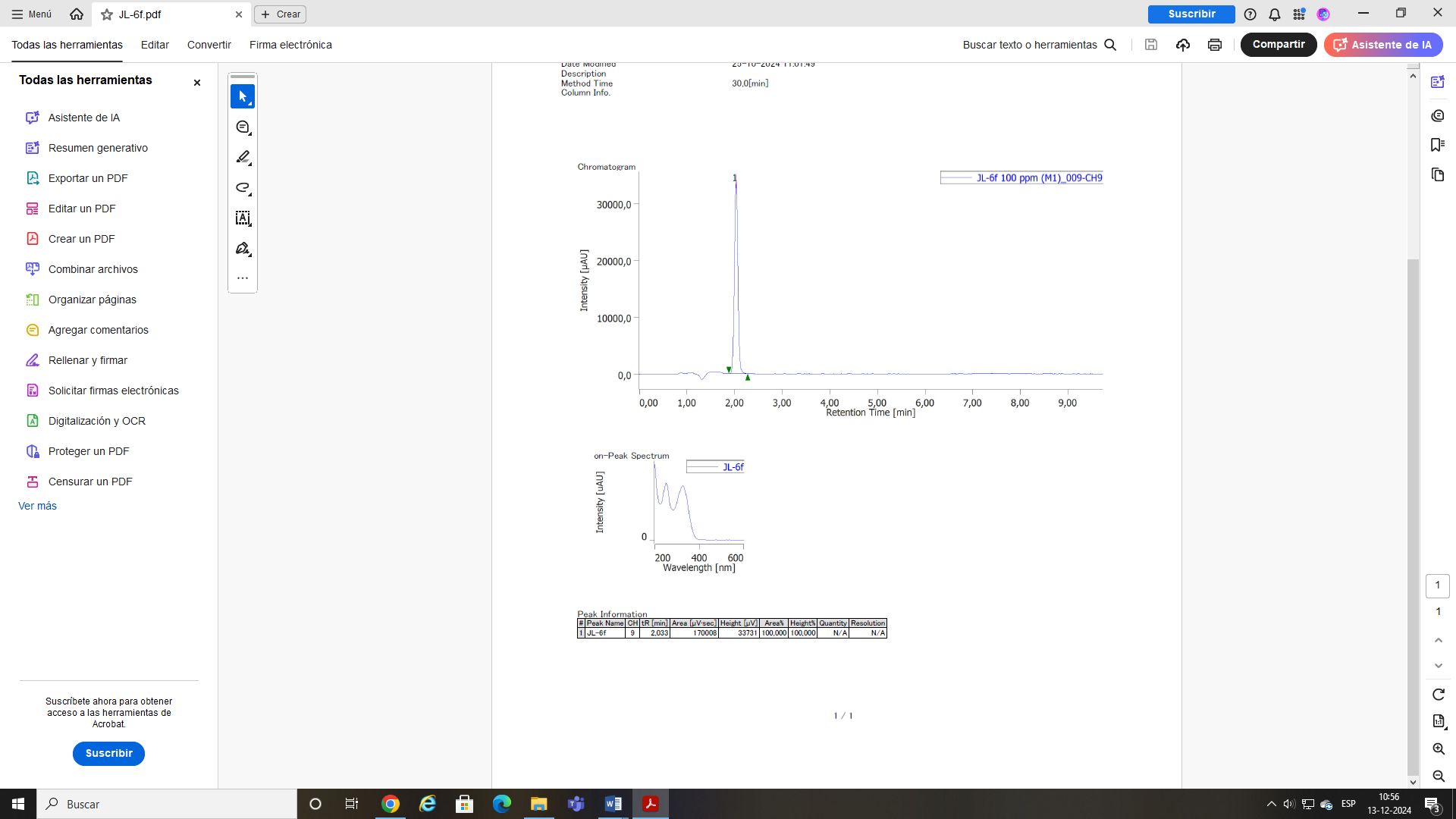


**HPLC compound 6g**


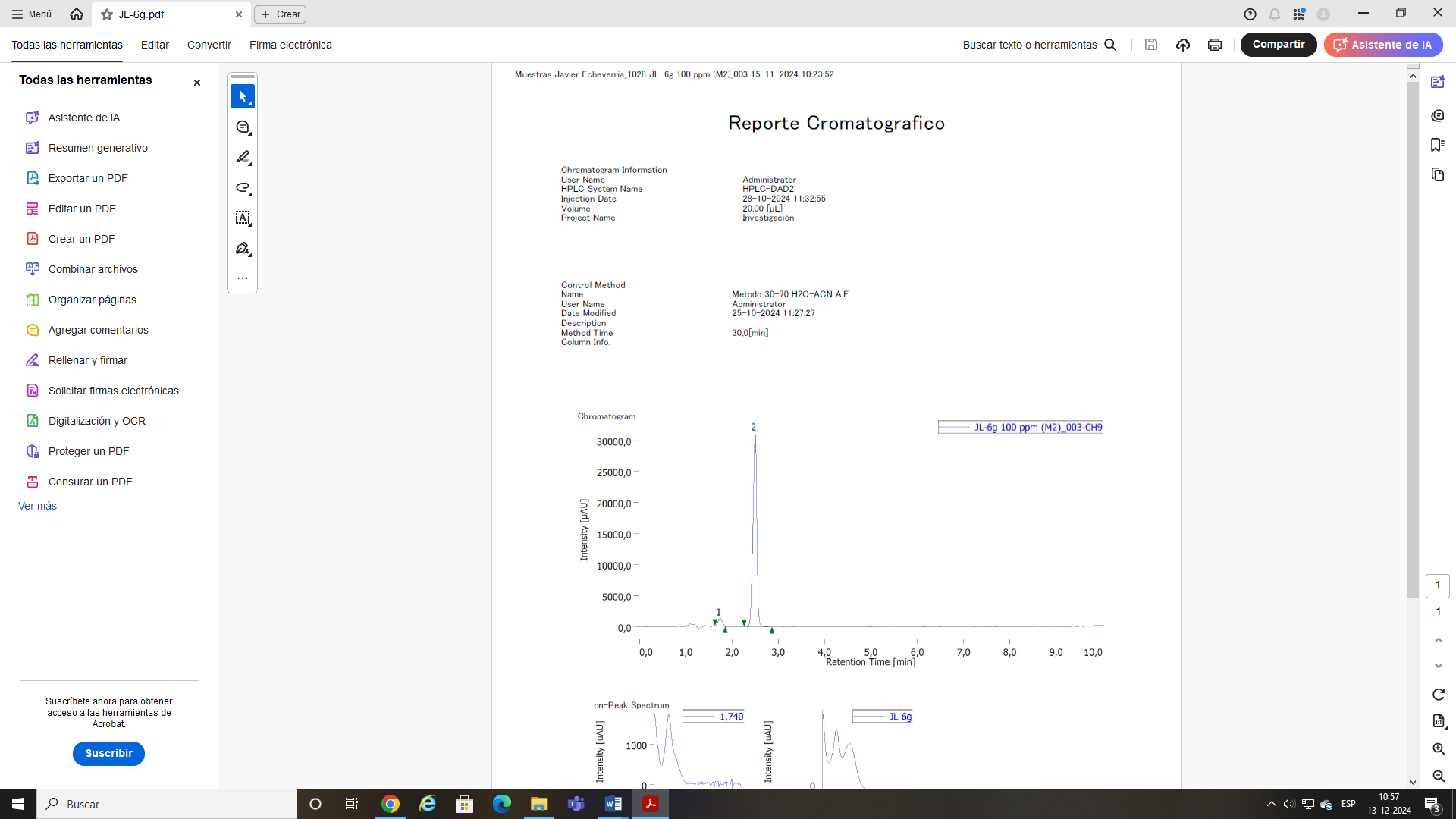


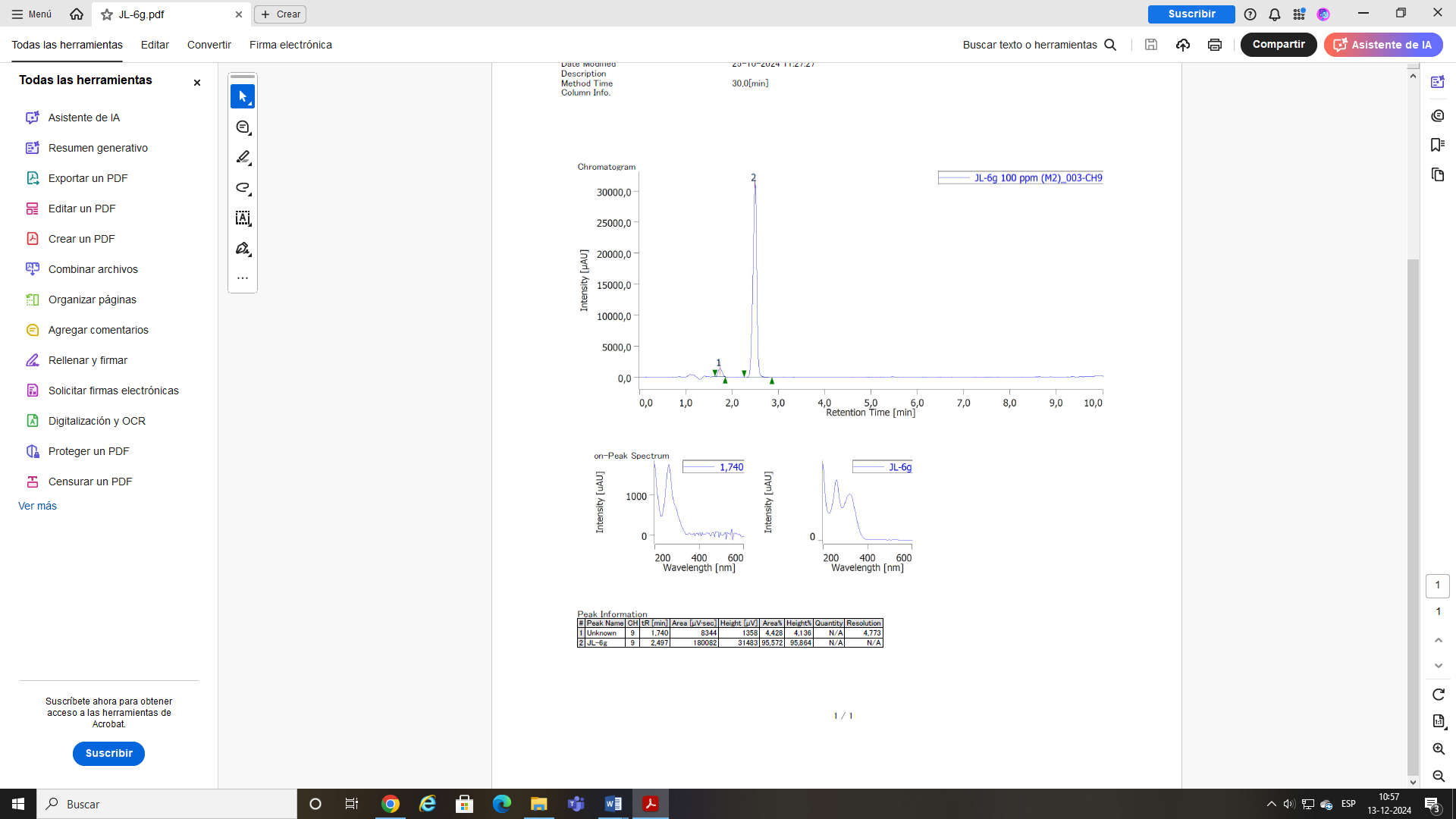


**HPLC compound 6h**


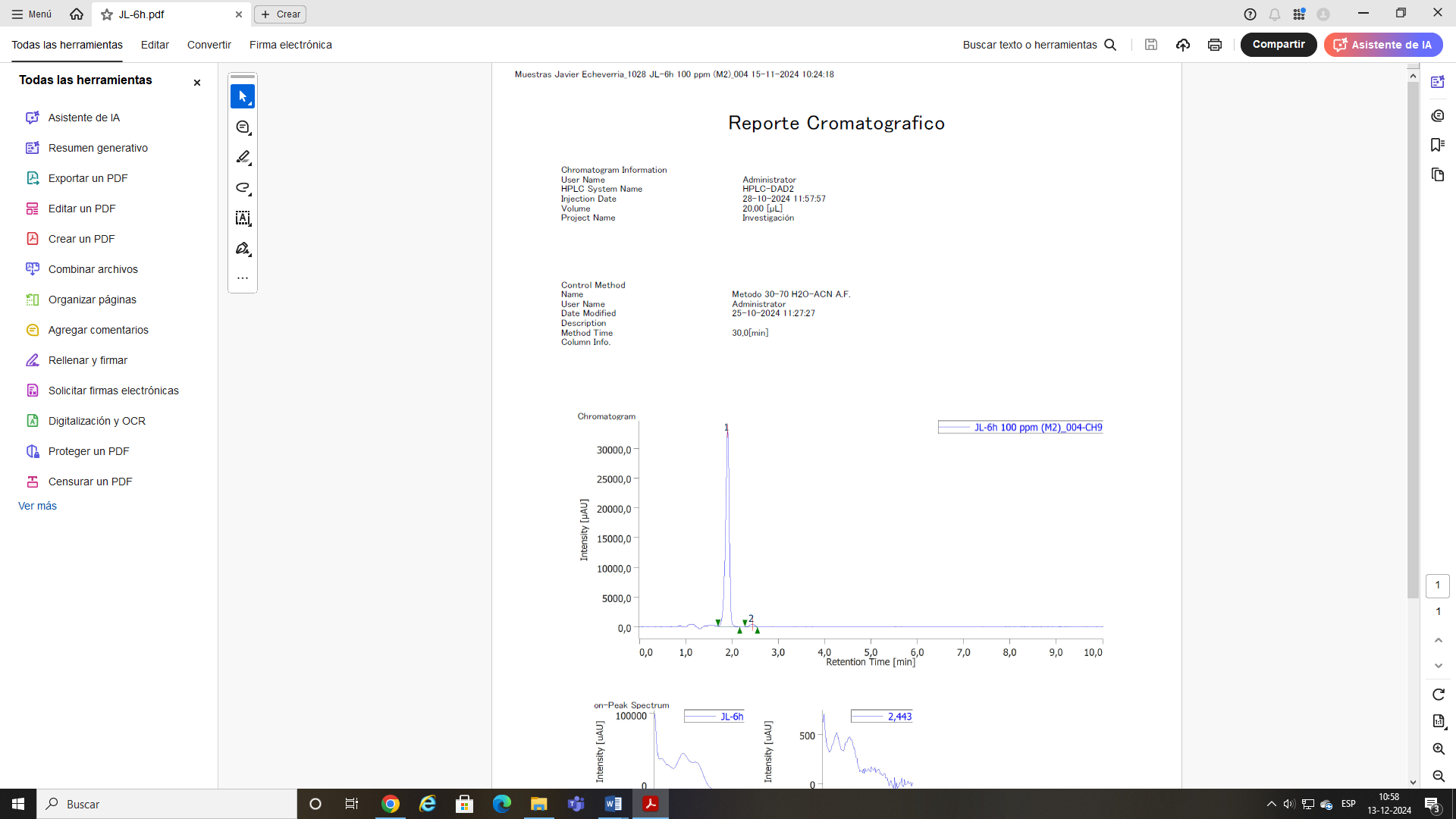


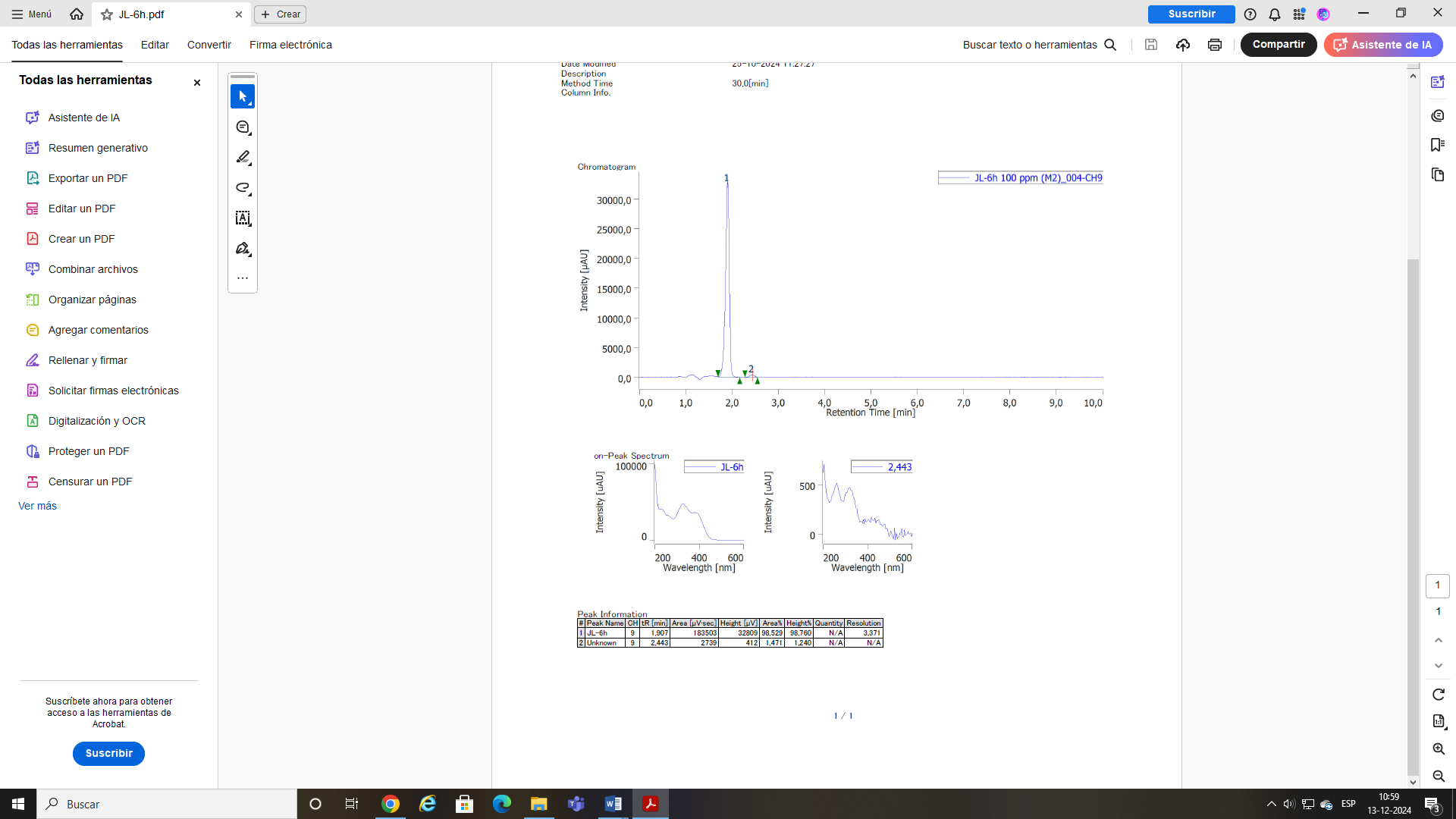


**HPLC compound 6i**


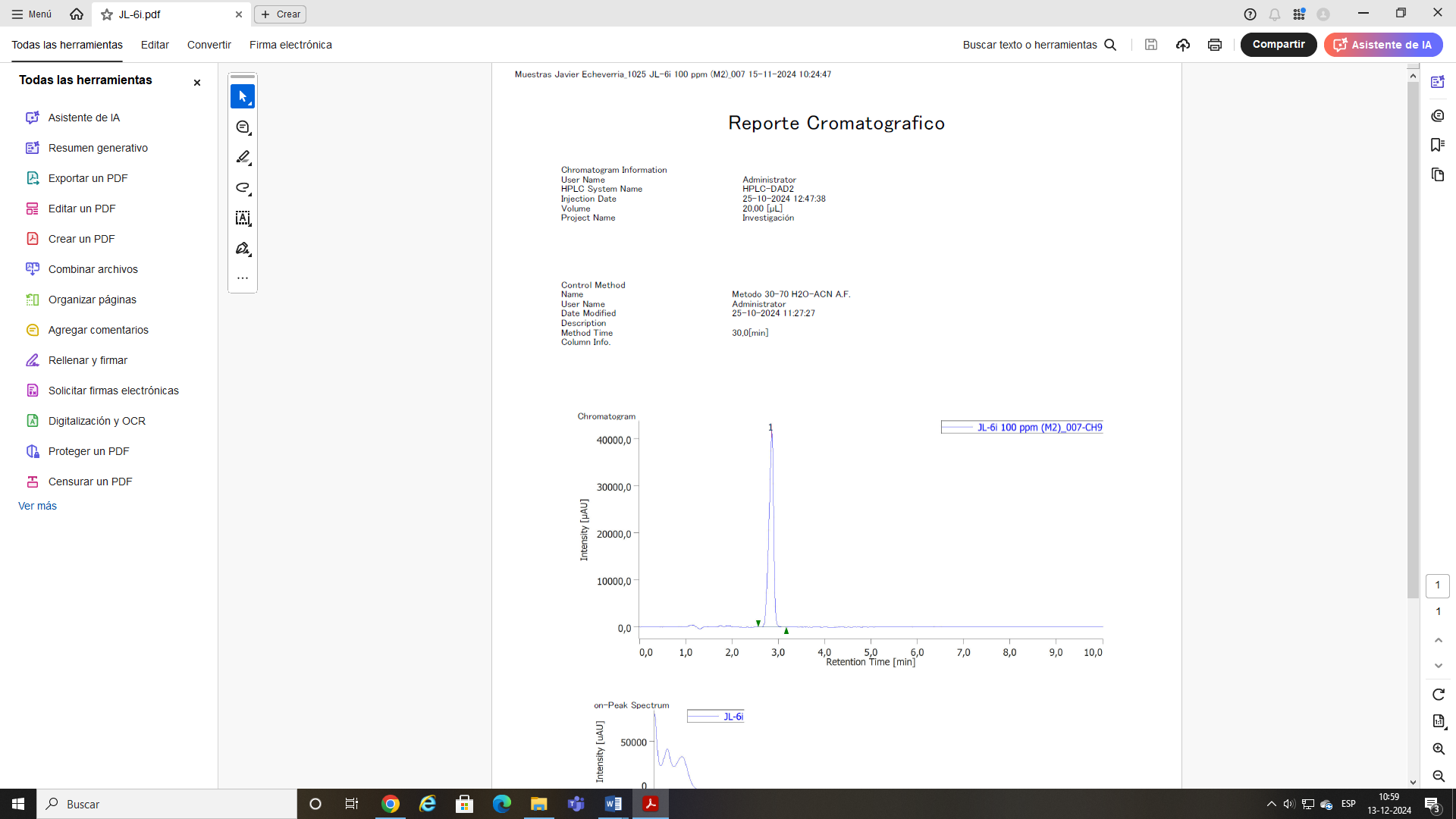


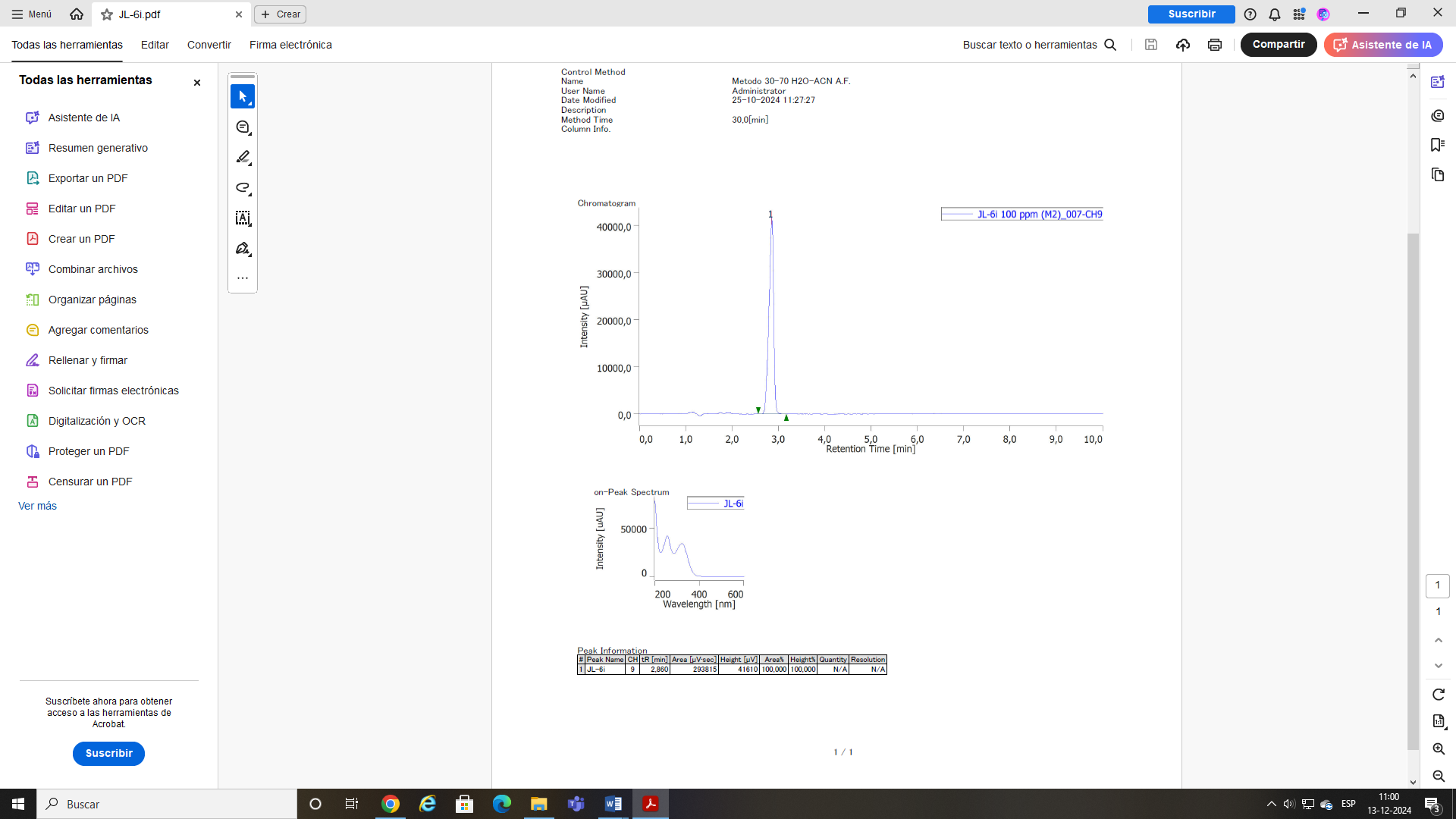


**HPLC compound 6j**


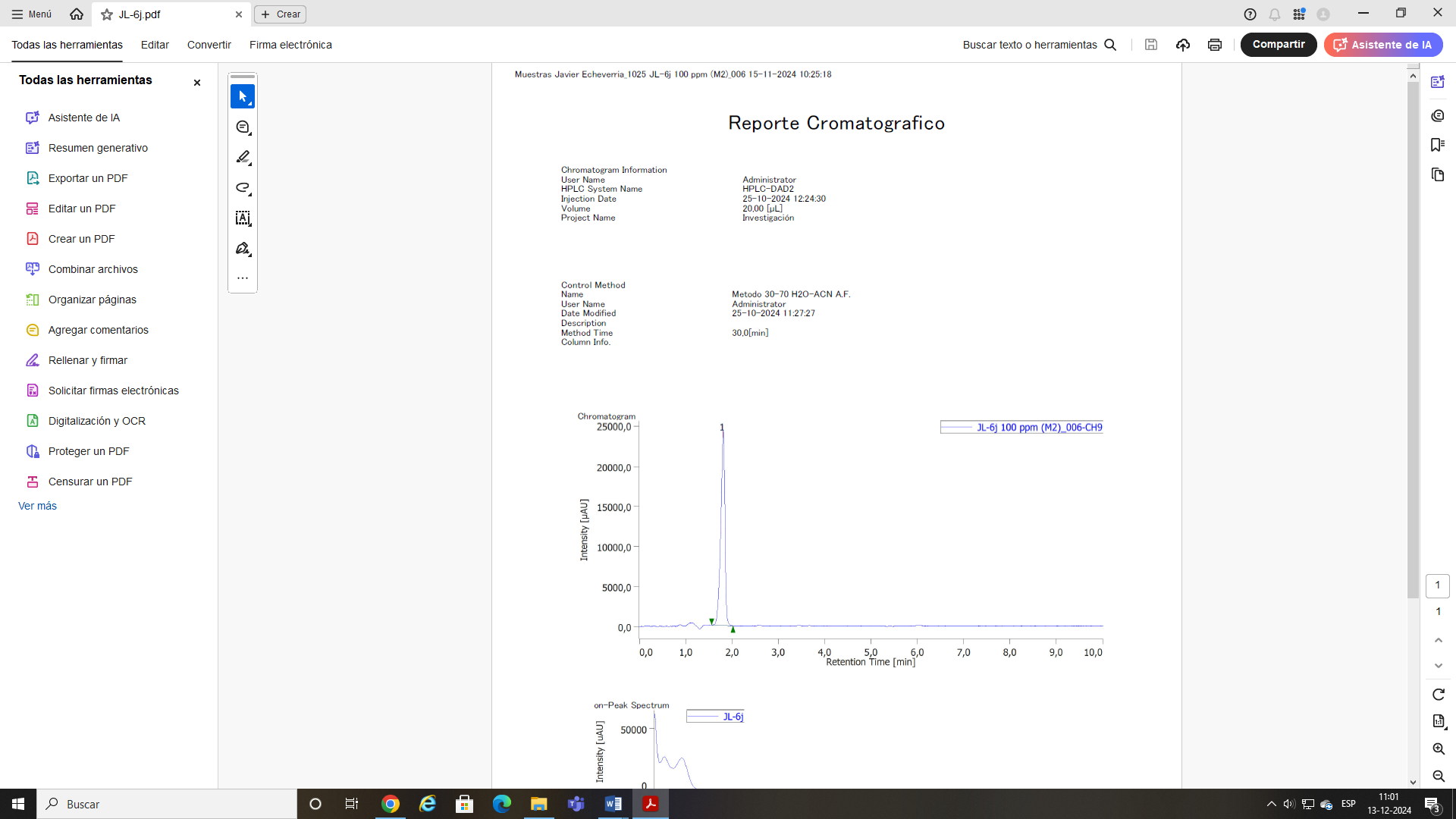


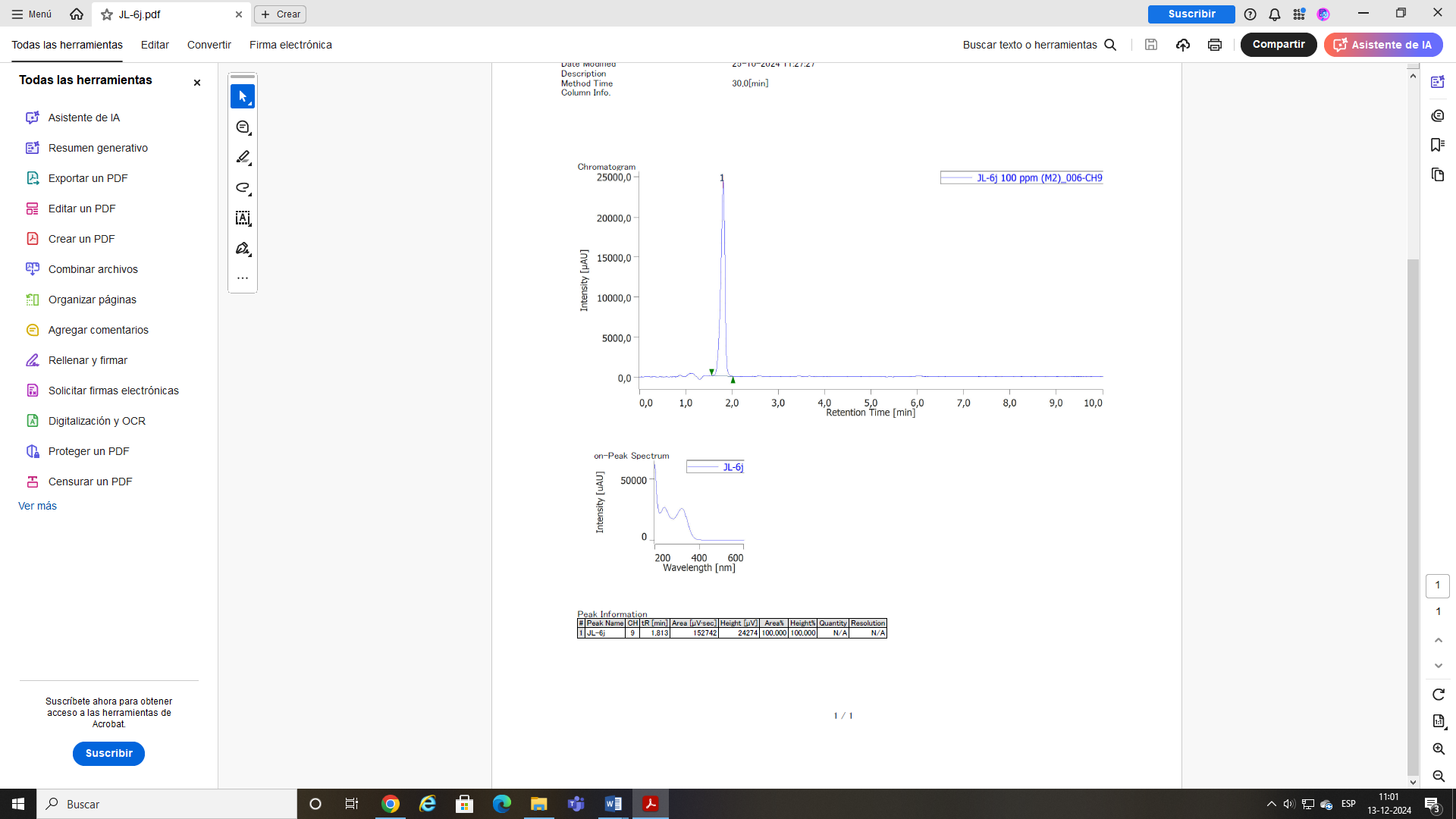


**HPLC compound 6k**

**
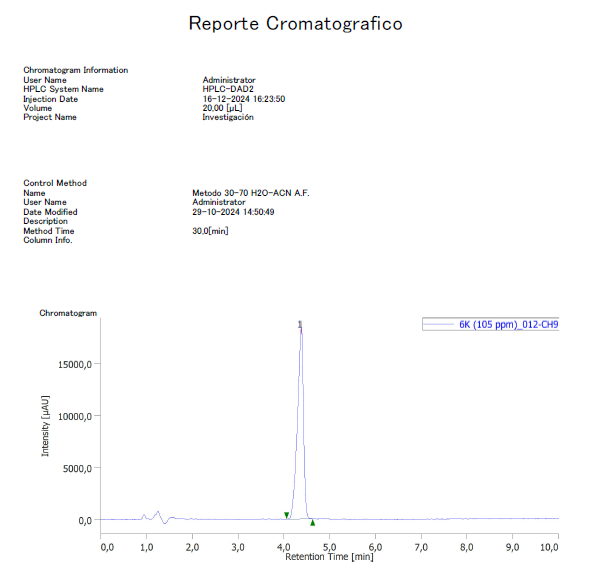
**

**
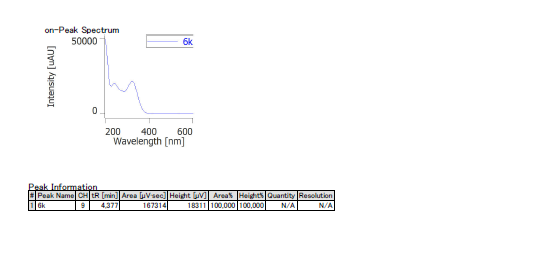
**

**HPLC compound 6l**


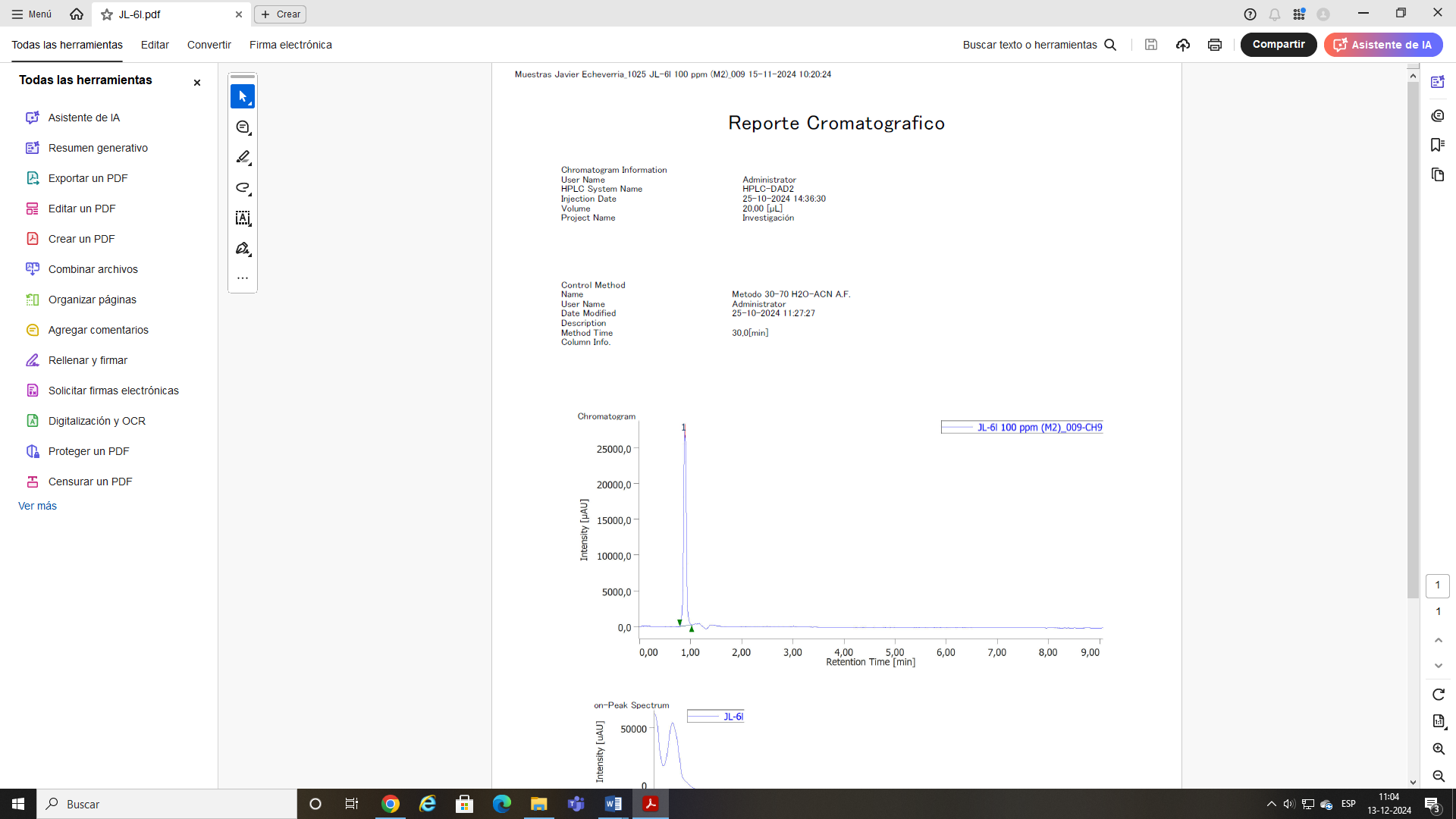


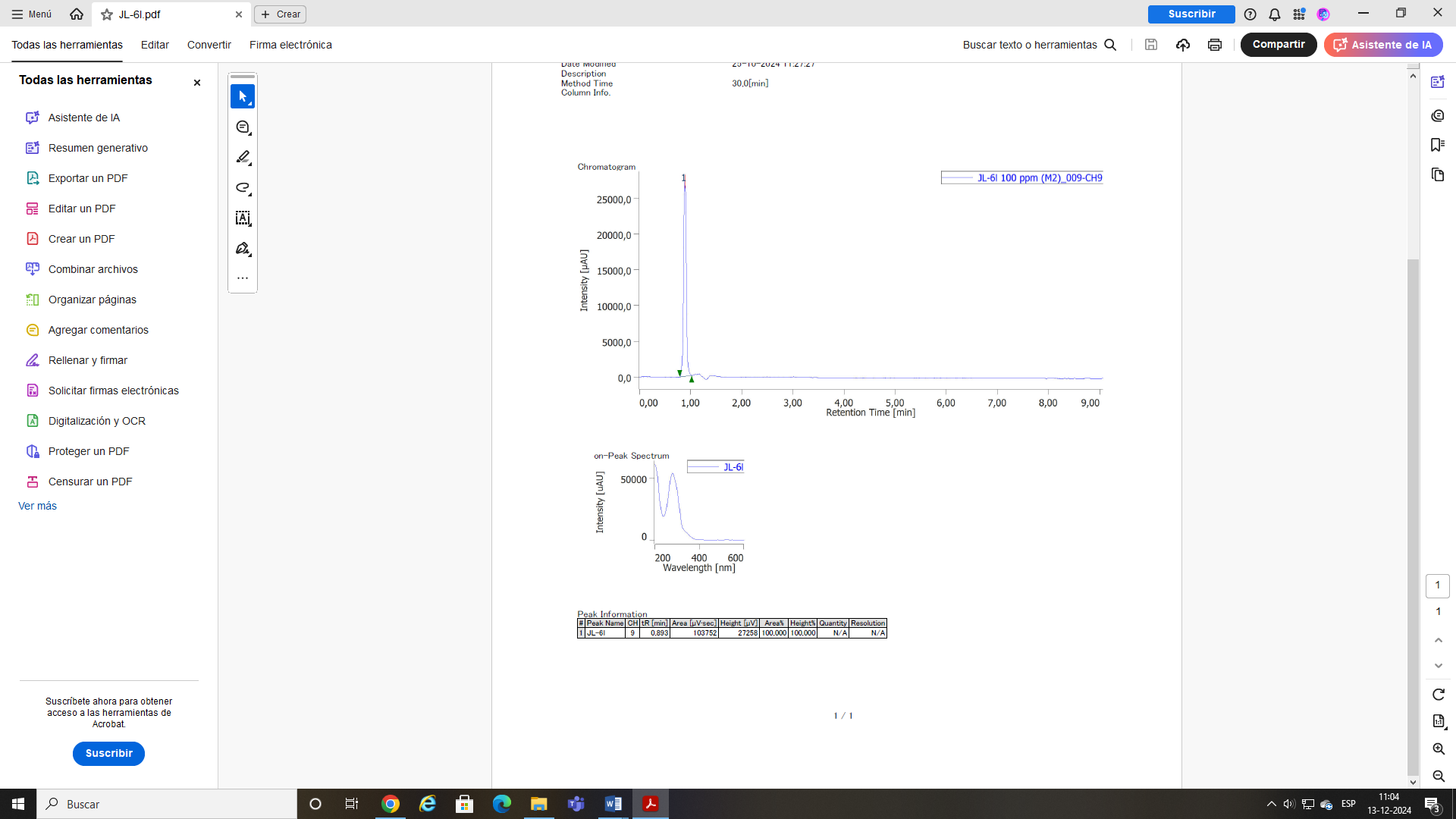


**Table S1.** Selected crystallographic data of compound **6h**.

| **Bond distances** | | |  | **Selected Angles** | | | | |
| --- | --- | --- | --- | --- | --- | --- | --- | --- |
| **Atom 1** | **Atom 2** | **Length (A)** |  | **Atom 1** | **Atom 2** | **Atom 3** | **Angle (º)** |  |
| N3 | C2AA | 1,3639 |  | C2AA | N3 | C10 | 129,88 |  |
| N3 | C10 | 1,4037 |  | C2AA | N7 | C5 | 116,34 |  |
| N1 | C6 | 1,4697 |  | C5 | N8 | C9 | 115,85 |  |
| N1 | C13 | 1,4589 |  | C6 | N1 | C13 | 112,17 |  |
| N1 | C20 | 1,3818 |  | C6 | N1 | C20 | 120,84 |  |
| O6 | C25 | 1,4181 |  | C13 | N1 | C20 | 121,04 |  |
| O6 | C30 | 1,2336 |  | C25 | O6 | C30 | 119,46 |  |
| N4 | C14 | 1,4595 |  | C14 | N4 | C16 | 121,21 |  |
| N4 | C16 | 1,3408 |  | C14 | N4 | C17 | 112,15 |  |
| N4 | C17 | 1,4558 |  | C16 | N4 | C17 | 126,23 |  |
| C3AA | C16 | 1,4991 |  | O1 | N6 | O2 | 123,28 |  |
| C6 | C17 | 1,5035 |  | O1 | N6 | C24 | 118,23 |  |
| C13 | C14 | 1,4989 |  | O2 | N6 | C24 | 118,46 |  |
| C30 | F1 | 1,2706 |  |  | | | |  |
| C30 | F3 | 1,2607 |  | **Selected Torsions** | | | | |
| C30 | F5 | 1,4734 |  | **Atom 1** | **Atom 2** | **Atom 3** | **Atom 4** | **Torsion (º)** |
|  |  |  |  | C10 | N3 | C2AA | N7 | 9,21 |
|  |  |  |  | C2AA | N3 | C10 | C7 | -22,66 |
|  |  |  |  | C13 | N1 | C20 | C26 | 24,03 |
|  |  |  |  | O2 | N6 | C24 | C28 | -5,67 |
|  |  |  |  | C8 | C3AA | C16 | N4 | -131,43 |
|  |  |  |  | N8 | C9 | C18 | C27 | -31,79 |

**Table S2.** Primary and secondary antibodies used in western blot experiments.

| **Primary Ab** | ***Dilution*** | ***Source*** | ***Incubation***  ***time*** | ***Incubation***  ***temperature*** | ***Brand*** |
| --- | --- | --- | --- | --- | --- |
| Caspase-3 | 1:1000 | Rabbit | 2 h | RT | Cell Signaling, MA, USA |
| Caspase-9 | 1:1000 | Rabbit | 2 h | RT | Cell Signaling, MA, USA |
| Caspase-8 | 1:1000 | Mouse | 2 h | RT | Cell Signaling, MA, USA |
| α-Tubulin | 1:5000 | Mouse | 2 h | RT | Sigma-Aldrich, MO, USA |
| Apaf-1 | 1:1000 | Rabbit | 2 h | RT | Abcam, Cambridge, UK |
| Cyt-C | 1:1000 | Rabbit | 2 h | RT | Abcam, Cambridge, UK |
| ERK | 1:1000 | Rabbit | 2 h | RT | Santa Cruz Biotechnology, Inc |
| p-ERK | 1:1000 | Rabbit | 2 h | RT | Santa Cruz Biotechnology, Inc |
| p53 | 1:1000 | Mouse | 2 h | RT | Cell Signaling, MA, USA |
| p-P53 | 1:1000 | Mouse | 2 h | RT | Cell Signaling, MA, USA |

| **Secondary Ab** | ***Dilution*** | ***Source*** | ***Incubation***  ***time*** | ***Incubation***  ***temperature*** | ***Brand*** |
| --- | --- | --- | --- | --- | --- |
| Anti- Mouse HRP | 1:5000 | Goat | 2 h | RT | R&D Systems |
| Anti- Rabbit HRP | 1:5000 | Goat | 2 h | RT | Sigma-Aldrich |

**Ab: antibody, RT: room temperature, ON: overnight.**

**Table S3.** Primary and secondary antibodies used in immunocytochemistry.

| **Primary Ab** | ***Dilution*** | ***Source*** | ***Incubation***  ***time*** | ***Incubation***  ***temperature*** | ***Brand*** |
| --- | --- | --- | --- | --- | --- |
| Bcl-2 | 1:100 | Rabbit | 2 h | 4ºC | Santa Cruz, CA, USA Santa Cruz, CA, USA |
| Caspase-3 | 1:100 | Rabbit | 2 h | 4ºC | Cell Signaling, MA, USA |
| α-Tubulin | 1:500 | Mouse | 2 h | 4ºC | Sigma-Aldrich, MO, USA |

| **Secondary Ab** | ***Dilution*** | ***Source*** | ***Incubation***  ***time*** | ***Incubation***  ***temperature*** | ***Brand*** |
| --- | --- | --- | --- | --- | --- |
| Anti- Rabbit Alexa-594 | 1:250 | Goat | 2 h | 4ºC | Invitrogen, CA, USA  USA |
| Anti- Mouse Alexa-488 | 1:250 | Goat | 2 h | 4ºC | Invitrogen, CA, USA |

**Ab: antibody, RT: room temperature, ON: over night**

**Table S4.** *In vitro* cytotoxicity of compounds **6a-l** on cancer cell lines.

| **Compounds** | **IC_50_^a^** | | | |  |
| --- | --- | --- | --- | --- | --- |
|  | **HL-60** | **HeLa** | **HepG2** | **MCF-7** | **Vero** |
| **6a** | > 50 | > 50 | > 50 | > 50 | - |
| **6b** | > 50 | > 50 | > 50 | > 50 | - |
| **6c** | > 50 | > 50 | > 50 | > 50 | - |
| **6d** | > 50 | > 50 | > 50 | > 50 | - |
| **6f** | 9.1± 5.1(5.5) | > 50 | > 50 | > 50 | > 50 |
| **6g** | 32 ± 2.7 (1.6) | > 50 | > 50 | > 50 | > 50 |
| **6h** | > 50 | > 50 | > 50 | > 50 | - |
| **6i** | > 50 | > 50 | > 50 | > 50 | - |
| **6j** | > 50 | > 50 | > 50 | > 50 | - |
| **6k** | > 50 | > 50 | > 50 | > 50 | - |
| **6l** | > 50 | > 50 | > 50 | > 50 | - |
| **Etoposide** | 3.0 ± 1.1(8.3) | 21.2 ± 3.8 | 34 ± 1.0 (0.7) | 7.6 ± 2.1 | > 25 |

^a^ IC_50_ values were determined in three independent experiments for triplicate in the range of 0.05 to 50 μM.

**Table S5.** Docking protocol validation for VEGFR-2.

| 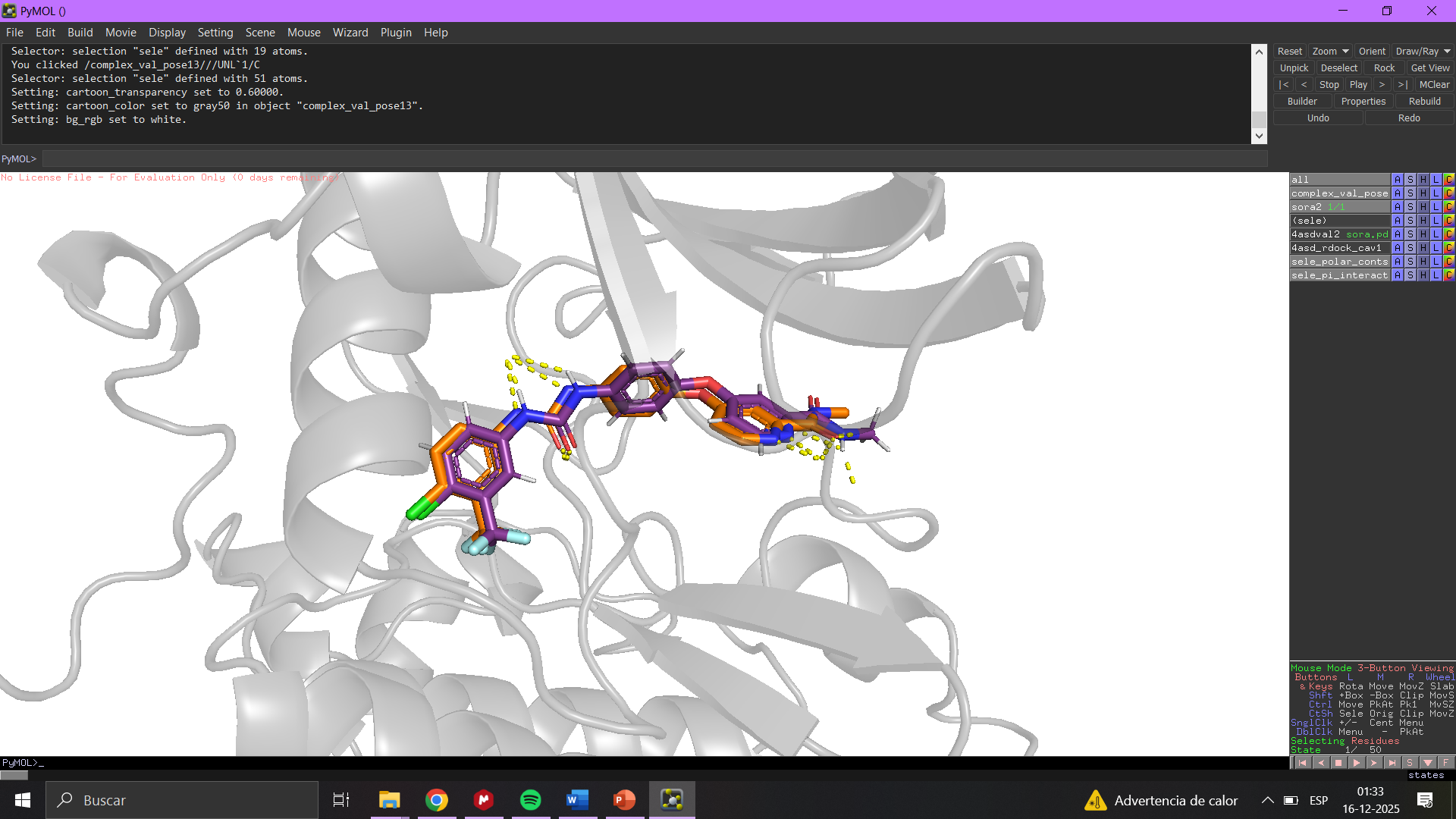  Purple: original ligand co-crystallized  Orange: best-ranked re-docked pose | | | | | |
| --- | --- | --- | --- | --- | --- |
| **Protein** | **PDB ID** | **Ligand co-crystallized** | **RMSD** | **Validation**  **criterion** | **Docking score** |
| VEGFR-2 | 4ASD | Sorafenib | 1.02 Å | RMSD < 2.0 Å | -30.63 |

**Table S6.** Docking protocol validation for B-Raf.

| 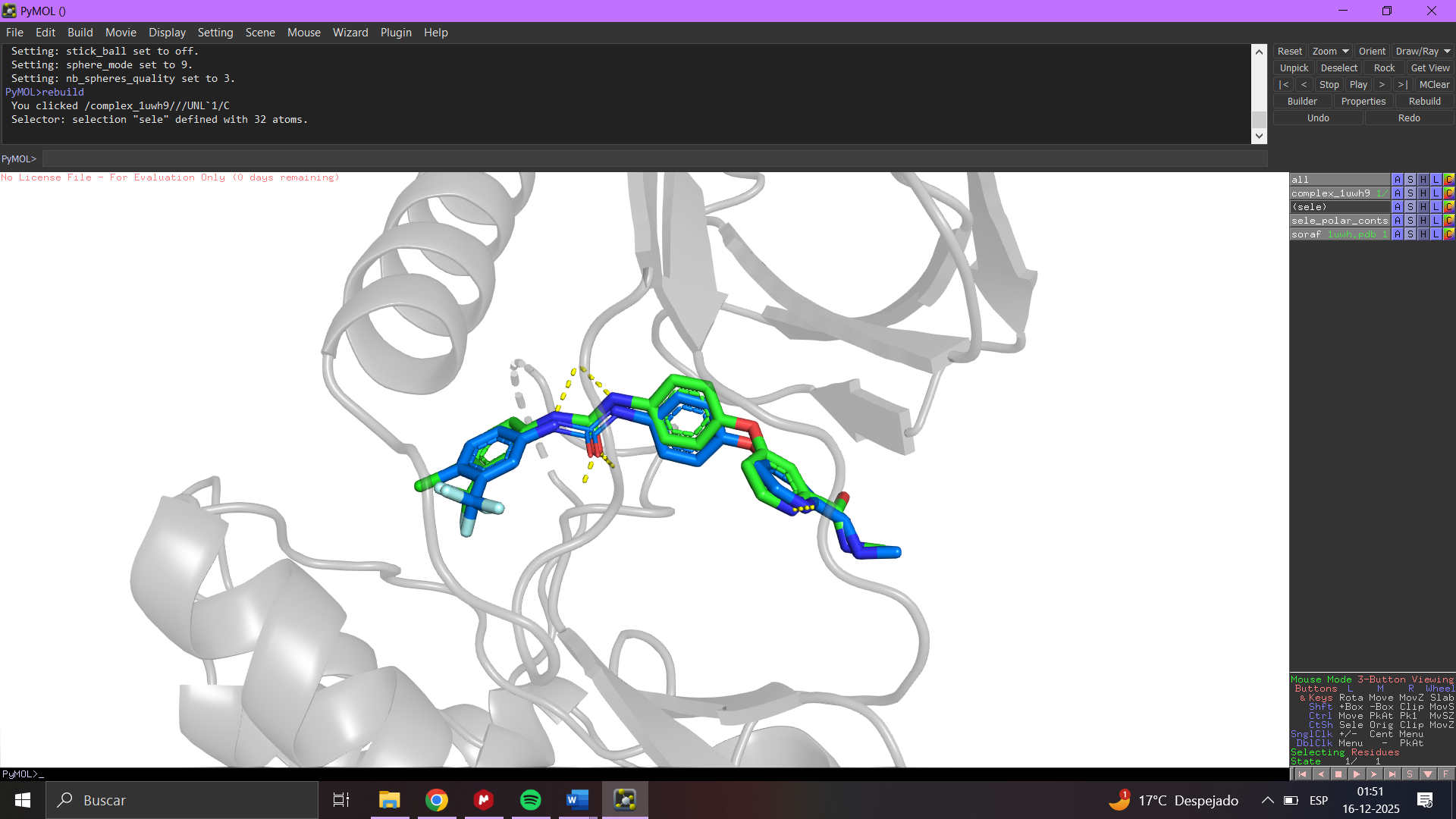  Blue: original ligand co-crystallized  Green: best-ranked re-docked pose | | | | | |
| --- | --- | --- | --- | --- | --- |
| **Protein** | **PDB ID** | **Ligand co-crystallized** | **RMSD** | **Validation**  **criterion** | **Docking score** |
| B-Raf | 1UWH | Sorafenib | 1.17 Å | RMSD < 2.0 Å | -30.61 |


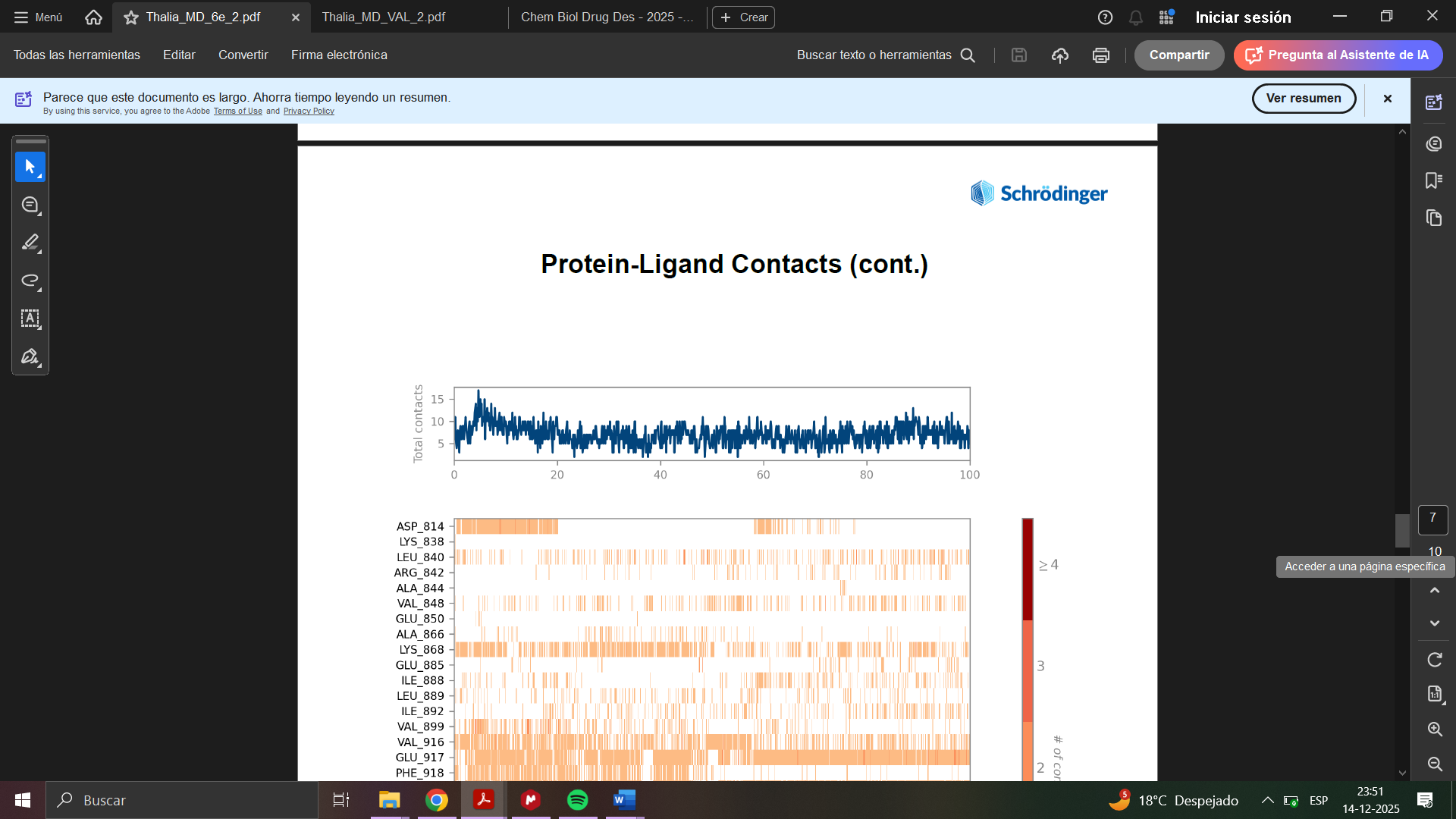


**A**


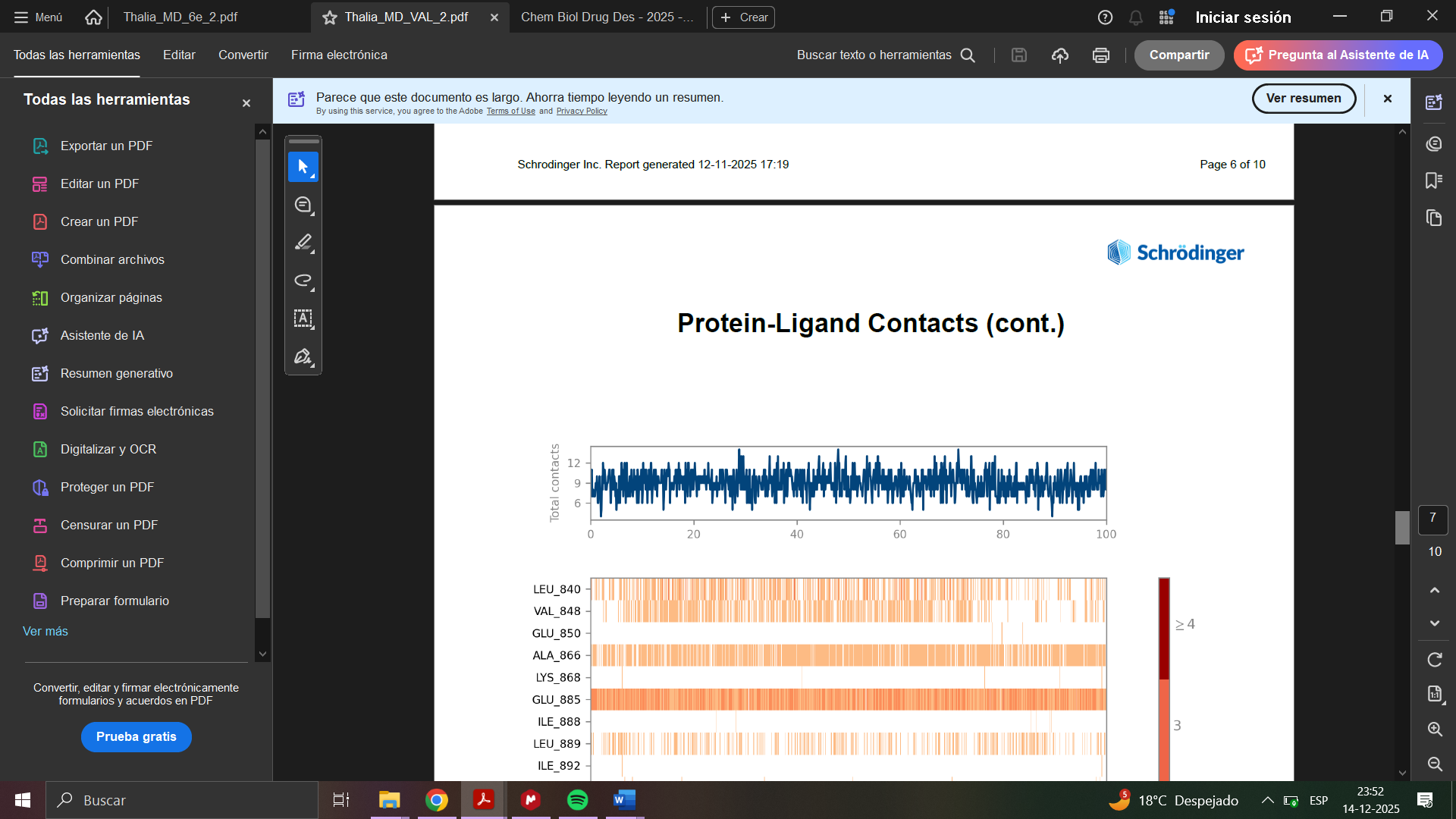


**B**

**Figure S1.** Superposition of the protein-ligand total contacts for **6e** (**A**), and sorafenib (**B**).
